# Influence of common lighting conditions and time-of-day on the effort-related cardiac response

**DOI:** 10.1371/journal.pone.0239553

**Published:** 2020-10-07

**Authors:** Johannes Zauner, Herbert Plischke, Hanna Stijnen, Ulrich T. Schwarz, Hans Strasburger

**Affiliations:** 1 Munich University of Applied Sciences, Munich, Germany; 2 Institute of Physics, Chemnitz University of Technology, Chemnitz, Germany; 3 Institute of Medical Psychology, Ludwig-Maximilians-Universität, Munich, Germany; Universita degli Studi di Bologna, ITALY

## Abstract

Melanopic stimuli trigger diverse non-image-forming effects. However, evidence of a melanopic contribution to acute effects on alertness and performance is inconclusive, especially under common lighting situations. Effects on cognitive performance are likely mediated by effort-related physiological changes. We assessed the acute effects of lighting in three scenarios, at two times of day, on effort-related changes to cardiac contraction as indexed by the cardiac pre-ejection period (PEP). In a within-subject design, twenty-seven participants performed a cognitive task thrice during a morning and a late-afternoon session. We set the lighting at 500 lux in all three lighting scenarios, measured horizontally at the desk level, but with 54 lux, 128 lux, or 241 lux melanopic equivalent daylight illuminance at the eye level. Impedance cardiography and electrocardiography measurements were used to calculate PEP, for the baseline and task period. A shorter PEP during the task represents a sympathetic heart activation and therefore increased effort. Data were analysed with linear mixed-effect models. PEP changes depended on both the light scene and time of day (p = 0.01 and p = 0.002, respectively). The highest change (sympathetic activation) occurred for the medium one of the three stimuli (128 lux) during the late-afternoon session. However, effect sizes for the singular effects were small, and only for the combined effect of light and time of day middle-sized. Performance scores or self-reported scores on alertness and task demand did not change with the light scene. In conclusion, participants reached the same performance most efficiently at both the highest and lowest melanopic setting, and during the morning session. The resulting U-shaped relation between melanopic stimulus intensity and PEP is likely not dependent solely on intrinsic ipRGC stimuli, and might be moderated by extrinsic cone input. Since lighting situations were modelled according to current integrative lighting strategies and real-life indoor light intensities, the result has implications for artificial lighting in a work environment.

## Introduction

Understanding the physiological impact that common lighting conditions have on human readiness to perform is paramount when designing artificial lighting. Light falling onto the retina serves multiple functions beyond those of visual perception, adaptation, and accommodation. First and foremost, light is the most potent *Zeitgeber* for the circadian clock. Light can phase-shift, stabilize, or destabilize circadian rhythms, leading to a wide array of health-related effects [1–3]. Light also affects sleep onset and quality of sleep, melatonin synthesis, mood, and alertness [4–7]. Instrumental to these effects are intrinsically photosensitive retinal ganglion cells (ipRGCs) in the retinal ganglion cell layer [8]. IpRGCs contain the photopigment melanopsin, which is most sensitive to a wavelength at around 480 nm [9]. These ganglion cells project through neural pathways different from those of the visual system and exert so-called *non-image-forming effects* (NIF, also called melanopic effects). The effects can be acute or can take a slower course, over a more extended period of time [10, 11]. When describing the NIF stimulus, the main focus is on ipRGC sensitivity, and, to a lesser degree, on the spatial distribution of light in the visual field [12, 13]. Recent studies help better understand known effects and discover new dependencies of physiological parameters on light. However, to transfer these findings to more common lighting situations, the eye-level stimulus used in the respective studies needs to be realistic with respect to its spatial distribution as well as its intensity. Additionally, the influence of intrinsic and extrinsic stimuli in ipRGC signalling have made it difficult to quantify light characteristics with respect to their melanopic effects. Recent SI-compliant metrics [13] seem to best describe physiological responses under common intensities [14], but are not yet universally adopted. When possible, SI-compliant melanopic illuminance values were calculated for literature references in this paper.

One NIF-effect example worth exploring are acute melanopic effects on the heart, and their connection to alertness and cognitive performance. There are conflicting findings regarding this topic in the current literature. For a critical review of how daytime alertness and mood depend on a melanopic stimulus, see Pachito et al. [15].

Changes in colour-temperature conditions lead to a change of effort-related cardiac response intensity (see below). In a between-subject design, Lasauskaite and Cajochen [16] tested 74 young participants under differing lighting conditions during daytime. The hypothesis of the researchers stated that higher correlated colour temperature would increase alertness (mediated mainly by ipRGCs), and therefore readiness to perform. This, in turn, should lead to a lower experienced task demand and, therefore, effort. The experimental, white LED light was set at four combinations of colour temperature and melanopic intensity, 2800 K / 301 lux, 4000 K / 415 lux, 5000K / 454 lux, or 6500K / 563 lux (correlated colour-temperature / melanopic equivalent daylight [D65] illuminance, abbreviated as MEDI). A vertical, uniformly illuminated front panel provided the light stimulus, emitting about 500 lux at the eye level, regardless of the spectrum. At the beginning of the test procedure, the test subjects were asked to read a simple text for 15 minutes, after which a demanding cognitive task was carried out. Task performance and self-reported task demand did not vary between the four lighting conditions. Cardiac pre-ejection-period (PEP) reaction as a marker for sympathetic nervous system activation and the experienced task demand were shown to vary. Under 4000K lighting, sympathetic activation was highest. At 6500K, sympathetic activation was lowest, with no change to the PEP baseline on average. The authors conclude that working under cooler light might lead to a lower effort, while leading to the same performance. This would be important for real life performance contexts, like office and school work. The construct of *effort* in the context of cardiovascular response reflects the integration of the *active-coping theory* of Obrist [17] with the *motivational intensity theory* of Brehm and Self [18]. This combination can be summarized as stating that the magnitude of the effort that a person invests in instrumental behaviour depends on the experienced demand, provided that success is regarded by that person as both worthwhile and possible. The mobilized effort is proportional to the task demand, with psychophysiological moderators that influence the perception of *necessary* and *justified* action. The mobilized effort manifests itself, e.g., in an activation of the sympathetic nervous system [19]. Sympathetic activation, in turn, can be indexed by non-invasive electrophysiological measures of cardiac reaction [20]. The advantage of this concept is that *effort* can be indexed through an objective psychometric marker.

While the above-mentioned study is the first (and to date only) one to show this dependency of pre-ejection period changes on light, there are two recent notable exploratory studies. Prayag et al. [21] looked at twenty-eight male participants in a within-subject design. They compared two white-LED-light setups with 254 lux and 100 lux MEDI, respectively, testing each twice for 50 minutes, in the evening between 7:00 pm and 11:00 pm. They conducted several physiological measurements and performance tests, and obtained self-reports. Various NIF responses were found, with influences of light on cardiac parameters (heart rate and heart-rate variability) after two minutes of light onset. Heart rate and electroencephalogram markers for alertness increased equally in both lighting scenarios. Heart-rate variability depended on the lighting situation, with an increase in low-to-high-frequency ratio for the first episode of the lower melanopic stimulus, but not the second. According to the authors, this indicates a more pronounced sympathetic influence on the sympathovagal balance. No changes in cognitive performance were found when comparing the experimental episodes. The second study, by Scholkmann et al. [22], used a monochromatic light screen with intermittent 20-sec light pulses during a 10-minute phase. The LED light was kept at 20 lux at eye level, for 682 nm (red), 515 nm (green), and 465 nm (blue) light, which translates to about 0.01 lux, 28 lux, and 230 lux MEDI, respectively. Fourteen volunteers participated in this within-subject experiment; cerebral hemodynamics and cardiorespiratory activity during midday were assessed. Blue light was shown to stimulate the pre-frontal cortex significantly more than red and green light, implying the presence of a pathway for higher autonomic functions through blue light, according to the authors. For heart rate variability, red light elicited an increase at high frequencies (indicating a stronger vagal modulation), while green light decreased the low-to-high-frequency ratio (indicating a stronger parasympathetic influence on the sympathovagal balance, according to the authors). While the studies by Prayag et al. [21] and Scholkmann et al. [22] showed a varying impact on heart rate variability, the autonomous activity coupled with that is not evident, since it is in dispute whether the low-to-high-frequency ratio accurately measures the cardiac sympathovagal balance [23]. The studies described above show a definitive impact of light on the heart, in particular when compared to dim light (not described here). However, how pronounced any downstream effects of melanopic stimuli on sympathetic heart activation are, and whether these effects are activating or inhibitory, is still inconclusive.

The purpose of the present study was to test whether the reported results by Lasauskaite and Cajochen [16] can be replicated under more common lighting conditions, and under the current knowledge of factors determining the melanopic stimulus. The experimental setup in that study used a participant-to-light-source geometry and level of brightness at the eye that are unlikely to be found in a real-world lighting scenario. Our study was carried out in a university study room with an LED lighting installation that enabled different light intensities, light-incident angles, and light spectra. Since other NIF effects are best predicted by the intrinsic ipRGC stimulus [14], we hypothesized that, with variations to the melanopic stimulus at eye level but constant illuminance on the task area (desk), the effort-related cardiac response would show a decrease when stimulus intensity for the ipRGCs rises. Furthermore, interaction effects of a light stimulus with time of day are almost ubiquitous for NIF effects, such as circadian phase changes, Melatonin suppression, and the post-illumination pupil reflex (PIPR). In those cases, the same light/melanopic stimulus at different times of day/night changes the intensity of the effect [24, 25] or even leads to the opposite effect [26]. Additionally, it is reported that people are more alert and that cognitive tasks are more easily carried out early after sleep, compared to later in the day when sleep load accumulates, with the variations depending on chronotype [27]. Following the reasoning of Lasauskaite and Cajochen [16] on the effect of alertness on perceived task demand, mobilized effort, and cardiac reaction, we thus further hypothesized that the effort-related cardiac response is higher later in the day, compared to early after sleep, and that time of day will interact with the light stimulus.

## Materials and methods

### Participants and study design

Twenty-seven young and healthy volunteers participated in the study (14 females and 13 males; average age: 26.2 ± 4.3 years; Morningness-Eveningness Score: 53.1 ± 10.5 [28]; body mass index, BMI: 23.2 ± 2.8). Based on effect sizes from Lasauskaite and Cajochen [16], we aimed at a sample size of thirty (G*Power 3 [29], with repeated-measures and within-factors setting: effect size: middle, power: 0.8), but were only able to recruit twenty-seven participants during the time window of the study. Participants were paid EUR 30 each (equivalent to USD 33) for taking part. Participants were recruited through messages on bulletin boards and announcements at classes at the Munich University of Applied Sciences. Exclusion criteria for participants were cardiovascular diseases, epilepsy, or antidepressant medication. Participants were instructed to refrain from sport and caffeine intake before experimental sessions. We used a 2×3 within-subject experimental design with two time-of-day scenarios, each comprising three lighting scenarios (see below). Each participant took part in every scenario. To account for learning effects for the cognitive task (especially when done three times in a row), and for potential carry-over effects of light from one scene to the next, the order in which time-of-day and lighting scenarios were tested was randomized for each participant. Cardiovascular data of two participants at evening sessions were lost due to technical problems, resulting in a final sample of 160 measurements (27×2×3 -2) of pre-ejection period (PEP), heart rate (HR), and left ventricular ejection time (LVET), in 27 participants.

### Experimental conditions–lighting and time-of-day

The study was carried out in a learning/study room at the Munich University of Applied Sciences with a dynamic lighting installation that allows varying light intensities, light angle, and light spectrum. Experimental sessions took place from the end of November to February, at two times-of-day: morning and late afternoon. Morning sessions started at 07:00 am, before dawn, late-afternoon sessions at 5:00 pm, after dusk. To eliminate the possibility of residual/dawning daylight or streetlight having an impact on the measurements, window blinds were drawn shut before each session (τ_vis, blinds_: 0). Measurements during the morning session thus took place mainly between 7:30 am and 8:30 am, which corresponds to the beginning of a workday; measurements during the late-afternoon session took place mainly between 5:30 pm and 6:30 pm, corresponding to the end of a workday.

**Fig 1:**
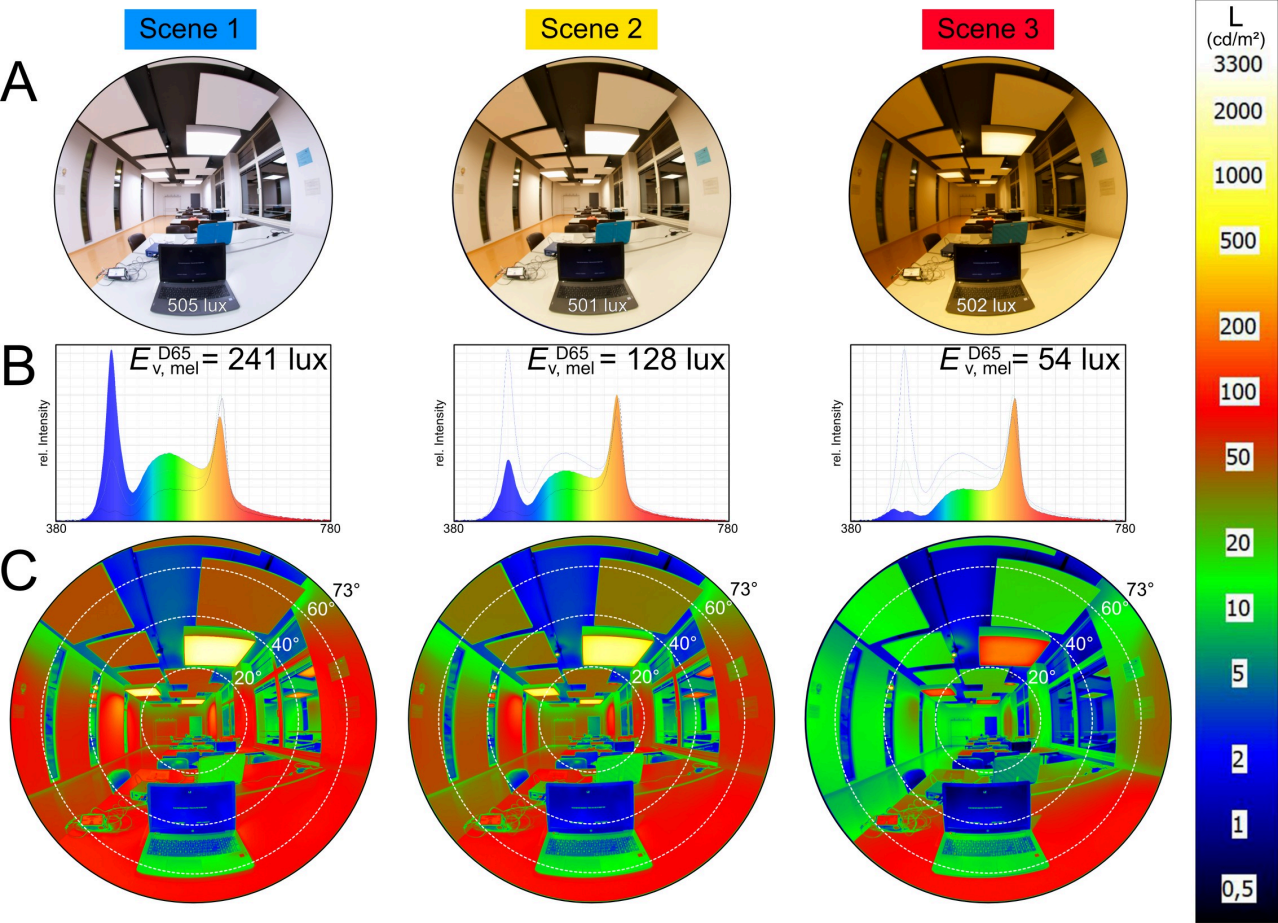
Overview of light scenes. From left to right: Scene 1, 2, and 3. Pictures and measurements are taken from the participant’s viewpoint. Height 120 cm above the floor, with vertical alignment. (A) 150° fisheye photograph, with illuminance measurement value at the desk surface. (B) Spectral relative intensity from a wavelength (λ) of 380 to 780 nm; E^D65^_v,mel_: melanopic equivalent daylight (D65) illuminance according to CIE S 026. (C) False-colour luminance distribution in a 150° fisheye photograph; scale on the right-hand side in cd/m^2^. Dotted lines indicate 20°-viewing-angle increments from the vertical centre point.

Three different lighting scenarios were tested. Lighting settings were chosen to maximize or minimize the effective melanopic stimulus at the eye level (i.e., 120 cm height above the floor, vertical alignment) relative to a medium intensity setting, while keeping the illuminance at the task level at 500 lux (75 cm height above the floor, horizontal alignment). Task-level illuminance was chosen as required by the standards for office workspaces in Europe [30]. Fisheye photographs, together with irradiance distributions from the participants' perspective, are shown in *Fig 1*. Measurements at the eye level and desk surface are presented in *Table 1*. The CIE S 026 standard was used for metrology [13]. While to be expected [31], it is notable that, through the influence of room surface reflections, the spectral distributions at eye-level differ from those of the light radiating from the lighting fixtures. In Scene 1, the melanopic stimulus is maximized, with light mainly coming from areal light sources at the ceiling. The spectral composition of light is dominated by short wavelengths (blue) compared to medium wavelengths (red/green). Correlated colour temperature, CCT, of the lights is at 7000 Kelvin (K). Scene 2 represents the most common workplace lighting scenario in terms of its spectrum (CCT of the lights being at 4000 K) and its horizontal-to-vertical illuminance ratio. In Scene 3, the melanopic stimulus is minimized, with light mainly coming from spotlight sources at the ceiling. The light spectrum contains very little short-wavelength energy compared to medium wavelengths (CCT of the lights at 2700 K). During the study, the lighting scenarios were manipulated by the experimenter via a smartphone application. Glare was not an issue in all three lighting scenarios; i.e., there was no reflection glare on the desk surface or the monitor. Average luminance ratios of the task area to the surrounding area were 2:1, 3:1, and 6:1 (Scene 1, 2, and 3), respectively, indicating no source of discomfort from task-surround luminance differences. The standardized computerized task used a positive contrast on the monitor (luminance ratio 15:1), which led to high luminance ratios to the surrounding task area of up to 100:1. However, this was equal for all experimental conditions, and none of the participants reported having problems reading text on the screen. In conclusion, the melanopic intensity decreased from Scene 1 over Scene 2 to Scene 3, while illuminance at (and luminance coming from) the desk surface remained constant. The raw measurement data for each lighting scenario are provided in S2 Dataset.

**Table 1:** Lighting scenarios–settings and measurement values.

	Scene 1		Scene 2		Scene 3	
**General settings and measurement values**^**1**^						
CCT of the light source (K)	7000		4000		2700	
Horizontal Illuminance at the desk surface (lux)	505		501		502	
**Measurements at eye level**^2^						
CCT at eye level (K)	5912		3539		2489	
Colour-Rendering Index	91		94		91	
Illuminance (lux)	261		209		146	
**α-opic equivalent daylight (D65) illuminance**^3^						
	Full	FOV	Full	FOV	Full	FOV
Melanopsin (MEDI; lux)	**241**	**215**	**128**	**100**	**54**	**31**
S-cone (lux)	246	219	101	78	32	17
M-cone (lux)	251	224	179	140	111	65
L-cone (lux)	261	232	211	165	150	89
Rods (lux)	241	215	141	111	69	40

^1^ CCT of the light source is according to the programmed setting, not a measurement value.

^2^ Measurements were taken at a height of 120 cm above floor level, vertically aligned.

^3^ Calculations are according to CIE S 026 (2018). Measurements are either taken over the full vertical hemisphere (“Full”) or with restrictions of the field of view (“FOV”) according to CIE S 026 Annex 4, Table A (Indoor). Melanopic equivalent daylight (D65) illuminance values are shown in bold.

*Table 1 (α-opic equivalent daylight (D65) illuminance)* and *Fig 1(B)* show that the light-setting changes influence multiple receptor stimuli, not just the melanopsin stimulus. With spectral changes, this is most often the case and can only be avoided by comparing reactions to receptor-specific metameric stimuli, such as Spitschan and Woelders [32] reported. However, the required lighting spectra for melanopsin metamerism are artificially truncated. This would impact the study setup, which uses common lighting conditions, including conditions for good colour rendering. Our use of non-metameric differences in the lighting scenarios, therefore, will not allow inferring the Melanopsin contribution to the effect in question. However, if the change in PEP is mainly mediated by ipRGC sensitivity, as is hypothesized, the experimental change in the light setting should affect the change in PEP according to the ipRGC stimulus.

### Measurements

A Biopac MP36 data acquisition system with a non-invasive cardiac output sensor SS31l and BioPac BSL 4.1 software on a personal computer (PC) were used to record an impedance cardiogram (ICG) and, simultaneously, an electrocardiogram (ECG). Measurement parameters were *impedance* (Z, measured in Ohm), *impedance change over time* (dZ/dt, measured in Ohm/sec), and *ECG Lead II* (measured in mV) with a sampling rate of 2000 Hz. Output was stored in a text file for analysis on the PC. For this device, four disposable strip electrodes (ICG) and two disposable spot electrodes (ECG) were used. The impedance was measured in a four-wire configuration to exclude the effects of skin, electrode, and lead-wire resistance. For this, strip electrodes were attached at the left body side. Two were attached at the base of the neck, with a parallel gap of 3 cm to one another; the other two strip electrodes were placed horizontally and 3 cm below the thoracic xiphisternal joint, with a 3-cm gap to one another. An alternating current was injected via the outer electrodes and the resultant voltage measured from the inner electrodes. For the ECG, one spot electrode was placed above the left ankle, the other one above the right wrist. Jewellery, mobile phones, keys, and other electronics were removed from the body before the measurement. See Sherwood et al. [33] for methodological guidelines. For spectral measurements, we used a *Jeti Specbos 1208* spectrometer; see *Table 1* for results. For luminance measurements, we used a *TechnoTeam calibrated Canon EOS D7* camera with fisheye-lens; see *Fig 1* for results. We further measured ambient room temperature, humidity, and carbon-dioxide level (at a nearby desk) with an *Ahlborn ALMEMO Datalogger* and *Ahlborn D7 sensory equipment*, to control for other criteria that might influence the cardiac reaction. Due to a technical malfunction, these data were partly lost, however, and are only available for 86 of the measurement episodes.

### Procedure

**Fig 2:**
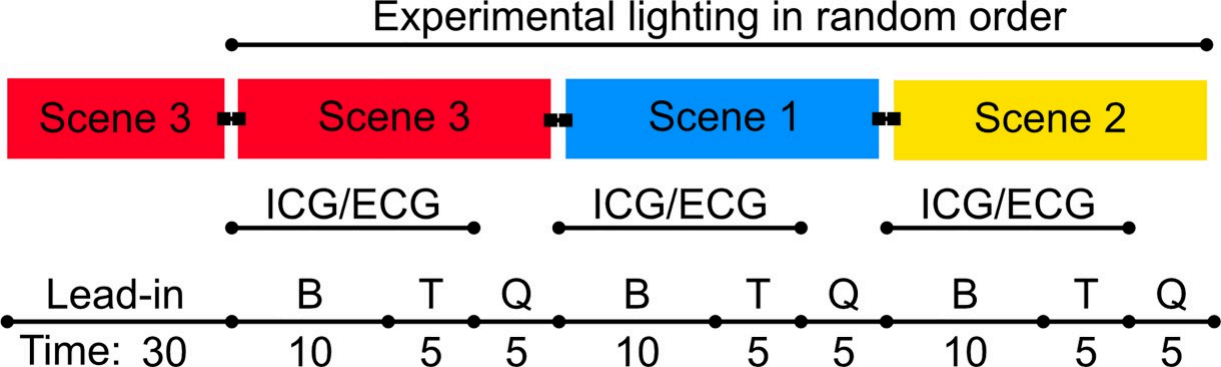
Experimental procedure. Schematic depiction of periods for one experimental session (identical for both morning and afternoon sessions) with exemplary order of lighting scenarios. Light is switched instantly between scenarios. The Unit for *Time* is minutes. B: Baseline period; T: Task period; Q: Questionnaires; ICG/ECG: Cardiac measurement.

The experimental procedure was adapted from Lasauskaite and Cajochen [16]. It was approved by the Ethics Committee of the Munich University of Applied Sciences. Participants arrived at the learning room at the appointed time (either 7:00 am or 5:00 pm) and were welcomed and seated in a wooden chair. Wood was chosen in order to minimize possible electrostatic charge, e.g. from clothing with synthetic fibres. Participant then read and signed the prepared informed consent form and filled out a questionnaire, containing four parts: (1) The German translation of the Morningness-Eveningness Questionnaire for testing chronotype (D-MEQ [28]); (2) the Karolinska-Sleepiness Scale for sleepiness (KSS [34, 35]); (3) general demographic questions; (4) questions regarding participants’ current state of health, and activities on the day, and the night before, the appointed time and day of testing (e.g. bedtime, wake-up time). Absence of sleepiness is commonly used as measure of alertness [36] and will be used as such in the present study. The experimenter then applied the electrodes, started the ICG/ECG measurements, and visually controlled for acceptable signal patterns. Participants then tried one block of the cognitive task described below. Up to this point, lighting was set to Scene 3 to minimize potential blue-light-induced carry-over effects to the first experimental episode. Before the first light scene change and before every subsequent change, room windows were opened briefly for cross ventilation. Participants were informed that lighting would be changed (i.e., both when the scene was changed or left unchanged) and that a "relaxation phase" of ten minutes would follow. Lighting remained the same until the end of the experimental episode (see *Fig 2*). During the baseline phase, participants were provided with current popular magazines for light reading. After the ten-minute baseline, they performed a computerized cognitive task (Sternberg task, PEBL 2.0 [37, 38]), which took about five minutes to complete. The Sternberg task is a letter-memorization task; default software settings were used. The task was divided into six blocks, each containing fifty trials. The blocks varied in the number of letters to be memorized, counterbalanced for two, four, and six letters. Each trial consisted of a single letter, presented on-screen. By pressing one of two buttons, the participant chose whether the presented letter was among the series of letters memorized at the beginning of each block. The participants had feedback on whether their response was correct. When they chose incorrectly, the memorized letter series would be presented briefly (750 ms). After finishing this task, ICG/ECG measurements were ended, and participants filled out questionnaires. These were: (1) the Karolinska Sleepiness Scale (KSS, see above), where participants rated their sleepiness on a scale of 1 (*extremely awake*) to 9 (*extremely sleepy–fighting sleep*). Then followed (2) the NASA Task Load Index Questionnaire (TLX [39]), where participants rated the task difficulty level in categories of *mental demand*, *physical demand*, *temporal demand*, *performance*, *effort*, and *frustration*, on a scale of 0 (low) to 20 (high). (3) Finally, participants also ranked the appeal of the lighting situation on a 5-level ordinal scale (*very good*, *somewhat good*, *neither good nor bad*, *somewhat bad*, *very bad*). After the second or third lighting condition of the experimental session, participants ranked the appeal of the lighting situation in comparison to the previous one *(better*, *similar*, *worse*). After the questionnaires were filled out, the lighting scenario was switched to the second experimental condition and the same tasks performed, and finally the third light scene tested in the same way. Participants then left, coming back at the second appointed day and time. Then, all steps described above were repeated, save for filling out the consent form (the first one already covered the whole experiment) and questionnaires on chronotype and demographic data. At the end of the second experimental session, participants were thanked, debriefed, and received their payment. The total time participants spent for the experiment was about 180 minutes, with about 90 minutes per experimental session.

### Data analysis

The pre-ejection-period (PEP) is the time span between the depolarization of the left ventricle (Q onset) and opening of the aortic valve (B point) [40, 41]. For practical reasons, the R onset (Q peak) is taken as a fiducial indicator of the beginning of the depolarization and can be picked up from the ECG signal [41]. As signature for the beginning of the depolarization, we took the minimum of the ECG’s second derivative. Its peak indicates the maximum curvature at the start of the R onset which, concurrently, represents the peak of the Q wave. Since the electrical dipole during the Q wave is approximately orthogonal to the Lead-II axis, and the electrical axis of the heart varies between the individual subject and its posture, using this electrode configuration may result in an absence, or large between-subject variability, of the Q-wave in the ECG signal [41]. The minimum of the ECG’s second derivative neither depends on the visibility of the Q wave nor the amplitude of its peak, which makes it a reliable indicator for the R-onset. Furthermore, we restricted the possible occurrences of R onset to a time window preceding the, easily identifiable, R peak. Within that window, the R onset is typically seen as a clear negative peak of the ECG signal’s second derivative, which can be located reliably and with high precision, and thus allows a reliable identification of the PEP onset. Heart rate (HR) was then calculated from the time difference of subsequent R peaks.

The time point for the opening of the aortic valve (B point) was derived from the impedance cardiogram (ICG). The impedance Z, and thus the ICG, are sensitive to a variation of blood volume in the thorax [33]. The first derivative, dZ/dt, corresponds to blood flow. The second derivative d^2^Z/dt^2^, in turn, corresponds to a change of the blood flow and is thus indicative for the opening of the heart valves. The B point is the onset of the aortic valve’s opening, indicated by a negative peak in the third derivative, d^3^Z/dt^3^. While, compared to ECG, the ICG signal is smooth and devoid of characteristic spikes, its first, second, and third derivative show distinct features. As selection criterion for picking the correct peak of the third derivative, we used the, easily identifiable, peak of the first derivative, dZ/dt. The B point is obtained as the minimum of d^3^Z/dt^3^ that occurs just before the maximum in dZ/dt. This strategy allows for an automated evaluation of the PEP interval for the large data sets, with few outliers and the required precision.

Numerically, the ICG and ECG signals were processed offline using a *Wolfram Mathematica Notebook* script, written for this project. To calculate the derivatives of the measured signals, we used the *Savitzky-Golay* filter [42], as described in *Numerical Recipes* [43]. This method allows data smoothing, while keeping intact signatures like peaks, and the simultaneous determination of derivatives. Similar to a moving average, a moving section of the data is selected. However, instead of a simple averaging, the algorithm fits a polynomial of given degree to the selected sequence. Then, one point of the fitted polynomial (usually the central point) is taken as value for the smoothed curve. Higher derivatives are taken from the corresponding derivatives of the fitted polynomial at the respective point. The *Savitzky-Golay* filter is implemented numerically by a list convolution with a kernel. That kernel is calculated in advance for the number of points for the moving fitting, the order of the polynomial, and the order of the derivative. The routines for the kernel’s calculation and for list convolution are provided by *Mathematica*, but are available in most numerical environments. We used a kernel length of 100 points, corresponding to a time interval of 50 ms, and a 3rd-order polynomial for all kernels and for the ICG and ECG signals. The third derivative of the ICG signal was calculated from the first derivative of the ICG signal, which, together with Z, was provided by the *Biopac MP36* system. We ensured that no time lag was introduced between the ICG and ECG signals and their derivatives by the *Savitzky-Golay* filter. While the *Savitzky-Golay* filter is conceptually straightforward, other higher-order finite, or infinite, impulse response filters (FIR or IIR filters) would produce similar results with appropriate filter settings. Other examples for a calculation of the B point from Z and its derivatives can be found in the literature [33, 40, 44].

Thus, PEP and LVET data were extracted from the ICG and ECG measurements in a semi-automated way and with a by-heartbeat resolution. The *Mathematica Notebook* output was stored in a text file, with separate files for the baseline and the task period. Each text file contained a timestamp containing the corresponding length of cardiac PEP, LVET, and HR for each heartbeat. The described *Mathematica Notebook* script is part of the *Supporting Information* as *S2 File*. We encourage its use by other researchers.

In the next step, we eliminated artefacts and implausible values (using a one-minute moving average and visual control in Microsoft Excel). We also controlled for possible preload or afterload effects (ventricular filling or arterial pressure, respectively), by comparing changes in PEP to changes in LVET [45, 46]. This was done by calculating the period of the electromechanical systole (EMS) as the sum of PEP and LVET. According to Sherwood and colleagues [33], decreases in PEP should be accompanied by decreases in EMS, i.e. positively correlated, in order to tentatively infer increased beta-adrenergic activation without loading factors. Correlation was visually inspected for each episode. Finally, for every measurement period (baseline or task), the average PEP, LVET, and HR were calculated, together with their standard deviations, and the correlation of each parameter with time was further determined. Cardiac reaction scores were calculated by subtracting the task average from the corresponding baseline average; they are marked with the Greek letter ‘Δ’ preceding the variable (e.g., “ΔPEP”). Negative ΔPEP values indicate shorter periods and thus more forceful cardiac contraction during the task period compared to the baseline. ΔPEP and ΔEMS were positively correlated between periods of rest and task (*r* = +0.39, p<0.001). This, in combination with the visual inspection of the unaggregated data, leads us to believe that no systematic loading effects were introduced to the ΔPEP data, leaving changes in positive inotropic, i.e. adrenergic, agents as the main cause for changes in PEP. A more forceful cardiac contraction, excluding preload and afterload effects, is the result of higher sympathetic activation of the heart [47] and is indicative of a higher experienced task demand and effort [20].

### Statistical methods

We used the *R* software [48] with the *lme4* package [49] to perform a *linear mixed-effects analysis* of the relationship between ΔPEP, and the light scene plus time-of-day. As random effects, we had intercepts for participants. Following Barr et al. [50], we tested random slopes for participants, but models in that case would not converge. We also tried other ways to include random slopes in partial models which, however, led to the same outcome. Random slopes were therefore left out of the final model [50]. Visual inspection of residual plots and random-intercept plots of the used model did not reveal any apparent deviations from homoscedasticity or normality. If not stated otherwise, p-values were obtained by likelihood-ratio tests of the full model with the effect in question, against the model without the effect. P-values less than or equal to 0.05 were considered significant. Beta coefficients (β) from the final mixed-effect model are given where appropriate. They represent the change in value of the dependent variable, when increasing the continuous predictor variable by one unit, or when changing the state of a nominal predictor variable. The continuous predictor variable or, respectively, the nominal state, is indicated by the beta coefficient’s subscript. For *light scene*, Scene 2 is always used as the baseline; with respect to *time of day*, late afternoon is the baseline. Group averages (M) and standard deviations (SD) are given for all dependent variables; those for the cardiac reactivity parameters are displayed in a dedicated table. The proportion of variance accounted for (R^2^), as well as effect size (*f*^*2*^), was calculated according to Selya et al. [51]. Values of *f*^*2*^ ≥ 0.02 are considered small effect sizes, *f*^*2*^ ≥ 0.15 medium, and *f*^*2*^ ≥ 0.35 large effects [51]. 95% confidence intervals for beta coefficients are denoted with *CI*, and units are left out for clarity. Confidence intervals were calculated as *profile confidence intervals* using the *lme4* package. The described methods and principles were also used for exploring other dependencies of light, time of day, and several other variables on cardiovascular baselines, cardiovascular reactivity of HR and LVET, task ratings, and task performance, even though we did not formulate *a-priori* hypotheses for these cases.

We used *Lilliefors test for normality* [52] on cardiac parameter values, to test the normality assumption prior to the *linear mixed-effect analysis*. All values are normally distributed at a 0.05 level of significance.

The appeal of the current lighting situation, in general as well as compared to the prior light scene, was analysed using *cumulative link mixed models* with the ordinal package in *R* [53].

Plots in the *Results* section were made with the R software. Raincloud plots were made according to Allen et al. [54]. The R code to create the plots from the data in this study is provided as part of the *Supporting Information*, in the file *S1 File*.

## Results

### Preliminary analysis

In a preliminary analysis, we looked at time effects within the different experimental periods, sleepiness scores at the beginning of each session, and room-level measurements. We tested for time effects during baseline and task periods, i.e. tested whether the values for PEP, HR, or LVET changed with the duration of the respective period, using the correlation with time (r_t_) as the dependent variable in a linear regression model. During the baseline period, PEP was not dependent on time (p = 0.40), and neither was HR (p = 0.21), but LVET declined slightly with duration (r_t_ = -0.05, p = 0.01). During the task period, PEP was not dependent on time (p = 0.27), but HR increased (r_t_ = 0.16, p<0.001) and LVET declined (r_t_ = -0.11, p<0.001). Neither the light scene nor the time of day were predictive of r_t_ values of PEP, HR, or LVET (all p≥0.12).

Further, sleepiness (KSS score) was tested for the beginning of each time-of-day session (KSS_D_). KSS_D_ scores (M_KSS_D_ = 4.39, SD_KSS_D_ = 1.83) showed no dependency on time-of-day, sex, or chronotype (all p>0.35), but were dependent on sleep duration in the night before the experiment (p = 0.02), with decreasing sleepiness for increasing sleep duration (β_sleep_ = -0.20, CI: -0.37 to -0.03).

Finally, we tested whether room levels of carbon dioxide (CO_2_), temperature (T), or relative humidity (H) differed between experimental scenarios and whether they were predictors of ΔPEP. None of those variables showed dependency on the light scenario (all p>0.30). CO_2_ levels (M_CO2_ = 900 ppm, SD_CO2_ = 14 ppm) were dependent on time-of-day (p = 0.02), with slightly lower values in the morning session, compared to late afternoon (β_Morning_ = -6 ppm, CI: -11 to -1). Temperature levels (M_T_ = 22.35°C, SD_T_ = 1.54°C) were dependent on time-of-day (p<0.001) as well, with slightly lower values during the morning session, compared to late afternoon (β_Morning_ = -0.96°C, CI: -1.42 to -0.50). Humidity levels (M_H_ = 25.3%, SD_H_ = 6.4%) did not vary between time of day (p = 0.59).

### Cardiac reactivity

Cardiac reaction scores, calculated as differences from baseline to task values, are presented in *Table 2*.

**Table 2:** Cardiac reactivity scores.

	Scene 1	Scene 2	Scene 3
Morning session			
ΔPEP	-0.05 (±0.77)	-2.02 (±1.08)	-0.63 (±0.75)
ΔHR	0.27 (±0.71)	1.00 (±0.65)	0.39 (±0.60)
ΔLVET	3.20 (±2.01)	-1.93 (±2.23)	1.70 (±1.80)
Late-afternoon session			
ΔPEP	-1.72 (±0.78)	-3.88 (±0.99)	-2.27 (±0.98)
ΔHR	1.53 (±0.50)	2.01 (±0.81)	1.96 (±0.73)
ΔLVET	1.57 (±2.15)	2.84 (±1.42)	-0.22 (±1.93)

Means and standard errors (in parentheses) for changes in (1) the pre-ejection period (ΔPEP), (2) heart rate (ΔHR), and (3) left ventricular ejection time (ΔLVET), from baseline to task periods. Measurement units are milliseconds (ms) for ΔPEP and ΔLVET, and beats per minute (bpm) for ΔHR. N = 26 for late-afternoon sessions of Scene 1 and Scene 2, n = 27 for all other sessions.

#### Cardiac PEP reactivity: Contraction

**Fig 3:**
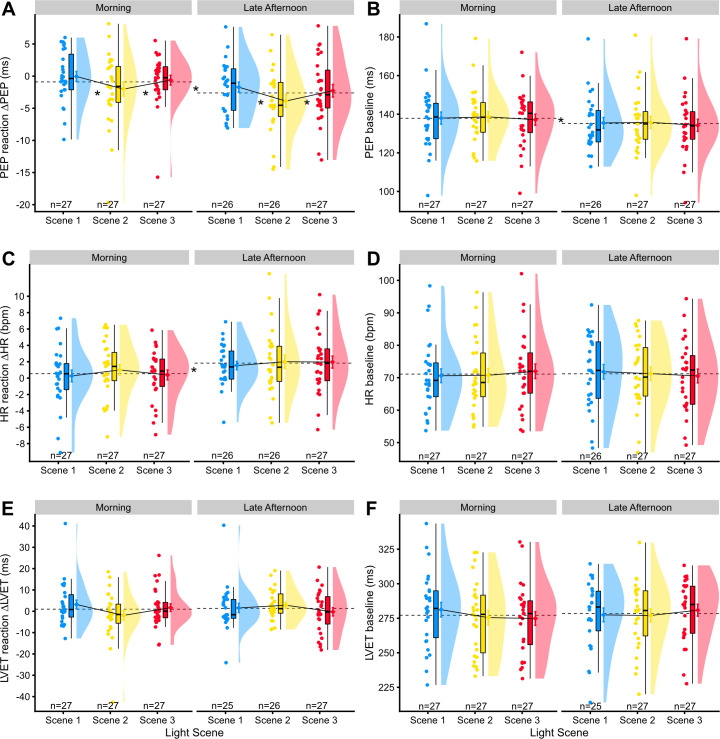
Raincloud plots for cardiac reaction scores and baseline scores. **(**A), (C), and (E) show cardiac reaction scores, and (B), (D), and (F) cardiac baseline scores, each differentiating between the factors of light scene and time of day. There are three Raincloud plots at each time of day (blue, yellow, and red); each of these consists, from left to right, of (a) the raw data, horizontally jittered to improve readability, (b) a Boxplot, and (c) a density plot of the data, with a dot with whiskers on its left, indicating its mean and standard error of the mean. Continuous sloped black lines between the means track the differences between light scenes. Horizontal dashed lines indicate the mean over all three light scenes during the respective morning or late-afternoon session. Asterisks indicate a significant change between the respective conditions, marked by the continuous or dashed lines. The number *n* at the bottom of each plot indicates the sample size for the respective Raincloud plot. Abbreviations: PEP: cardiac pre-ejection period; ΔPEP: change in PEP; HR: heart rate; ΔHR: change in HR; LVET: cardiac left ventricular ejection time; ΔLVET: change in LVET; bpm: beats per minute; ms: milliseconds.

With respect to PEP reactivity, results for ΔPEP are shown in *Fig 3*. Increased cardiac contraction, i.e., a decreased systolic time interval for the pre-ejection period (ΔPEP), was dependent on the light scene (p = 0.01). A ΔPEP value of zero indicates no change in PEP from baseline to the task. Relative to Scene 2, Scenes 1 and 3 both led to a reduced cardiac contraction (β_Scene1_ = +1.97 ms, CI: 0.60 to 3.33; β_Scene3_ = +1.50 ms, CI: 0.14 to 2.85). Overall effect size for *light scene* (*f*^*2*^_*light-scene*_) was 0.07.

Besides being dependent on the lighting scenario, ΔPEP also depended on the time of day (p = 0.002). During morning sessions, the change in cardiac contraction was reduced compared to late afternoon (β_Morning_ = +1.78 ms, CI: 0.67 to 2.89). Effect size (*f*^*2*^_*time-of-day*_) was 0.08. The interaction of light scene and time of day on ΔPEP was tested but had no relevant predictive power (p = 0.99). Sex, age, BMI, sleep duration, chronotype, and KSS_D_ were no relevant predictors (all p≥0.65).

The mixed-effect model with light-scene and time-of-day predictors had an effect size *f*^*2*^_*total*_ of 1.05 and R^2^_total_ of 0.51, but this includes the random effect for participants and therefore mostly accounts for the inter-individual variance (SD_Participants_ = 2.89 ms), not the experimental conditions. When using a random-effect-only model as a reference, the combined effect size for the light scene and time of day was *f*^*2*^ = 0.15, and the explained variance R^2^ = 0.13 (SD_Residual_ = 3.55 ms).

**Fig 4:**
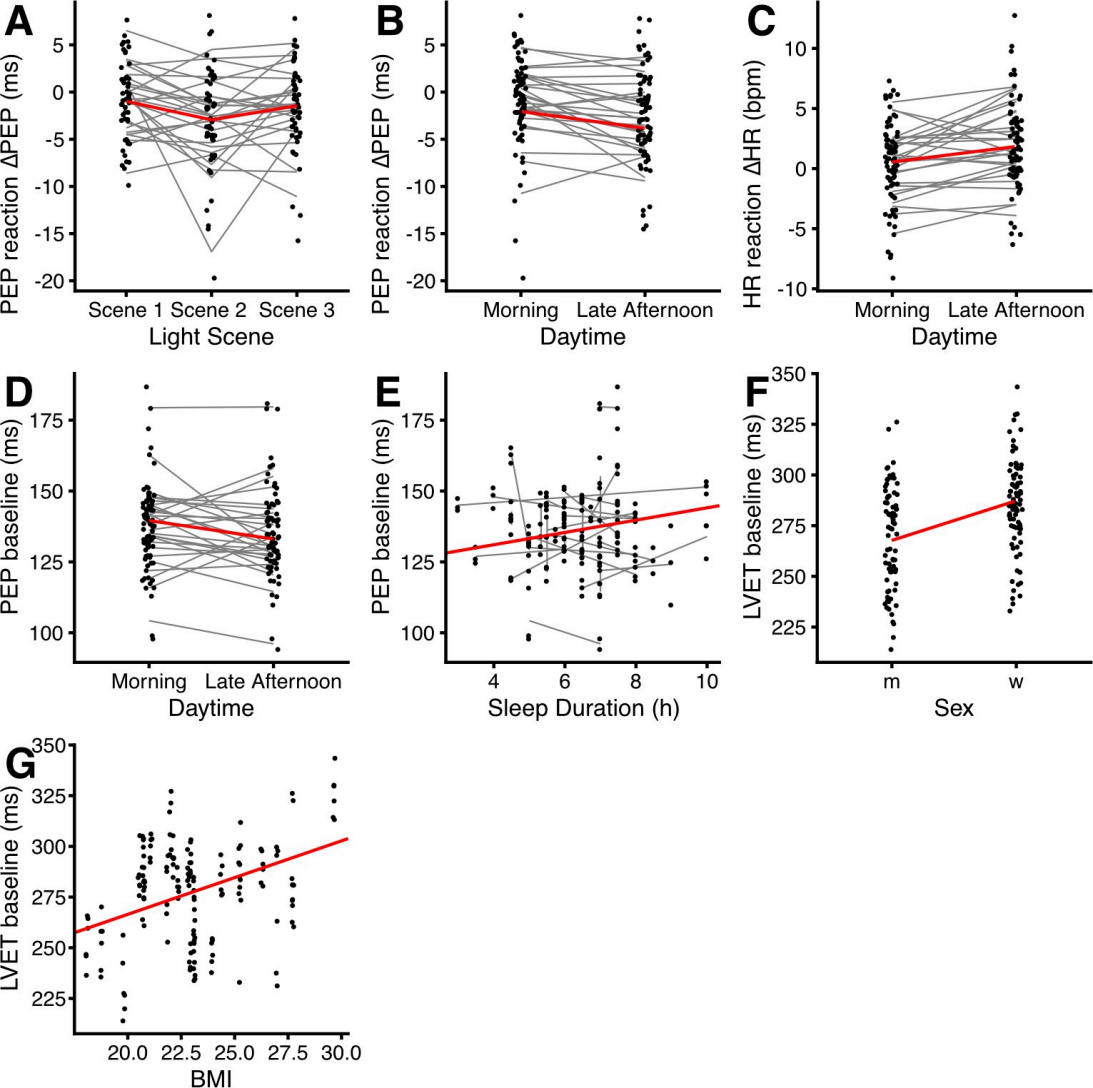
Scatterplots of cardiovascular dependencies on a number of predictors. Points show individual values; grey lines indicate the intraindividual change between predictor levels. Each of these lines connects the predicted means of up to three individual values. Regression lines when all other predictors are held constant on an average level are shown in red. For the categorical predictors (A–D, and F), points are jittered horizontally to improve readability. (A) ΔPEP values are lowest for Scene 2. (B) ΔPEP values are higher in the morning, compared to late afternoon. (C) ΔHR is lower in the morning, compared to late afternoon. (D) PEP baseline values are higher in the morning, compared to late afternoon. (E) PEP baseline values increase slightly with sleep duration. Sleep duration refers to the sleep the night before the respective experimental session. (F) LVET baseline values are higher for females (w) than for males (m). (G) LVET baseline values increase with BMI values. Abbreviations: PEP: cardiac pre-ejection period; ΔPEP: change in PEP; HR: heart rate; LVET: cardiac left ventricular ejection time; BMI: body mass index; bpm: beats per minute; ms: milliseconds.

Finally, we also tested for influential data points on a by-participant level, i.e., we dropped individuals from the analysis to test whether the reported effects depended on those participants. For the predictors *light scene* and *time of day*, there was no influential participant. However, the CI for β_Scene3_ crossed the zero mark four times (i.e. was not part of the 95% CI) out of twenty-seven participants. This means that there is some sensitivity to the sample for Scene 3 being significantly different from Scene 2 in the effect on ΔPEP values (see Fig 4(A) for the individual trends between light scenes). The empirical trend, i.e. the difference of ΔPEP between Scene 2 and Scene 3, stayed broadly the same, regardless of the excluded participant.

#### HR and LVT reactivity

With respect to the other cardiac reactivity variables, we tested ΔHR and ΔLVET for dependencies on the light scene, time of day, the interaction between light and daytime, sex, age, BMI, sleep duration, chronotype, and KSS_D_. Time of day was predictive of ΔHR (p = 0.001), with a smaller increase in heart rate during the morning session compared to late afternoon (β_Morning_ = -1.29 bpm, CI: -2.07 to -0.52; see Figs 3 and 4). Other parameters had no significant effect on ΔHR (all p≥0.08). None of the independent variables was predictive of ΔLVET (all p≥0.06).

### Cardiovascular baselines

Cardiovascular baseline parameters in the study were PEP, HR, and LVET. We tested these values during the baseline period for dependencies on the light scene, time of day, interaction between light and daytime, sex, age, BMI, sleep duration, chronotype, and KSS_D_. Fig 4 shows scatterplots of all cardiovascular dependencies.

PEP values (M_PEP_ = 136.54 ms, SD_PEP_ = 15.10 ms) were dependent on time of day (p<0.001) and on sleep duration (p = 0.001), with higher values obtained during the morning session compared to late afternoon, and increasing values with longer sleep duration (β_Morning_ = +6.51 ms, CI: 3.43 to 9.58; β_Sleep_ = +2.17 ms, CI: 0.85 to 3.49). Other predictive parameters on PEP were not significant (all p≥0.09).

Surprisingly, HR baseline values (M_HR_ = 71.17 bpm, SD_HR_ = 10.89 bpm) were not dependent on the time of day, nor on any of the other parameters (all p≥0.08).

LVET values (M_LVET_ = 277.80 ms, SD_LVET_ = 24.96 ms) were dependent on sex (p = 0.003) and BMI (p = 0.002). Values for LVET were greater for females, compared to males. LVET values further increased with higher BMI (β_Female_ = +19.53 ms, CI: 6.28 to 32.78; β_BMI_ = +3.62 ms, CI: 1.24 to 6.01). Other predictive parameters on LVET were not significant (all p≥0.22).

### Task performance and retrospective self-report

#### Task performance

Task performance parameters were reaction time and accuracy. Both were tested for dependencies on the light scene, time of day, the interaction between light and daytime, sex, age, BMI, sleep duration, ΔPEP, chronotype, and KSS_D_. Accuracy was logit transformed for linearization [52]. Besides calculating average performance values, the PEPL 2.0 software output includes performance for two, four, and six character blocks of the cognitive task. The block sizes can be likened to task difficulties.

Reaction time (grand M_RT_ = 851 ms, SD_RT_ = 178 ms) showed a dependency on sleep duration, but only for the four-character block (p = 0.02), with increased reaction time with sleep duration (β_Sleep_ = +11.70 ms, CI: 1.90 to 21.50). Other predictive parameters on reaction time were not significant, regardless of character count (all p≥0.10).

Accuracy (grand M_Acc_ = 97.7%, SD_Acc_ = 0.02%) was dependent on KSS_D_, when testing for the six-character block or the average over all the blocks (p = 0.002 and p = 0.003, respectively). Accuracy decreased with increasing sleepiness score (six characters: β_KSSD_ = -0.25, CI: -0.41 to -0.08; average: β_KSSD_ = -0.19, CI: -0.31 to -0.07; β values and CI relate to logit transformed accuracy values). Other predictive parameters on accuracy were not significant, regardless of character count (all p≥0.06).

#### Task ratings

Questionnaire-derived ratings for task demand consisted of all six items from the NASA Task-load-index. Scores for the items *mental demand*, *temporal demand*, *performance*, *effort* and *frustration* were summed up to calculate a Raw-TLX score (RTLX [55]). RTLX scores were tested for dependencies on the light scene, time of day, sex, KSS_D_, sleep duration, and chronotype. Raw-TLX scores (M_RTLX_ = 50.6, SD_RTLX_ = 11.7) were dependent on time of day, sex, and sleep duration (p = 0.017, 0.006, and 0.005, respectively). RTLX scores were higher for females, compared to males, and lower during morning sessions, compared to late afternoon. RTLX scores were further lower for increased sleep duration (β_Female_ = +10, CI: 2.6 to 17.4; β_Morning_ = -1.4, CI: -2.3 to -0.4; β_Sleep_ = -1.4, CI: -2.3 to -0.4). Other predictive parameters for RTLX were not significant (all p≥0.30).

Single-item-TLX scores showed those dependencies in parts. *Mental demand*, *physical demand*, *effort*, and *frustration* had different scores depending on sex (all p≤0.04). *Temporal demand* and *frustration* had different scores depending on the time of day (all p≤0.02). *Frustration* depended on sleep duration (p = 0.008). Finally, *physical demand* and *performance* depended on KSS_D_ (all p≤0.04).

#### Other ratings

Other questionnaire-derived ratings contained sleepiness after each task period and the appeal of the lighting situation. KSS scores after each task period (KSS_S_) were tested for dependencies on the light scene, time of day, sex, sleep duration, KSS_D_ (scores the sleepiness at the beginning of the session), chronotype, and ΔPEP. KSS_S_ scores (M_KSS_S_ = 4.66, SD_KSS_S_ = 1.92) were dependent on KSS_D_ (p<0.001). Unsurprisingly, KSS_S_ scores increased with higher KSS_D_ scores (β_KSSD_ = +0.76, CI: 0.63 to 0.89). Other predictive parameters for KSS_S_ were not significant (all p≥0.06).

The appeal of the current lighting situation in general, as well as the appeal compared to the prior light scene, were tested for dependencies on the light scene, time of day, the interaction of light scene with the time of day, chronotype, sex, KSS_D_, and prior light scene. The appeal of the current lighting situation, scored on a Likert scale from one to five, was dependent on the light scene (p<0.001), but not on other parameters (all p≥0.29). No participant rated the current lighting situation as *very bad* (lowest of five scores). It was more likely that participants rated Scene 1 lower than Scene 2. Scene 3 was rated about as appealing as Scene 2 (β_Scene1_ = -1.94, CI: -2.93 to -0.94; β_Scene3_ = -0.19, CI: -1.14 to +0.77; β values and CI relate to the *cumulative-link mixed-model* output).

The appeal of the current lighting situation compared to the prior situation, scored on a three-step ordinal scale, was dependent on the light scene (p = 0.001), but not on other parameters (all p≥0.06). It was more likely that participants rated Scene 1 lower than Scene 3 (β_Scene1_ = -1.05, CI: -2.11 to +0.01; β_Scene3_ = +1.03, CI: -0.21 to +2.26; β values and CI relate to the *cumulative-link mixed-model* output).

## Discussion

In light of the results of Lasauskaite and Cajochen [16] and the assumed underlying mechanisms, we anticipated that an increase in melanopic stimulus intensity (increasing from Scene 3 over 2 to 1) would result in a decrease of cardiac contraction change, as measured by ΔPEP. However, while ΔPEP did indeed depend on the lighting conditions, the results show that the melanopic stimulus is not the singular, linear predictor that we expected. Rather, ΔPEP had a minimum at the medium-intense melanopic stimulus during Scene 2, and higher values for Scene 1 (~2.0 ms) and Scene 3 (~1.5 ms). A second-order predictor for the melanopic stimulus intensity would fit the experimental data, but there is no support for such a stimulus-effect connection in literature. Rather, a linear dependency would be expected when looking at other melanopic effects in a similar range of melanopic stimulus intensities [4, 14]. Since all photoreceptor types are influenced by the light-setting changes, they could be possible mediators for the effect on ΔPEP. However, no single receptor stimulus can be used to satisfactorily explain the results. We thus find it likely that a combination of receptor inputs mediate the effect. Therefore, another possibility for an interpretation is using both the melanopic stimulus intensity (E_V,mel_^D65^) and the level of vertical illuminance at the eye as predictors (p = 0.003 and p = 0.005, respectively; β_Ev,mel,D65_ = +0.06 ms, CI: 0.02 to 0.10; β_Ev_ = -0.10 ms, CI: -0.16 to -0.03). From a theoretical viewpoint, we favour that latter possibility even though our data do not provide direct evidence for this moderator effect of illuminance. In the literature, however, brightness has been shown to have alerting effects, independent of melanopic efficacy [56, 57], while downstream effects of a melanopic stimulus can counter sympathetic effects, such as pupil re-dilation [58]. From a physiological perspective, the intrinsic melanopic reaction could be moderated by an extrinsic cone input, as is the case with other NIF effects, such as pupil reaction [59, 60]. Extrinsic rod input is unlikely to occur, since even the lowest light setting is well above the saturation level of rods. Therefore, greater sympathetic activation (i.e. cardiac contraction) with increasing brightness and decreasing melanopic stimulus intensity could result in the present experimental outcome for a light-scene dependent ΔPEP. Further research would be needed, however, to support such an interpretation, especially considering the changes in multiple photoreceptor stimuli, as was discussed above and in *Materials and Methods*.

The second hypothesis - that there is a dependence of ΔPEP on time of day and interaction with the light scene - was only partly supported by the experimental outcome. During morning sessions, changes in cardiac contraction were lower by about 1.8 ms when compared to late-afternoon sessions, which is about the same amount as for a change in light scene from Scene 1, or 3, to 2. To the best of our knowledge, this time-of-day dependent, task-triggered change in cardiac contraction has not been reported before. It is unlike the exploratory findings from van Eekelen et al. [61], even though circadian effects on PEP itself are well known [62, 63] and nearly all physiological processes appear to depend on circadian phase [64]. Our data did not support the hypothesized interaction of the light scene with time of day, but that might still occur for other scenarios, such as at late-night times. One limitation of the present study is a chronotype bias, where most of the participants were neutral types, some morning types, and only two were pronounced evening types. This skew in the chronotype distribution is possibly due to the early start for the morning session, which might have put off some prospective evening-type participants. Therefore, we do not know whether the changes due to time of day would be different for evening chronotype subjects, as is suggested in a study by Goldstein et al. [65].

Effect sizes for the light scene and time of day are small (*f*^*2*^_*time-of-day*_ = 0.08 and *f*^*2*^_*light-scene*_ = 0.07) to medium (*f*^*2*^_combined_ = 0.15), and this is to be expected. Firstly, using a repeated-measures design for the cognitive task usually, through learning effects, dampens the impact on the autonomous nervous system–even when this effect is excluded through randomization, as was done in the present study. Through repetition and learning, the task demand for the participant is expected to decrease, thereby reducing mobilized effort and sympathetic activation. However, this learning effect might more accurately reflect real-life physiological behaviour, where cognitive tasks are usually executed more than once. Secondly, from a theoretical perspective, cardiac contraction reactivity allows to adjust for widely different requirements and shows vast inter-individual differences [45]. Within reasonable limits, changes in lighting conditions and time of day have to be considered circumstantial to the task itself–we would not expect them to change the perception of a given task difficulty entirely. Instead, the effects of lighting and time of day seem to work rather subtly by adjusting the ergonomics and, therefore, perceived demand of a task, and their respective effect sizes do neither overstate their importance nor render them irrelevant. The proportion of possible change in resting PEP for our data is about ten percent, relative to an artificially induced maximum of about 40 ms [47].

Since we did not have an *a-priori* hypothesis for other parameters, the results for those will not be discussed in detail but only for providing context for the primary experimental outcome. The analysis of secondary parameters should also be viewed as exploratory, due to the accumulation of type I errors through the multitude of comparisons. As already stated above, physiological (and ultimately behavioural) parameters are expected to change with the time of day. It is therefore not surprising to see such dependencies for a range of cardiovascular parameters and task ratings. Peculiarly, heart rate itself was not dependent on time of day, but this might be because the morning and late-afternoon sessions show similar heart-rate levels in their respective points on the circadian curve [66].

Room-level data were limited, but they still show that there were systematic differences between morning and late-afternoon sessions. Room-temperature and carbon-dioxide levels have the potential to influence performance [67, 68], and therefore, likely, also the experienced task demand and the cardiac reaction. However, changes in carbon dioxide in the room were negligible in size, and a difference of about one degree Celsius between morning and late afternoon is not considered influential at about 22°C [68]. Temperature differences resulted mainly through the building’s heating cycles during winter.

The lighting situation affected the physiological level, but we found no difference in task performance, neither with respect to reaction time nor accuracy. One possible interpretation is that, even though the experimental settings did not change performance, that was because participants were just more or less "efficient" in terms of energy usage and conservation, thereby achieving the same results. Alternatively, experimental settings did change performance, but on a level not detectable with the posed task, i.e., because statistical power was too low to detect the change. On average, performance accuracy was rather high, even for the six-character block (~96%). The lack of change might thus reflect a ceiling effect and future studies would need a higher difficulty level. Retrospective self-reports of psychophysical effects for task demand and alertness did not depend on the lighting scenario either, which might be interpreted in a similar way.

In comparison to Lasauskaite and Cajochen [16], we find that the minimum ΔPEP in both studies occurs at a medium setting (Scene 2 here, and the 4000K scene in that study), and is close to zero for the highest setting (Scene 1 and 6500K scene, respectively). While we find a significant difference from medium setting toward the lowest setting (Scene 3), results from Lasauskaite and Cajochen [16] in that respect are not significant, but tend in the same direction (2800K scene). It is important to note, that in terms of melanopic stimulus alone, the range of our study is between 54 and 241 lux MEDI, whereas in the other study the range is between 301 and 563 lux MEDI. This means that the lowest melanopic intensity from Lasauskaite and Cajochen is higher than the highest melanopic intensity in our study. Only in terms of light spectrum / spectral composition are the settings comparable between lowest, medium, and highest setting. This difference in the parameter settings adds weight to the discussion above, that the melanopic stimulus alone cannot predict the cardiac reaction, and that a different or at least additional mechanism is needed to explain the effect. Overall, the difference in ΔPEP from the light scene is smaller in our study (~2 ms between Scene 2 and 1, compared to ~3.5 ms between the 4000K and 6500K scene there). The lower effect might be explained by the overall lower, more moderate stimulus levels used here (e.g., ~241 lux MEDI for Scene 1, compared to ~563 lux MEDI in Lasauskaite & Cajochen’s 6500K scene), or on the repeated-measures setup as explained above. The same applies when comparing effect sizes between the present study (small, *f*^*2*^_*light-scene*_ = 0.07) to the other study (medium, d = 0.51, p = 0.034). The latter found a significant effect of light scenes on ΔPEP for baseline periods of exposure, compared to a habituation phase with 2800K. This was possible, because they used a three-step experimental procedure, as explained above. As we did not have a habituation phase prior to every baseline period, we could only test for differences in baseline PEP itself, but find no statistical dependency on the lighting scenario. As a further comparison, we calculated the Bayes Factor (BF) in that study from the p-value of the main effect of light (p = 0.034; BF = 0.31), and compared it with the Bayes Factor for only the *light-scene* variable in the present study (based on likelihood ratios; BF = 0.014) [69]. BF values represent the odds of the null hypothesis relative to the alternative hypothesis; lower values represent better evidence against the null hypothesis. The Bayes Factor for the present study is, by a factor of about 22, lower than in Lasauskaite & Cajochen’s study (i.e., 0.31/0.014). Therefore, the present study adds firm evidence in favour of a real effect of lighting on ΔPEP, even though not stemming from the melanopic stimulus alone, as was initially hypothesized.

Looking at other publications on effort-related cardiac reactivity, we find similar differences in magnitude and direction of ΔPEP as found in the present study between Scene 2 and Scene 1 (~-2 ms). For example, the difference is similar when lowering the task difficulty (to *Low* instead of *Moderate* [70]), or when paying a lower reward (1 Swiss Franc instead of 15 [71]), or also when putting subjects in a bad mood (*negative mood* instead of *positive mood* [72]). As stated by Wright and Kirby [20], cardiac reaction will decrease when lowering task difficulty or reducing personal involvement. Nevertheless, only in connection with the task outcome, i.e. the performance result, can it be determined whether this is desirable or not. In the case of our present study, Scene 1 and 3 are preferable for the posed task compared to Scene 2: even though effort, and therefore energy consumption (sympathetic activity), was lower, task performance and self-reported task demand were the same.

Sikka et al. [73] showed that blood vessels express melanopsin and display vasorelaxation under blue light, and in a recent study, Stern et al. [74] measured a decrease of systolic blood pressure minutes after a full-body blue-light shower, with no changes in a control group. While the evidence for this effect is still limited, it is worth discussing it in the context of the present study. We cannot rule out the occurrence, or interaction, of blood-vessel mediated melanopic effects with retina-mediated melanopic effects, since the participants’ skin was partly exposed to the room lighting, but we find it rather unlikely. Firstly, when compared to Stern et al. [74], the exposed skin areas of our participants were small, with only face and hands exposed by all participants, plus neck and arms by some. Secondly, irradiance levels were only one thousandth of those in that study. Thirdly, such an effect should have shown up in baseline PEP values, in which we had no relevant differences between lighting conditions nor a dependency on time. Lastly, since we were mainly interested in changes of PEP between two phases with the same lighting conditions, any general effect on blood pressure would influence both phases. We believe, therefore, that any blood-vessel mediated melanopic effect had no appreciable impact on our results for ΔPEP.

A number of studies found alerting effects of blue-wavelength-dominated light that we cannot confirm [7, 75–78]. The different outcome in those studies compared to the present study might be explained by longer light-exposure periods, different light stimulus intensity or incident angle, night-time testing, or by using monochromatic light. Studies with a more common context in terms of stimulus and time of day share similar findings to the present study. Laszewska et al. [79], for example, experimented during regular work hours (noon) with results showing little to no acute impact of the light spectrum on alertness. Finally, as mentioned above, Prayag et al. [21] showed acute physiological activation depending on the melanopic stimulus to some degree but without differences in actual performance. Lasauskaite and Cajochen predicted, that the change in cardiac reaction would be due to a higher alertness at higher colour temperature settings (their model, in brief: higher CCT [Light Scene] → higher alertness [KSS] → higher readiness to perform → less perceived task demand [TLX] → less mobilized effort → less sympathetic activation → less cardiac contraction change [ΔPEP]). The authors could not test this in full, however, since alertness scores (measured as KSS) from the beginning of each session were lost due to technical failure. As stated above, we found no impact on alertness of the light scenes or of the time of day. Perceived task demand, operationalized through the NASA TLX score, neither depended on the light setting in the present nor in the other study. The lack of changes in either alertness or perceived task demand do not necessarily speak against the model from the other study. Since the singular effects of the light scene and time of day on ΔPEP are small, the KSS and TLX scores might not be reliable enough to detect an equally small, underlying change in alertness and perceived task demand, respectively, especially since both were queried in retrospect after the task.

## Conclusion

We studied the question of whether changes in the melanopic stimulus, and the time of day would both influence the change in cardiac pre-ejection period (ΔPEP) when performing a cognitive task. ΔPEP is considered a marker for sympathetic activation and, in turn, for mobilized effort and experienced task demand. Light scenes in our study were all constructed as “common indoor office lighting situations” in terms of their light spectrum, their intensity, and the geometric formation of the light sources. Changes in the melanopic stimulus are necessarily accompanied by changes in the activation of other photoreceptor types, when common white light conditions with good colour rendering are maintained. Therefore, a direct test of a pure melanopsin contribution cannot be achieved. However, differences in the light settings were designed to maximize or minimize the melanopic stimulus, starting from a baseline for all affected dimensions, while keeping other variations small. Under these conditions, the medium melanopic-stimulus setting resulted in a greater sympathetic activation compared to the lower- and higher-intensity setting. This result broadly confirms the connection between the spectral composition of light and cardiac contraction as found by Lasauskaite and Cajochen [16], yet with much stronger evidence as shown by the respective Bayes Factors for the overall effect of light in the two studies. Furthermore, it shows, for the first time, that the lighting setup is relevant in terms of ΔPEP under common lighting conditions. However, the result also shows that melanopic stimulus alone cannot account for the changes in sympathetic activation, as we had assumed. Another predictor is required to explain the peak in sympathetic activation at a medium-intense melanopic stimulus. We propose this predictor to be brightness, on theoretical grounds.

We demonstrated, for the first time, a connection between the effort-related change in ΔPEP and time of day, with smaller sympathetic activation during the morning-session compared to that at late afternoon. Yet, importantly, the physiological changes were not accompanied by changes in performance, nor self-reported task demand or alertness. In the context of this study, Scenes 1 and 3 can be likened to an ergonomic optimization. Compared to Scene 2, the changed lighting condition will thus not change the way tasks are completed. The optimal lighting will rather minimize the required amount of sympathetic activation, i.e. the energy, to perform those cognitive tasks. According to literature, the effect is possibly mediated through heightened alertness and readiness to perform, resulting in a reduction in perceived task demand and mobilized effort.

Our findings have practical implications since they show that the way lighting systems are set-up to satisfy regulatory standards does matter on a physiological, effort-related level, even when acute effects do not manifest themselves in immediate performance increases or reduced task demand. We designed the lighting scenes following recommendations for dynamic, circadian lighting systems, with Scene 1 representing a morning setting, Scene 3 an evening setting [80, 81] and Scene 2 resembling a common approach to meet regulatory standards. It is likely that the acute effects found in the present study add to the positive, mid-to-long-term circadian effects of dynamic lighting.

## Supporting information

S1 Dataset Comma-Separated-Value (CSV) data table used for all linear regression, linear mixed-effect, and cumulative link mixed-model analysis. (CSV)

S2 Dataset A CSV file containing the spectral measurements for all three lighting scenarios, with, and without the Field-Of-View (FOV) occlusion. (CSV)

S1 File A ZIP file containing the R code used for every variable (*.R file extensions). The code can be executed with the free R software (GNU General Public License). The plots, included in the *Results* section, can be created directly from the study data with the file *PEP_Plots*.*R*. For the *Raincloud plots*, additional source files are needed from Allen et al. [54]. Further, the ZIP file contains text files with the R software console output, showing the executed code and the results (*.txt file extensions). Lastly, S1 File contains PDF files for all dependent variables with significant predictor variables. The PDF files contain two plots each, showing the QQ-Plots for *Random Intercepts* and *Residuals* from the linear mixed-effect model.
(ZIP)

S2 File A ZIP file containing The *Wolfram Mathematica Notebook* script for processing electrocardiogram (ECG) and impedance cardiogram (ICG) data into Heart Rate (HR), cardiac Pre-Ejection Period (PEP) and cardiac Left Ventricular Ejection Time (LVET), as discussed in the section on *data analysis*. The ZIP file also contains a PDF file with a printout of the executed script for reference. The script was written for the present study, but we encourage its use by other researchers. Test data is provided as *S3 File*. The script is annotated on a-step-by-step basis, and can be executed with *Wolfram Mathematica*. In addition to the PDF file provided as part of S2 File, the script can be viewed with the free *Wolfram Player*.
(ZIP)

S3 File A ZIP file containing two text files with sample ECG and ICG data. One file contains a large dataset, the other a smaller subset. The files are intended as example data for the *Wolfram Mathematica Notebook* script in *S2 File*.
(ZIP)
