# Supplementary figures and images for: Influence of common lighting conditions and time-of-day on the effort-related cardiac response
Source: PLoS One. 2020 Oct 7;15(10):e0239553. doi: 10.1371/journal.pone.0239553 (PMC7540875; doi:10.1371/journal.pone.0239553)

**Random intercepts**

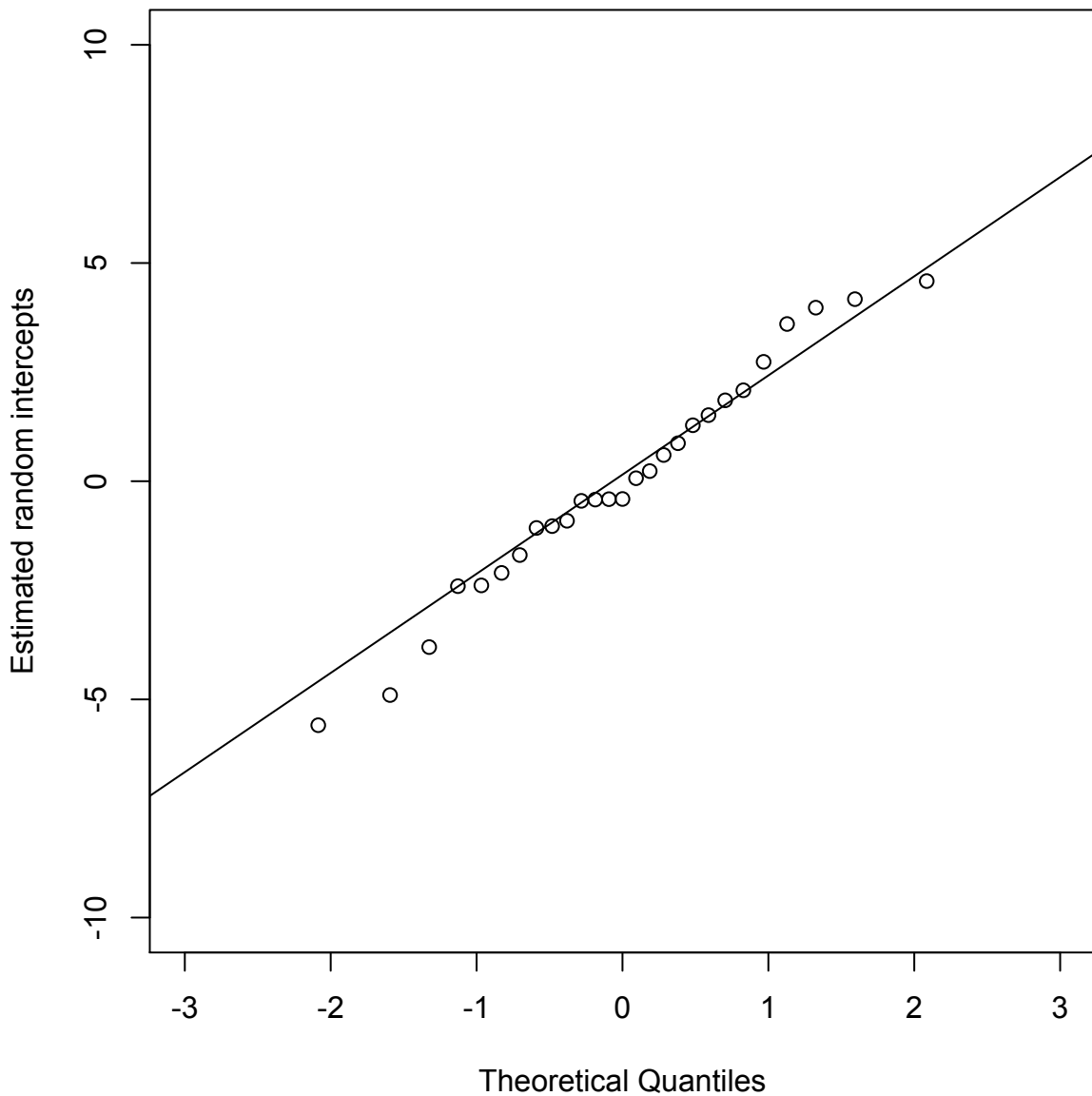

**Residuals**

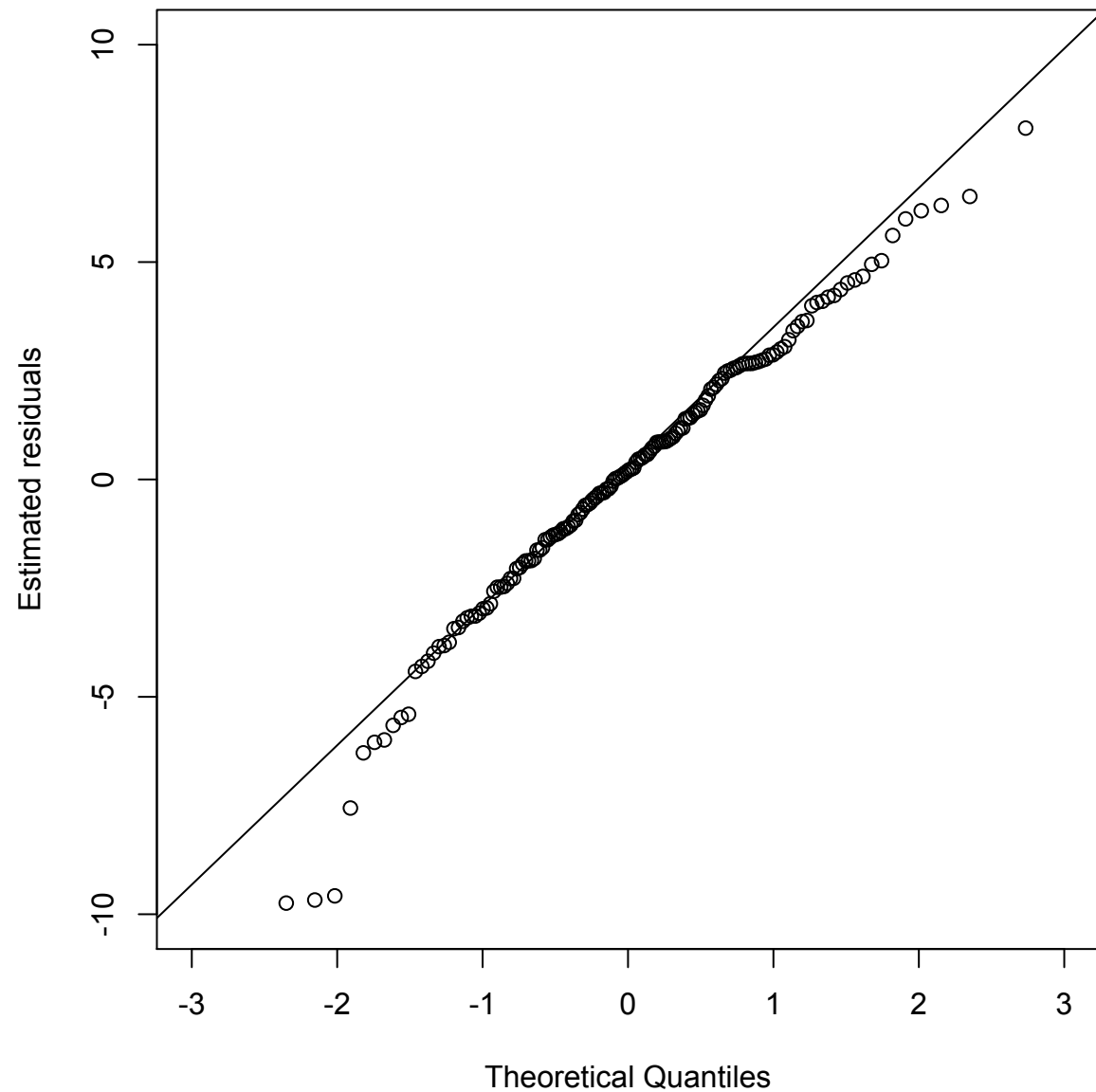

Supplement: S1 File — The code can be executed with the free R software (GNU General Public License). The plots, included in the Results section, can be created directly from the study data with the file PEP_Plots.R. For the Raincloud plots, additional source files are needed from Allen et al. [54]. Further, the ZIP file contains text files with the R software console output, showing the executed code and the results (*.txt file extensions). Lastly, S1 File contains PDF files for all dependent variables with significant predictor variables. The PDF files contain two plots each, showing the QQ-Plots for Random Intercepts and Residuals from the linear mixed-effect model. (ZIP) [file pone.0239553.s003.zip › 01_dPEP.pdf]

**Random intercepts**

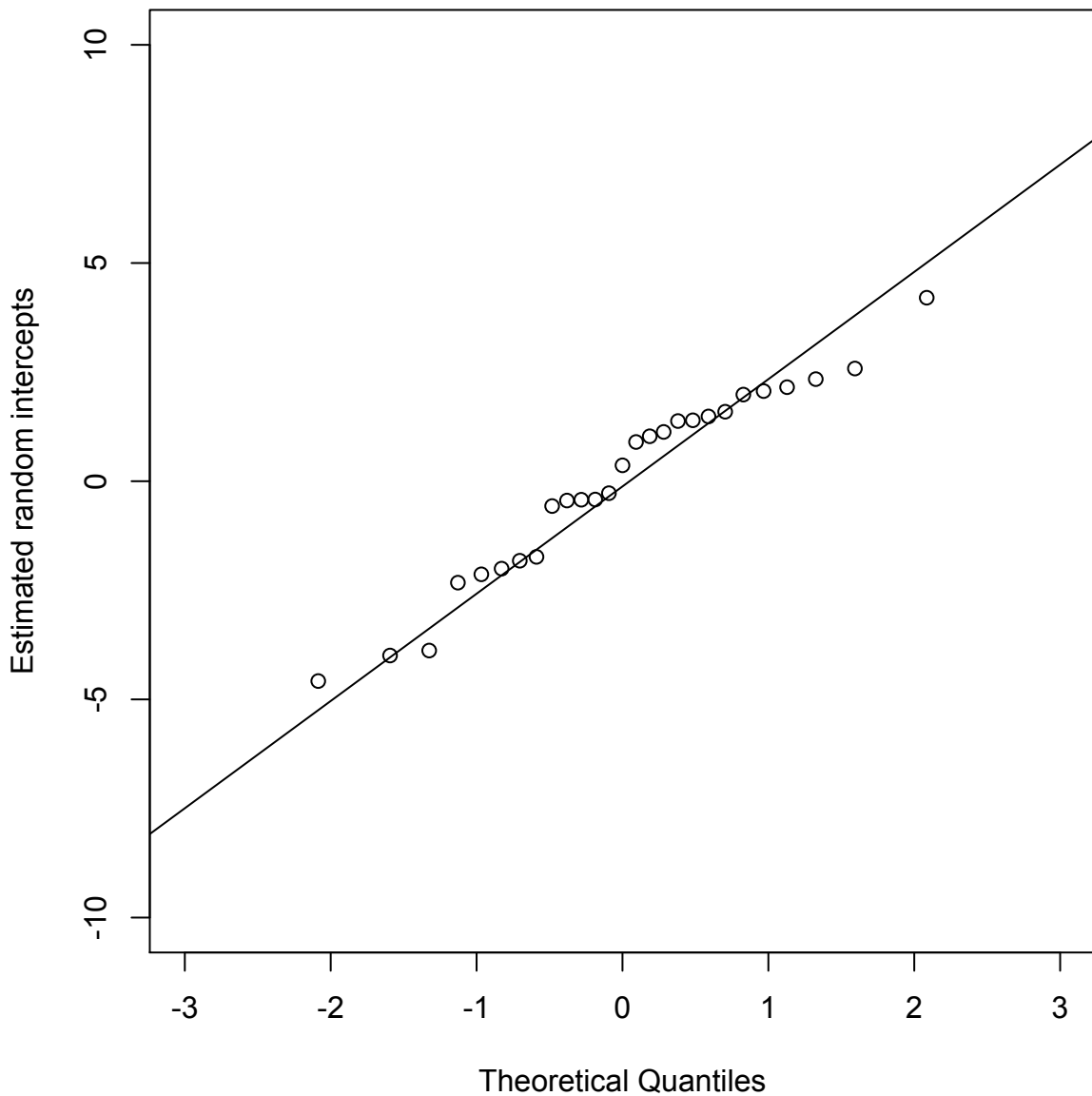

**Residuals**

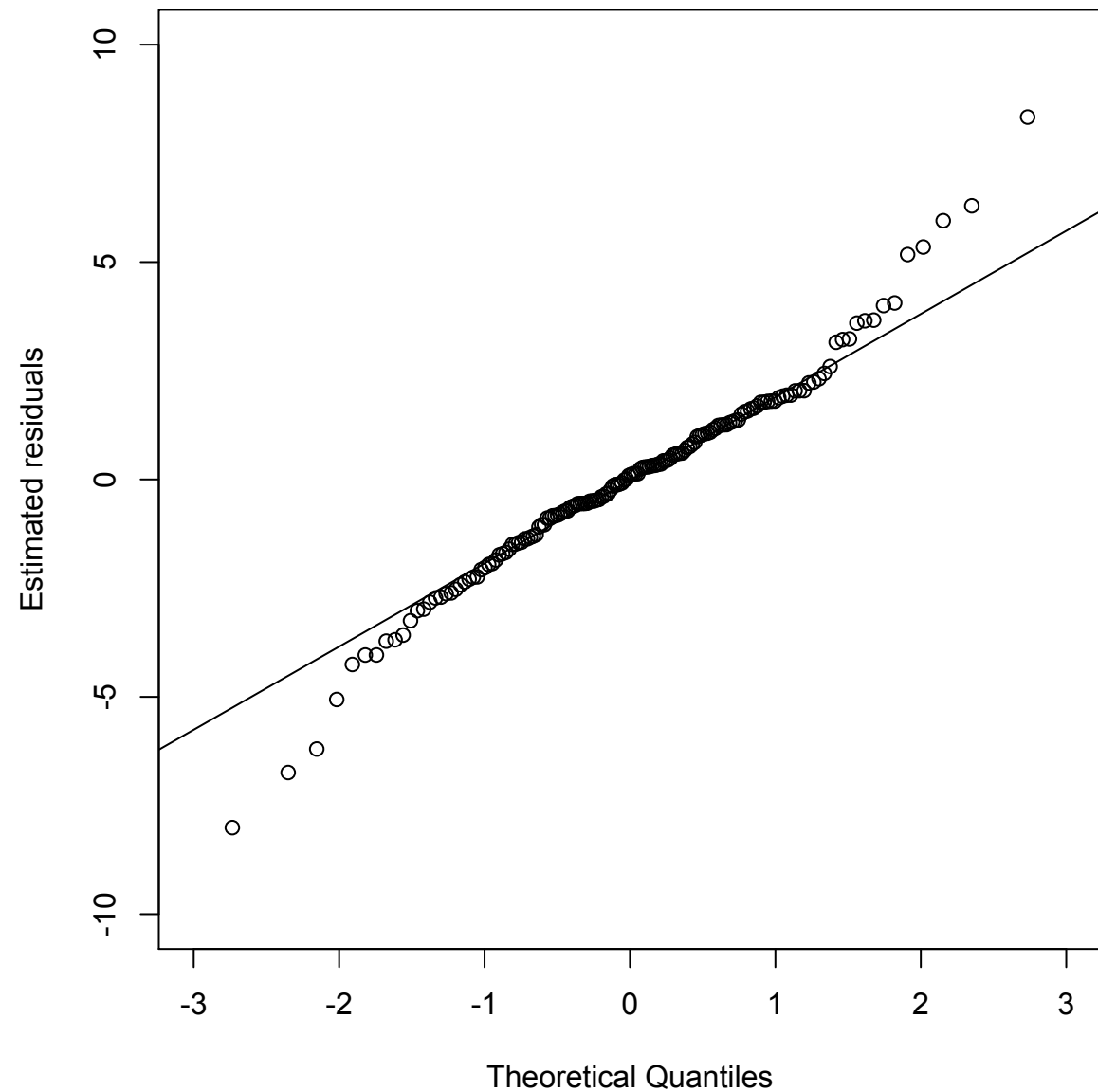

Supplement: S1 File — The code can be executed with the free R software (GNU General Public License). The plots, included in the Results section, can be created directly from the study data with the file PEP_Plots.R. For the Raincloud plots, additional source files are needed from Allen et al. [54]. Further, the ZIP file contains text files with the R software console output, showing the executed code and the results (*.txt file extensions). Lastly, S1 File contains PDF files for all dependent variables with significant predictor variables. The PDF files contain two plots each, showing the QQ-Plots for Random Intercepts and Residuals from the linear mixed-effect model. (ZIP) [file pone.0239553.s003.zip › 02_dHR.pdf]

**Random intercepts**

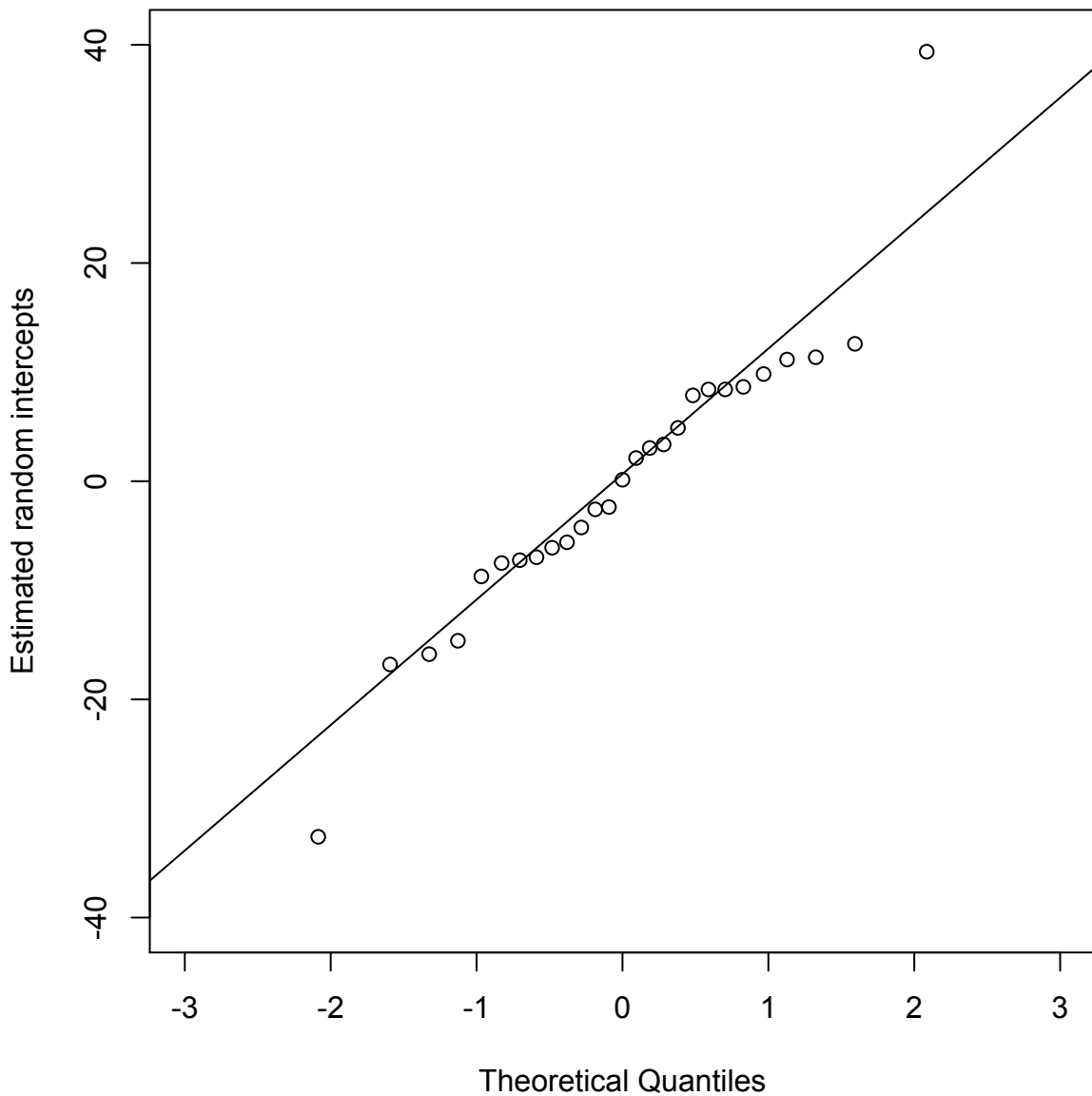

**Residuals**

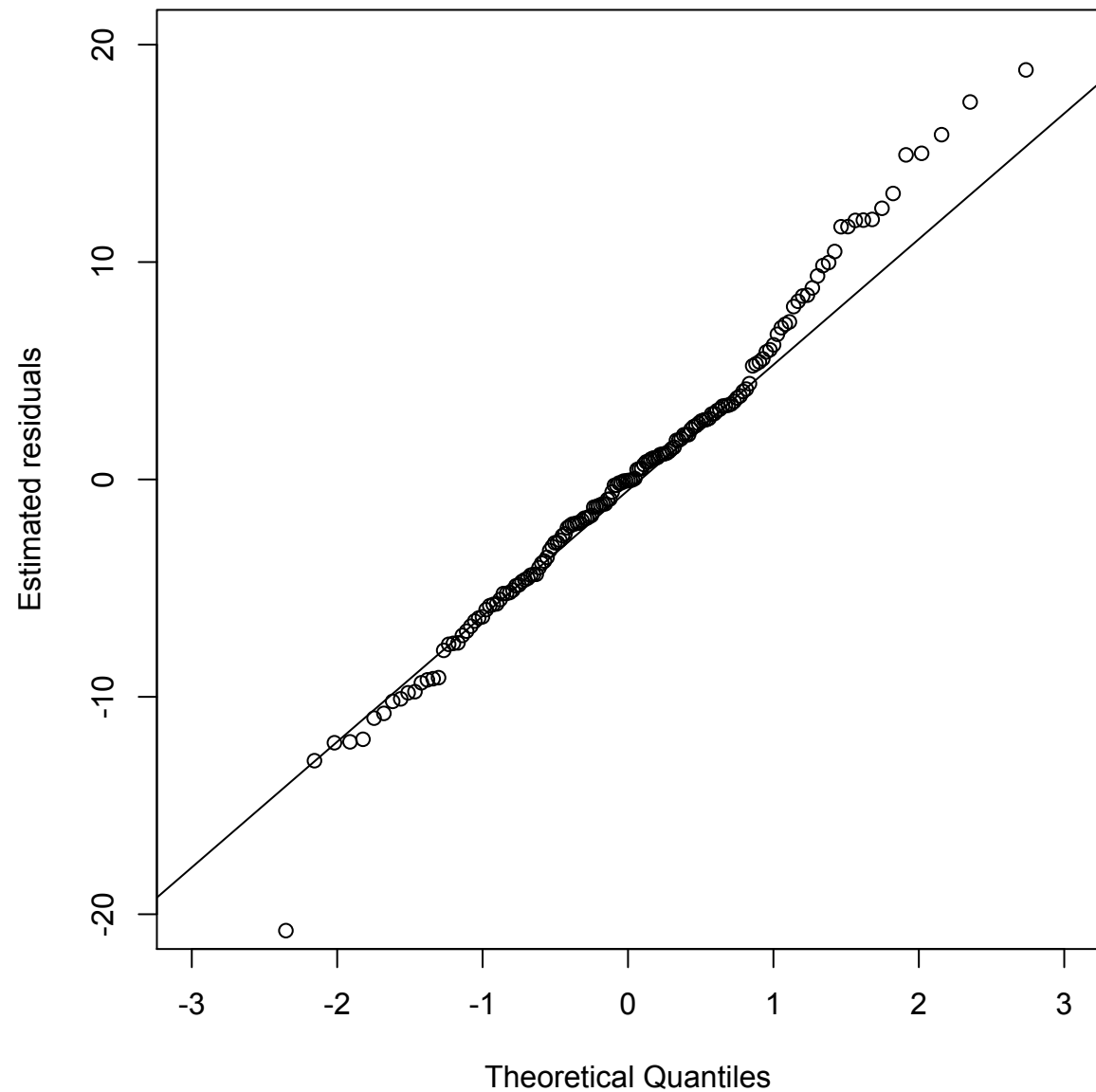

Supplement: S1 File — The code can be executed with the free R software (GNU General Public License). The plots, included in the Results section, can be created directly from the study data with the file PEP_Plots.R. For the Raincloud plots, additional source files are needed from Allen et al. [54]. Further, the ZIP file contains text files with the R software console output, showing the executed code and the results (*.txt file extensions). Lastly, S1 File contains PDF files for all dependent variables with significant predictor variables. The PDF files contain two plots each, showing the QQ-Plots for Random Intercepts and Residuals from the linear mixed-effect model. (ZIP) [file pone.0239553.s003.zip › 04_PEP_Rest.pdf]

**Random intercepts**

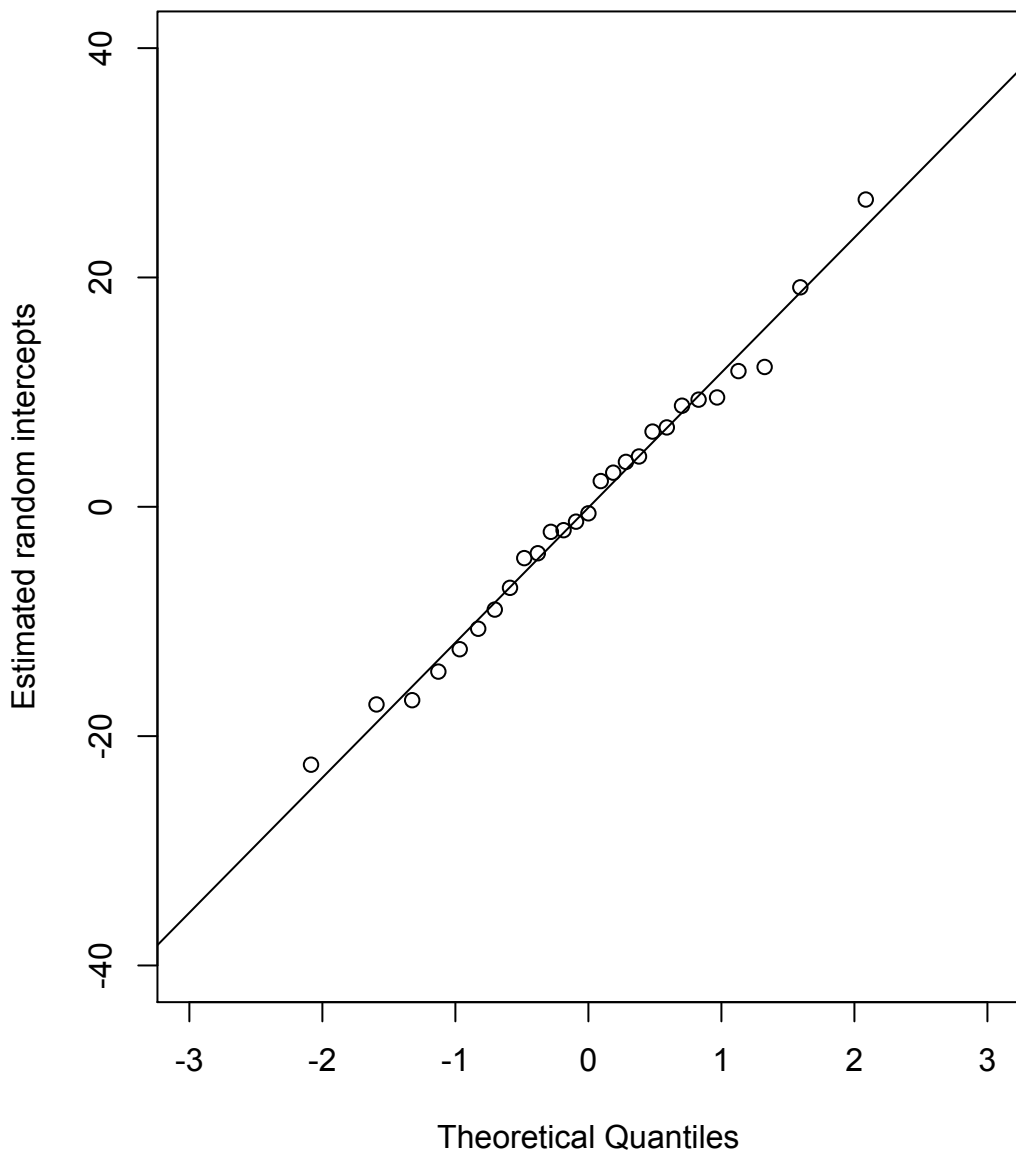

**Residuals**

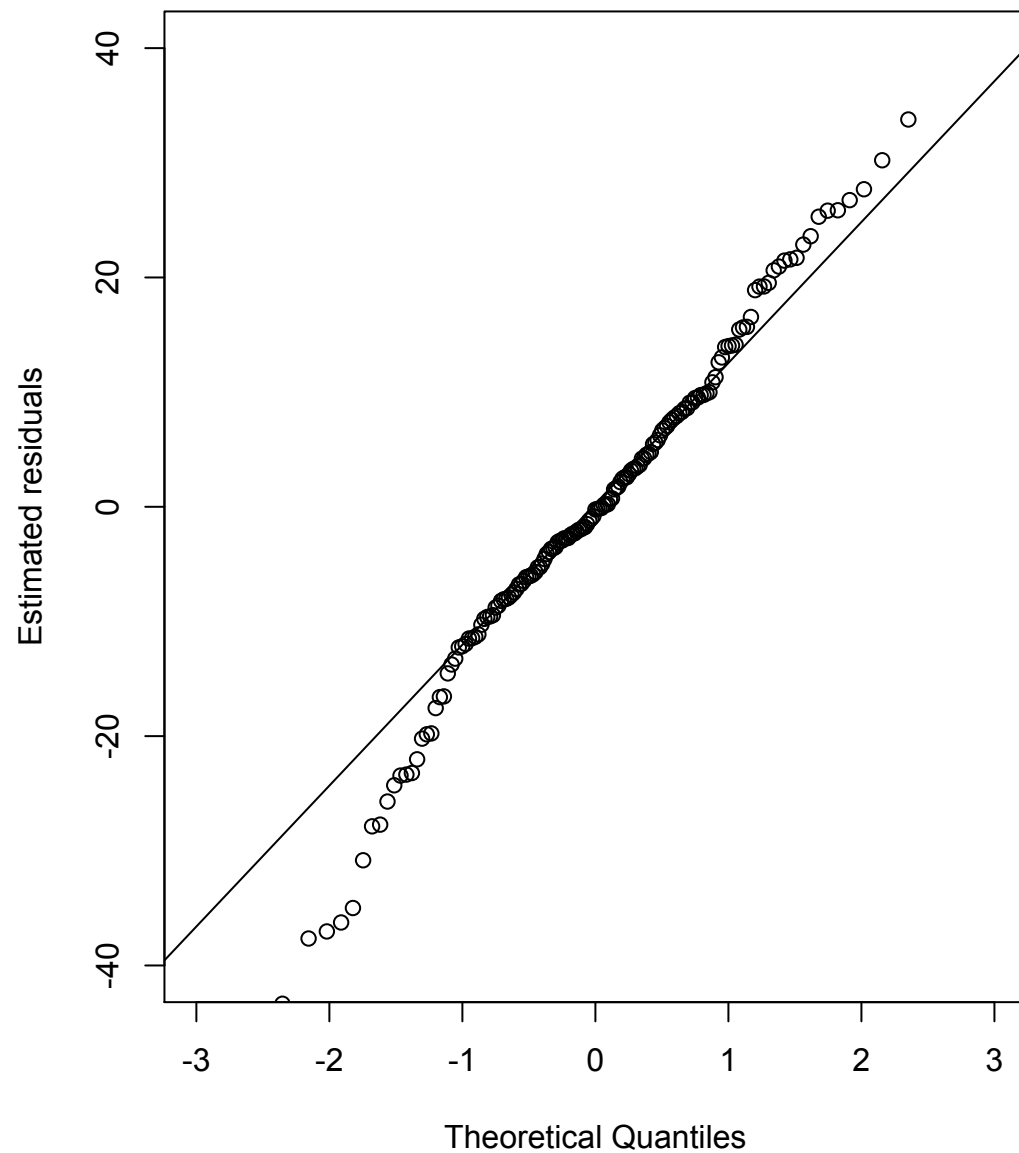

Supplement: S1 File — The code can be executed with the free R software (GNU General Public License). The plots, included in the Results section, can be created directly from the study data with the file PEP_Plots.R. For the Raincloud plots, additional source files are needed from Allen et al. [54]. Further, the ZIP file contains text files with the R software console output, showing the executed code and the results (*.txt file extensions). Lastly, S1 File contains PDF files for all dependent variables with significant predictor variables. The PDF files contain two plots each, showing the QQ-Plots for Random Intercepts and Residuals from the linear mixed-effect model. (ZIP) [file pone.0239553.s003.zip › 06_Lvet_Rest.pdf]

**Random intercepts**

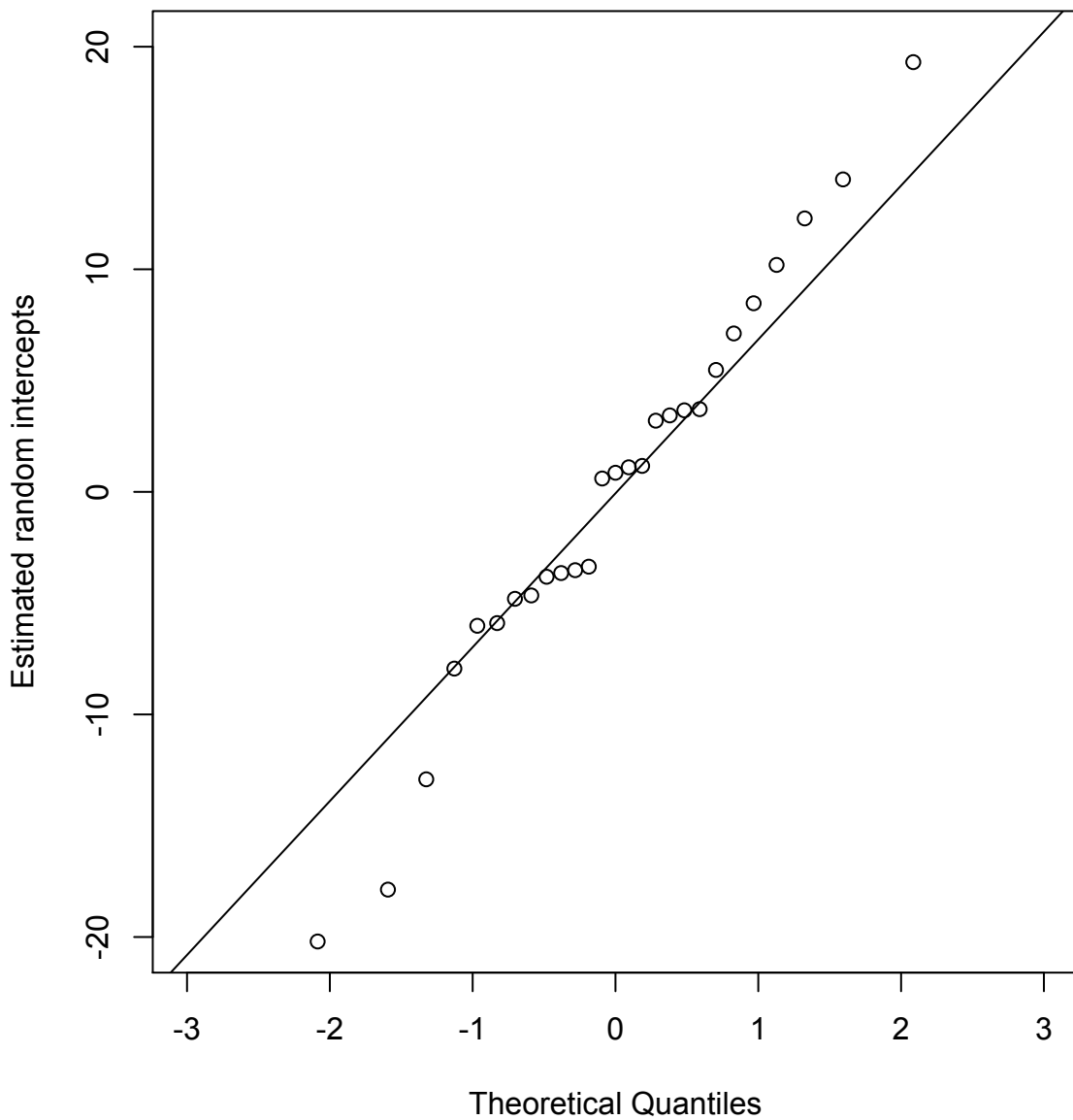

**Residuals**

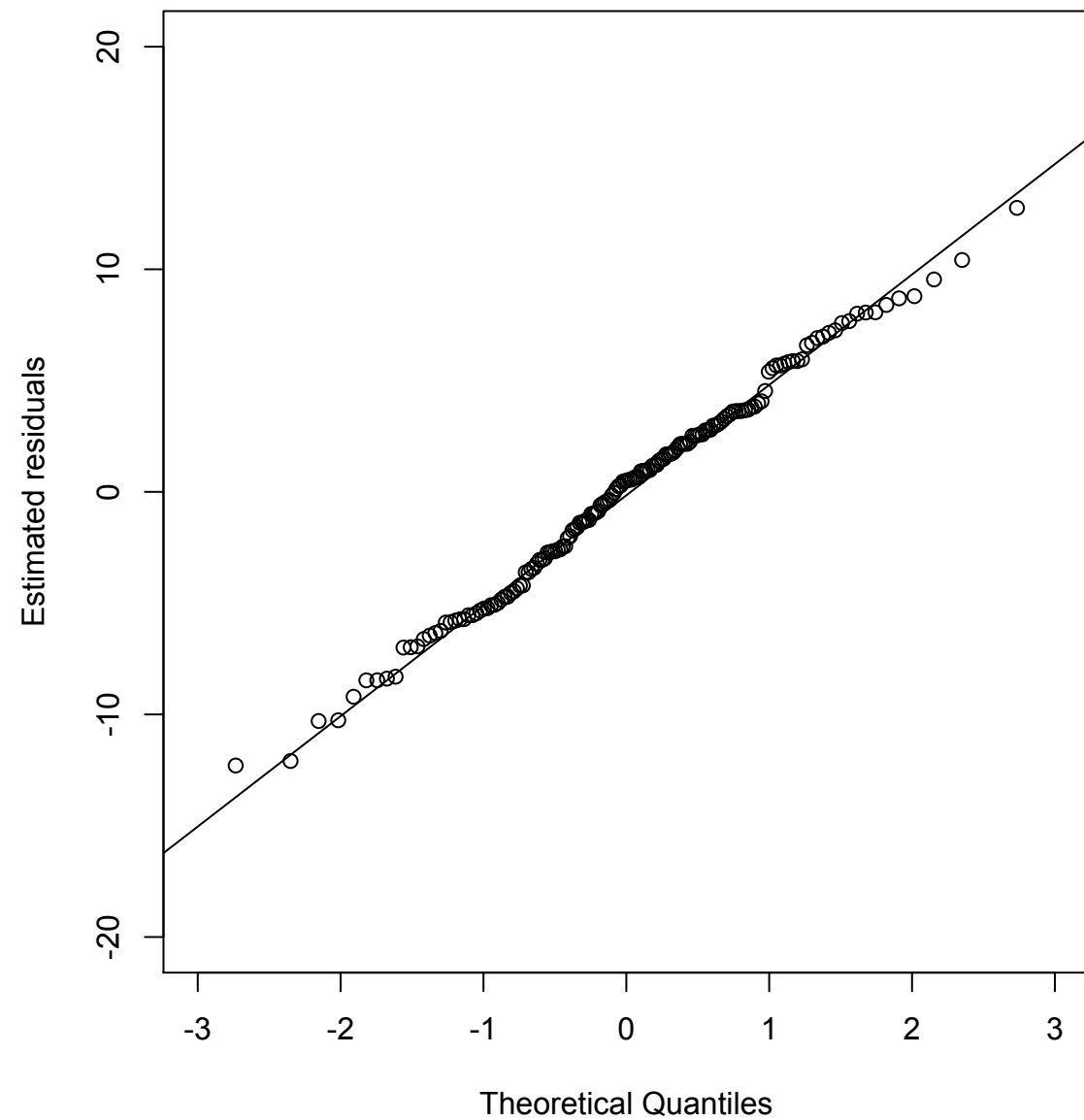

Supplement: S1 File — The code can be executed with the free R software (GNU General Public License). The plots, included in the Results section, can be created directly from the study data with the file PEP_Plots.R. For the Raincloud plots, additional source files are needed from Allen et al. [54]. Further, the ZIP file contains text files with the R software console output, showing the executed code and the results (*.txt file extensions). Lastly, S1 File contains PDF files for all dependent variables with significant predictor variables. The PDF files contain two plots each, showing the QQ-Plots for Random Intercepts and Residuals from the linear mixed-effect model. (ZIP) [file pone.0239553.s003.zip › 07_Raw_TLX.pdf]

**Random intercepts**

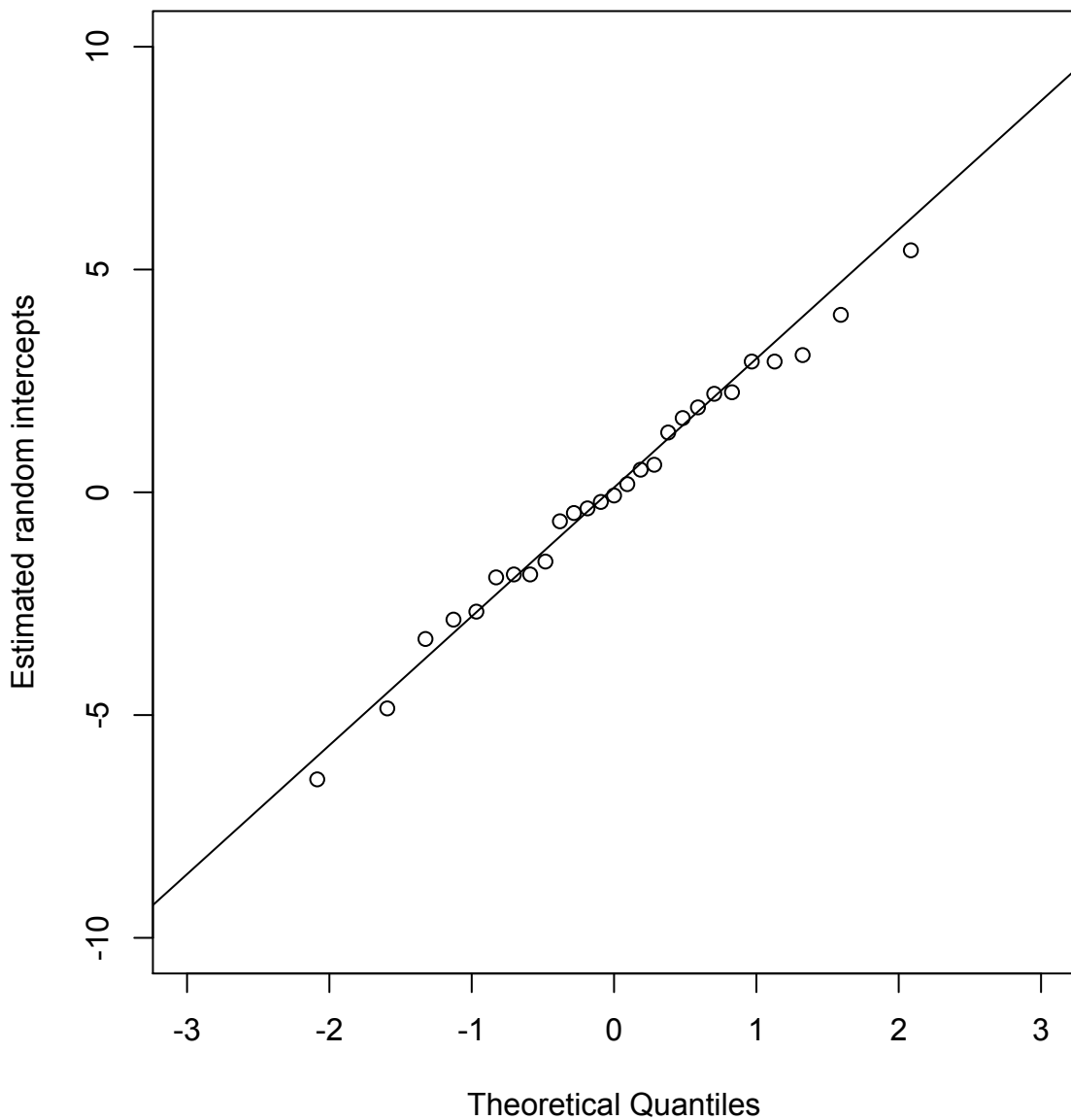

**Residuals**

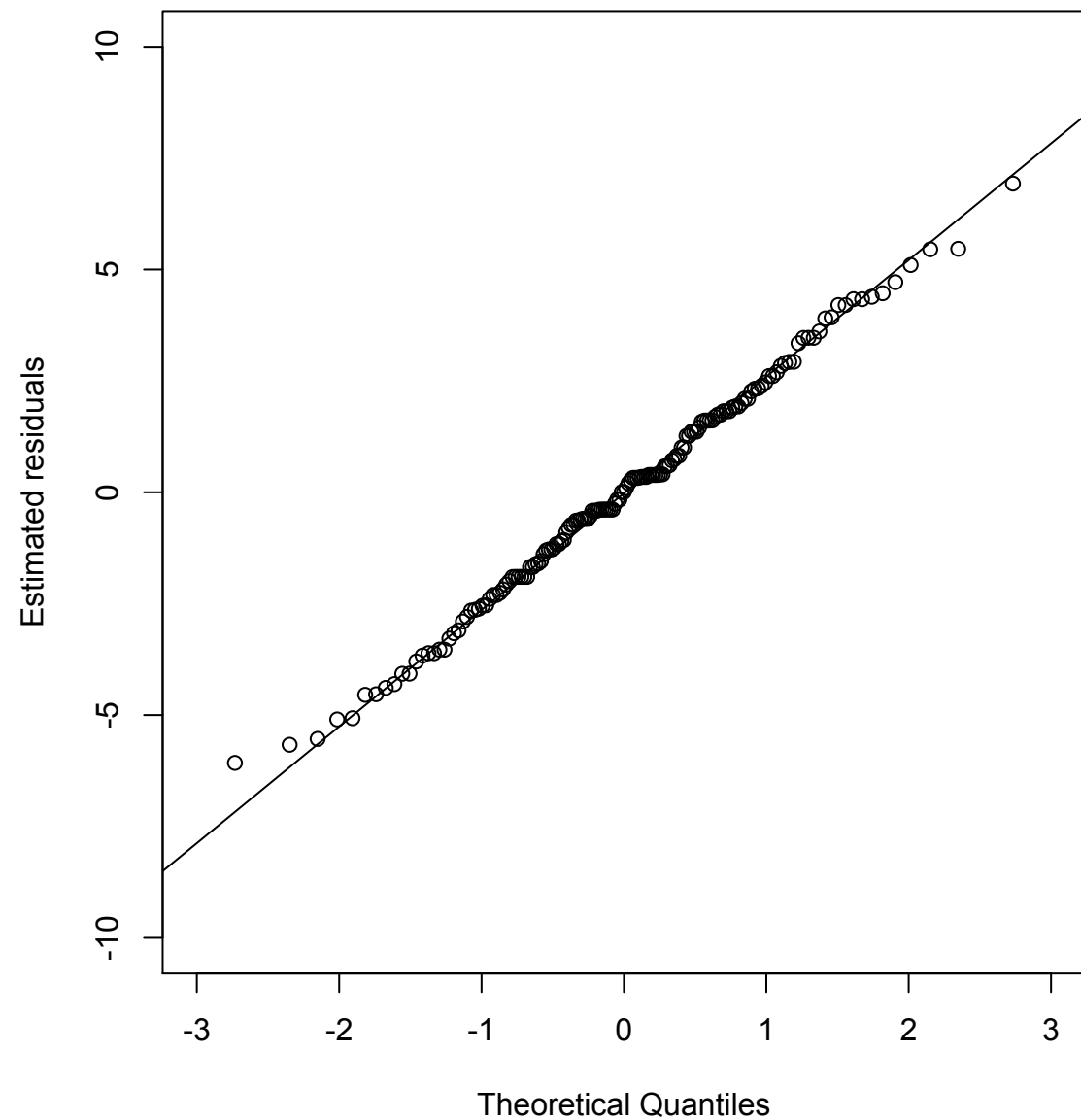

Supplement: S1 File — The code can be executed with the free R software (GNU General Public License). The plots, included in the Results section, can be created directly from the study data with the file PEP_Plots.R. For the Raincloud plots, additional source files are needed from Allen et al. [54]. Further, the ZIP file contains text files with the R software console output, showing the executed code and the results (*.txt file extensions). Lastly, S1 File contains PDF files for all dependent variables with significant predictor variables. The PDF files contain two plots each, showing the QQ-Plots for Random Intercepts and Residuals from the linear mixed-effect model. (ZIP) [file pone.0239553.s003.zip › 08_TLX_1.pdf]

**Random intercepts**

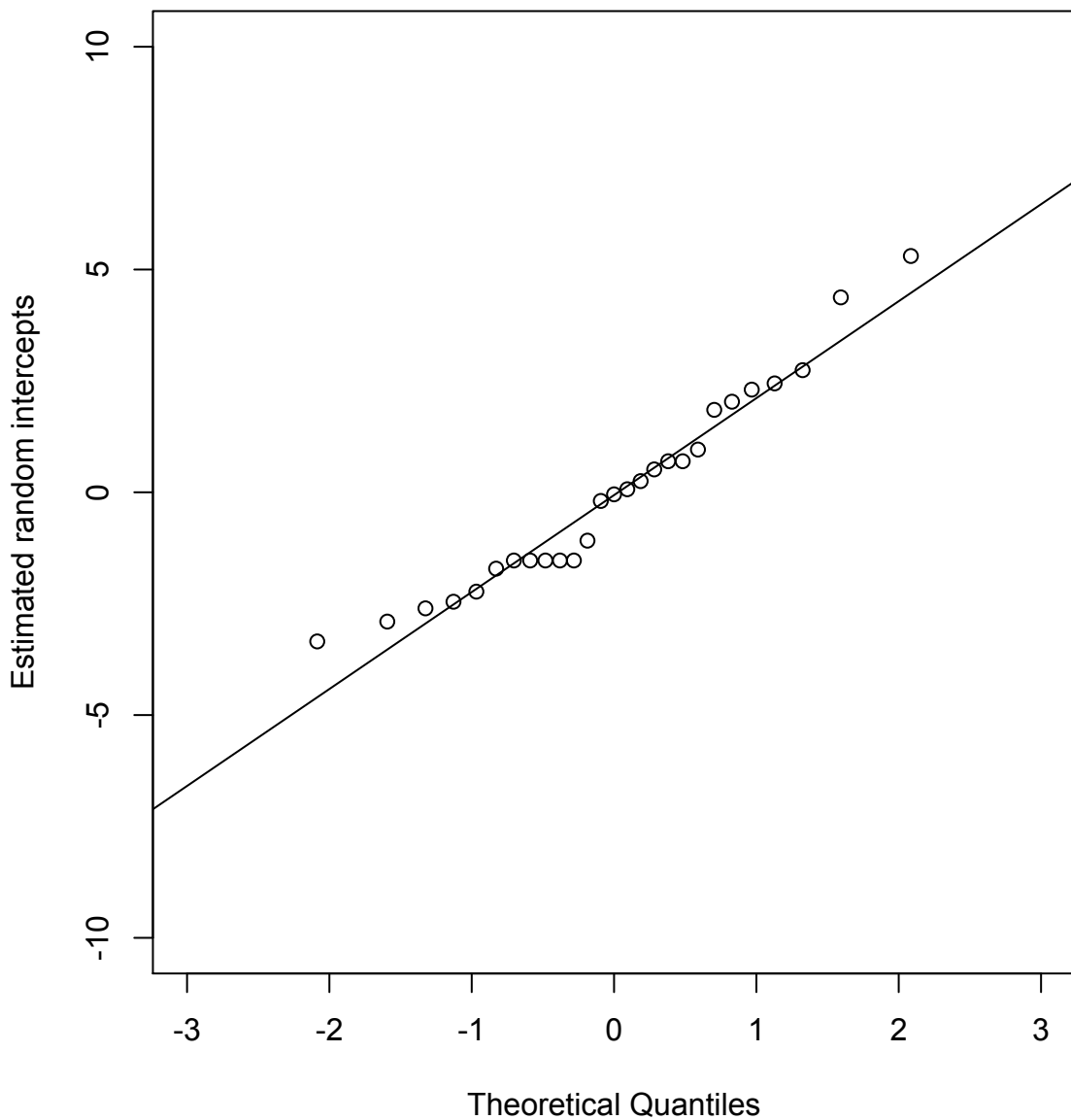

**Residuals**

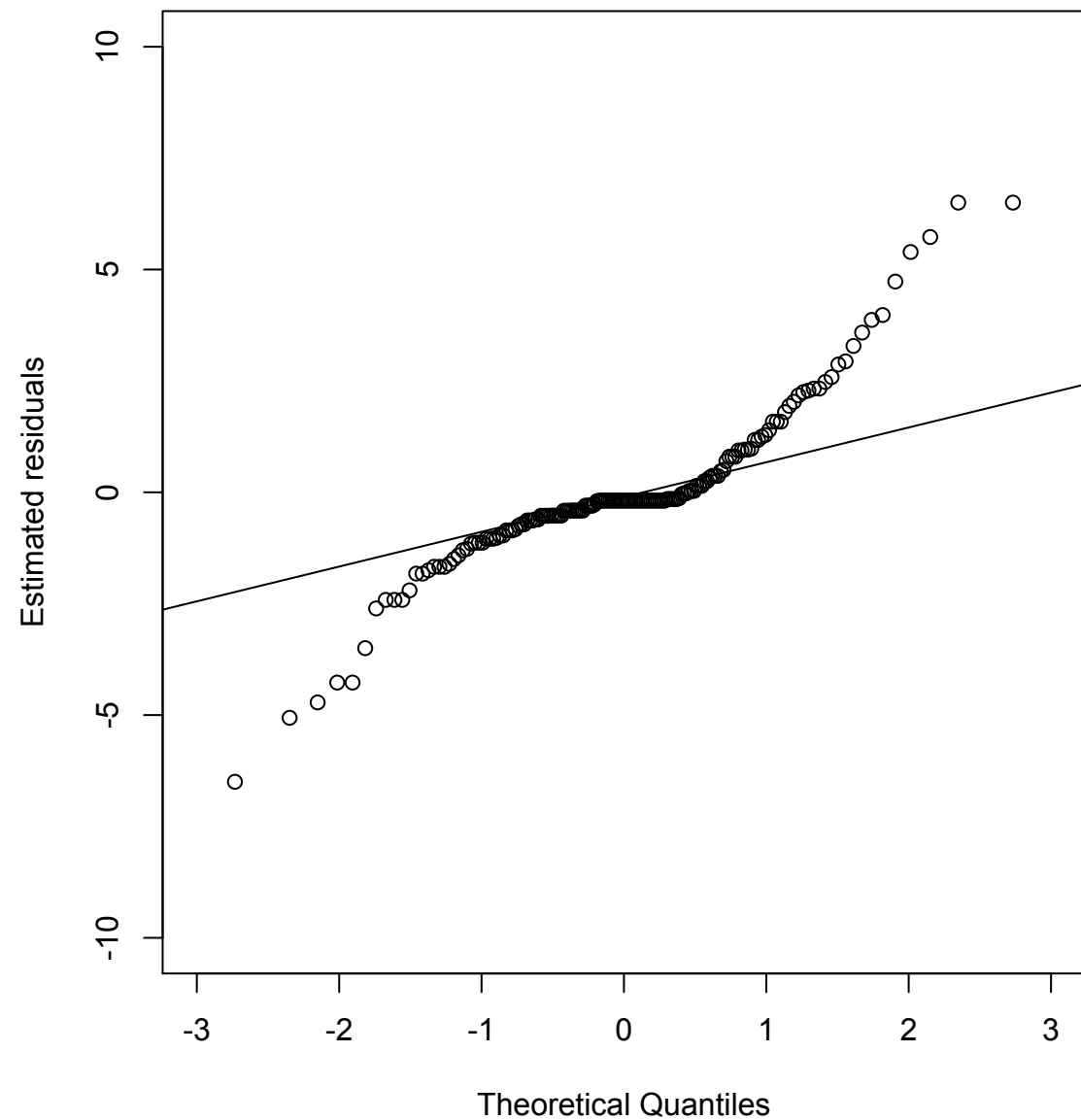

Supplement: S1 File — The code can be executed with the free R software (GNU General Public License). The plots, included in the Results section, can be created directly from the study data with the file PEP_Plots.R. For the Raincloud plots, additional source files are needed from Allen et al. [54]. Further, the ZIP file contains text files with the R software console output, showing the executed code and the results (*.txt file extensions). Lastly, S1 File contains PDF files for all dependent variables with significant predictor variables. The PDF files contain two plots each, showing the QQ-Plots for Random Intercepts and Residuals from the linear mixed-effect model. (ZIP) [file pone.0239553.s003.zip › 09_TLX_2.pdf]

**Random intercepts**

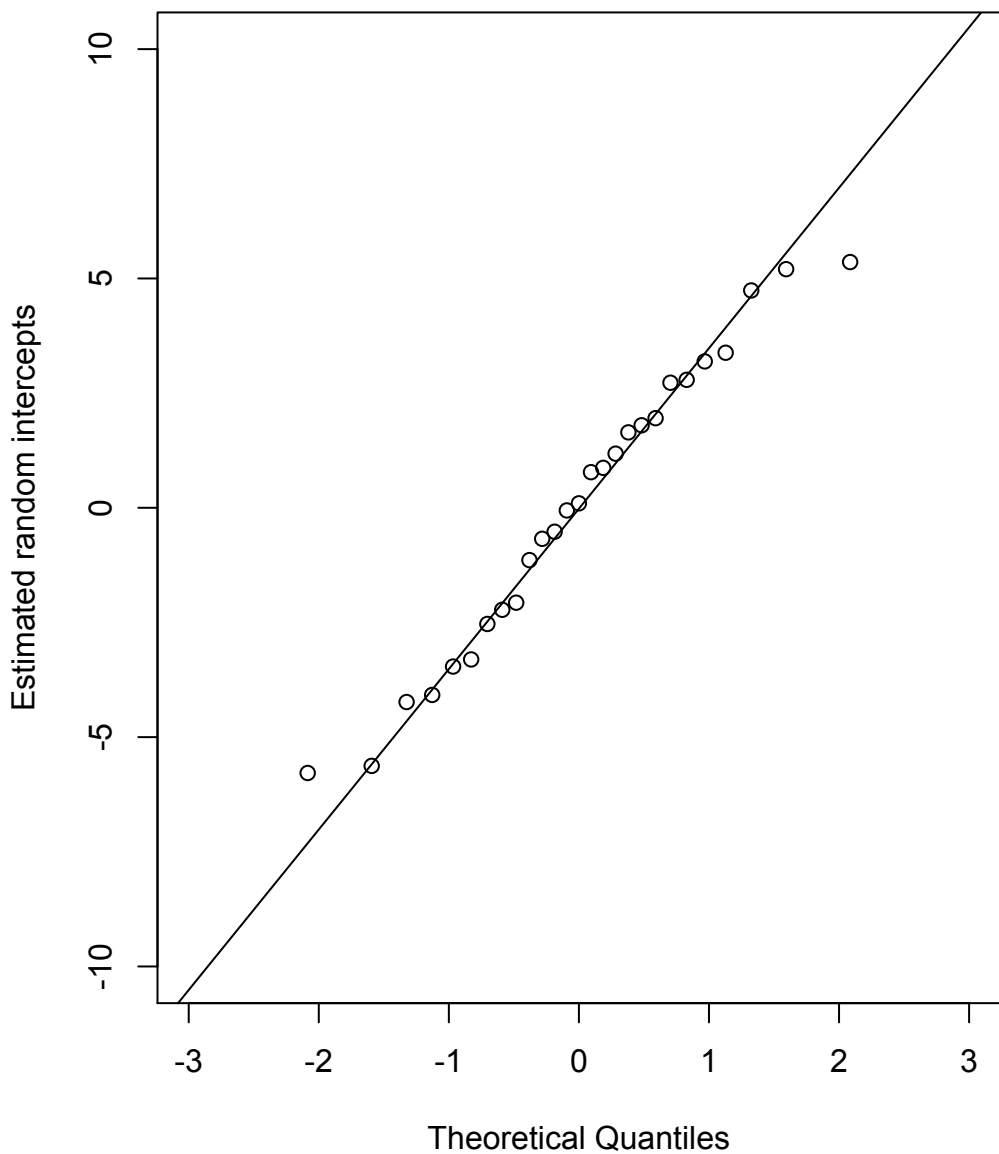

**Residuals**

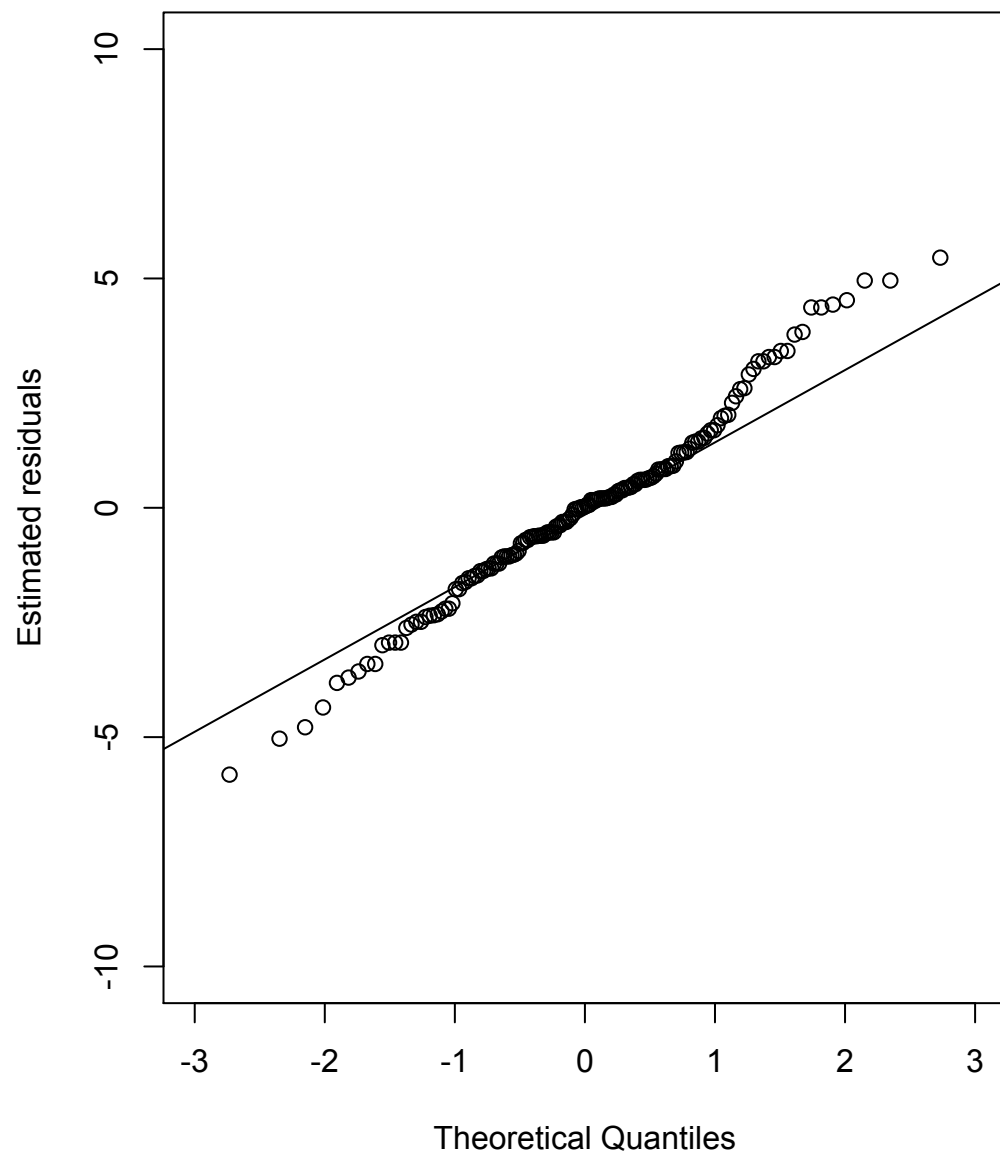

Supplement: S1 File — The code can be executed with the free R software (GNU General Public License). The plots, included in the Results section, can be created directly from the study data with the file PEP_Plots.R. For the Raincloud plots, additional source files are needed from Allen et al. [54]. Further, the ZIP file contains text files with the R software console output, showing the executed code and the results (*.txt file extensions). Lastly, S1 File contains PDF files for all dependent variables with significant predictor variables. The PDF files contain two plots each, showing the QQ-Plots for Random Intercepts and Residuals from the linear mixed-effect model. (ZIP) [file pone.0239553.s003.zip › 10_TLX_3.pdf]

**Random intercepts**

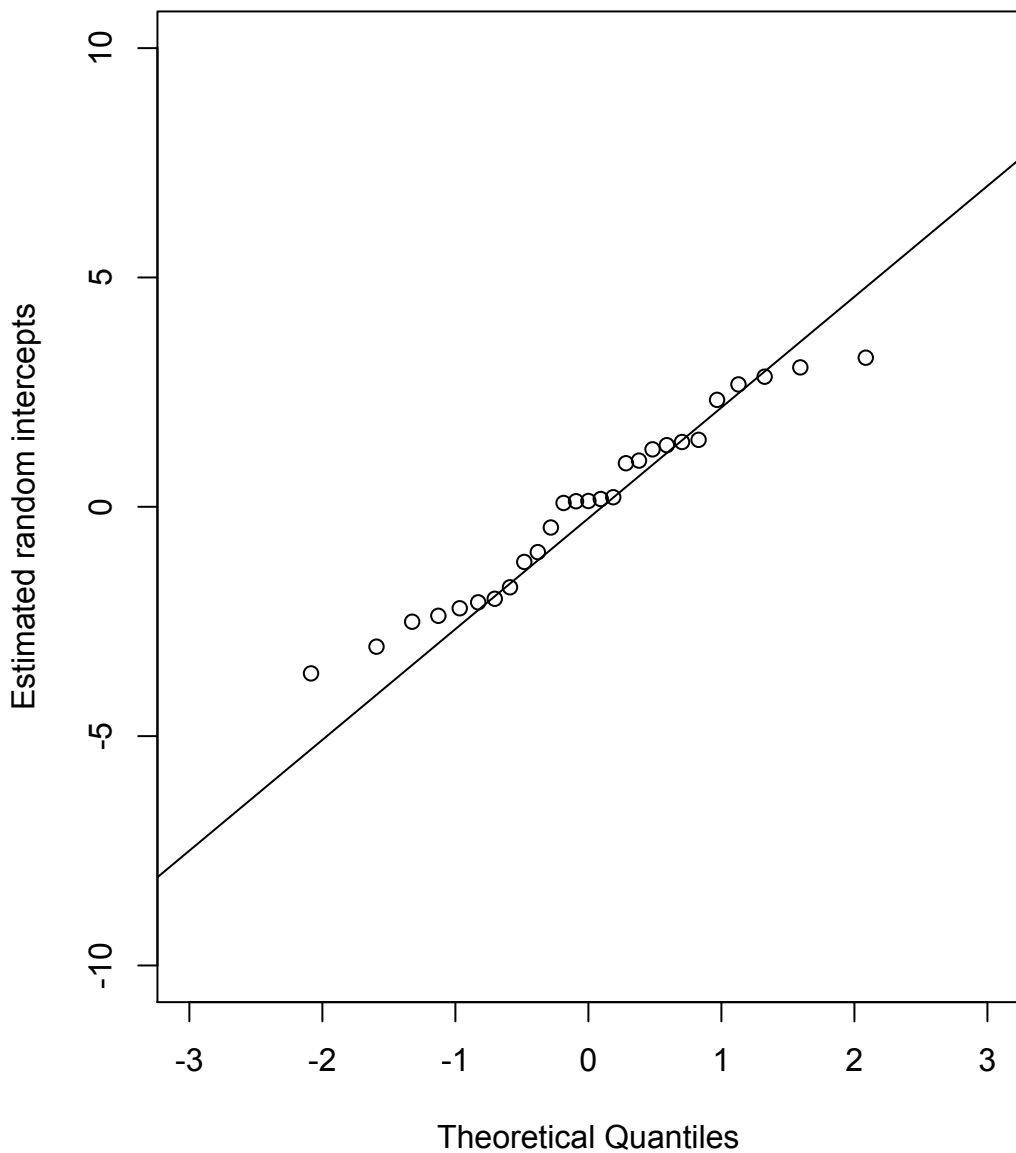

**Residuals**

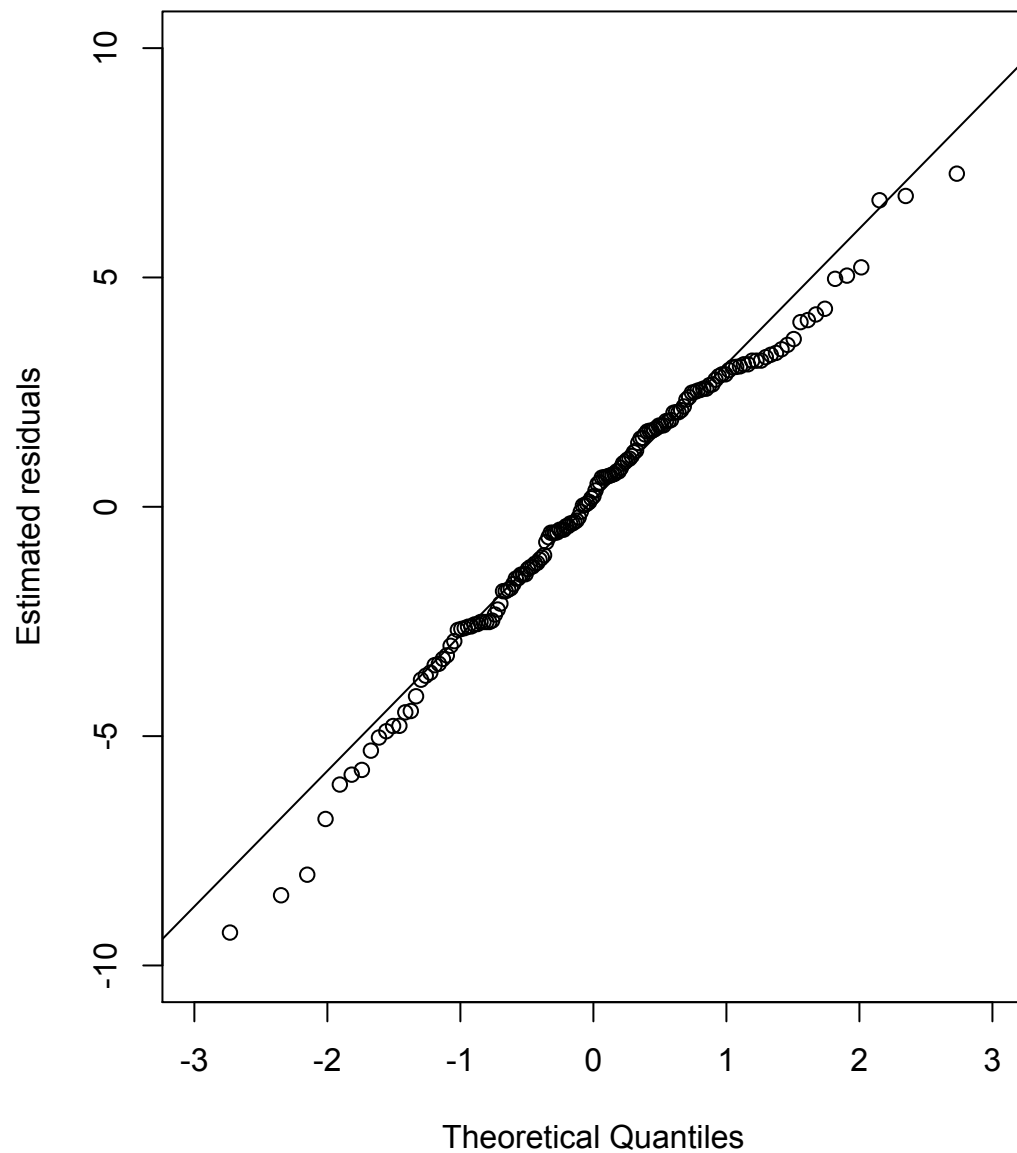

Supplement: S1 File — The code can be executed with the free R software (GNU General Public License). The plots, included in the Results section, can be created directly from the study data with the file PEP_Plots.R. For the Raincloud plots, additional source files are needed from Allen et al. [54]. Further, the ZIP file contains text files with the R software console output, showing the executed code and the results (*.txt file extensions). Lastly, S1 File contains PDF files for all dependent variables with significant predictor variables. The PDF files contain two plots each, showing the QQ-Plots for Random Intercepts and Residuals from the linear mixed-effect model. (ZIP) [file pone.0239553.s003.zip › 11_TLX_4.pdf]

**Random intercepts**

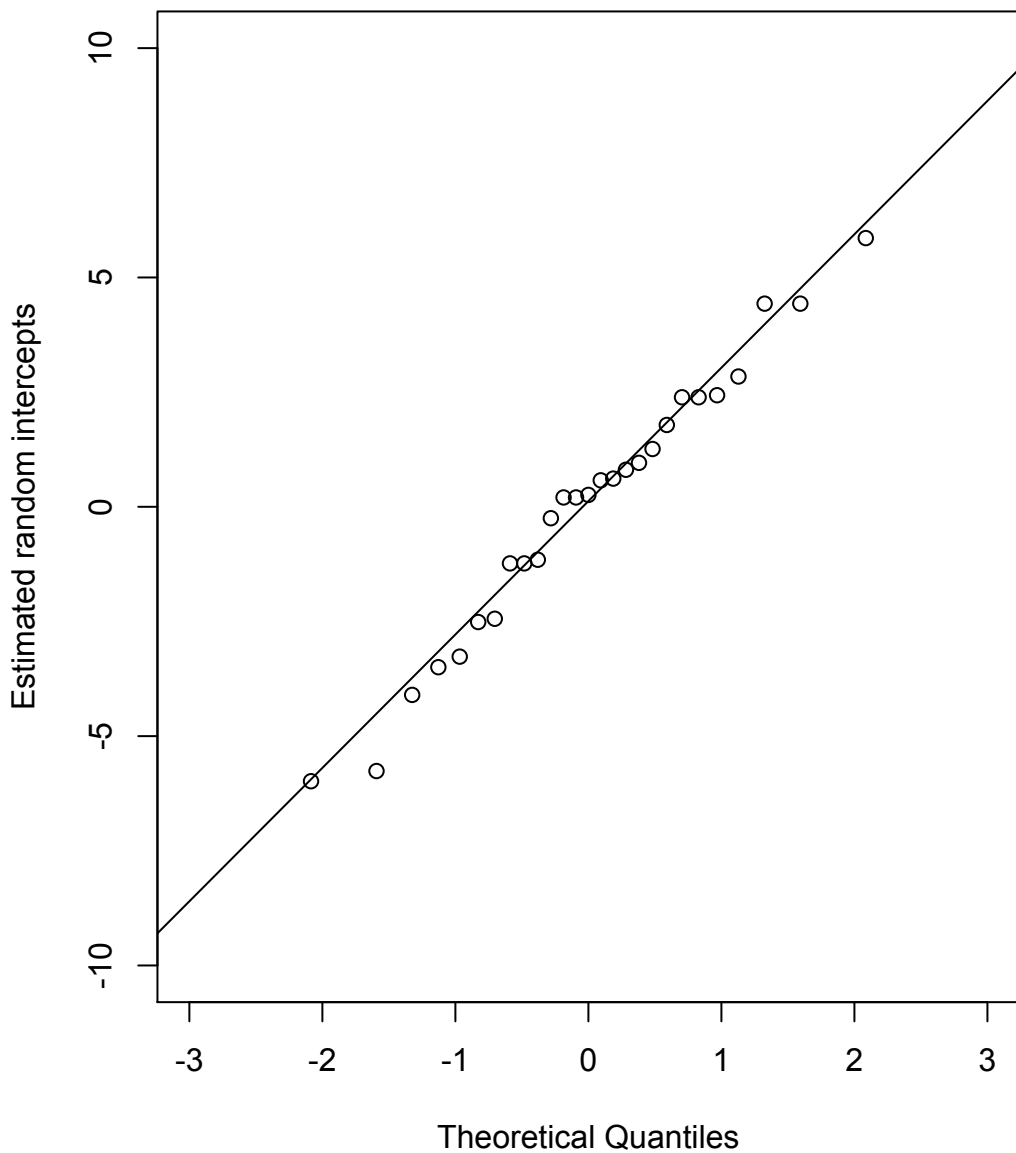

**Residuals**

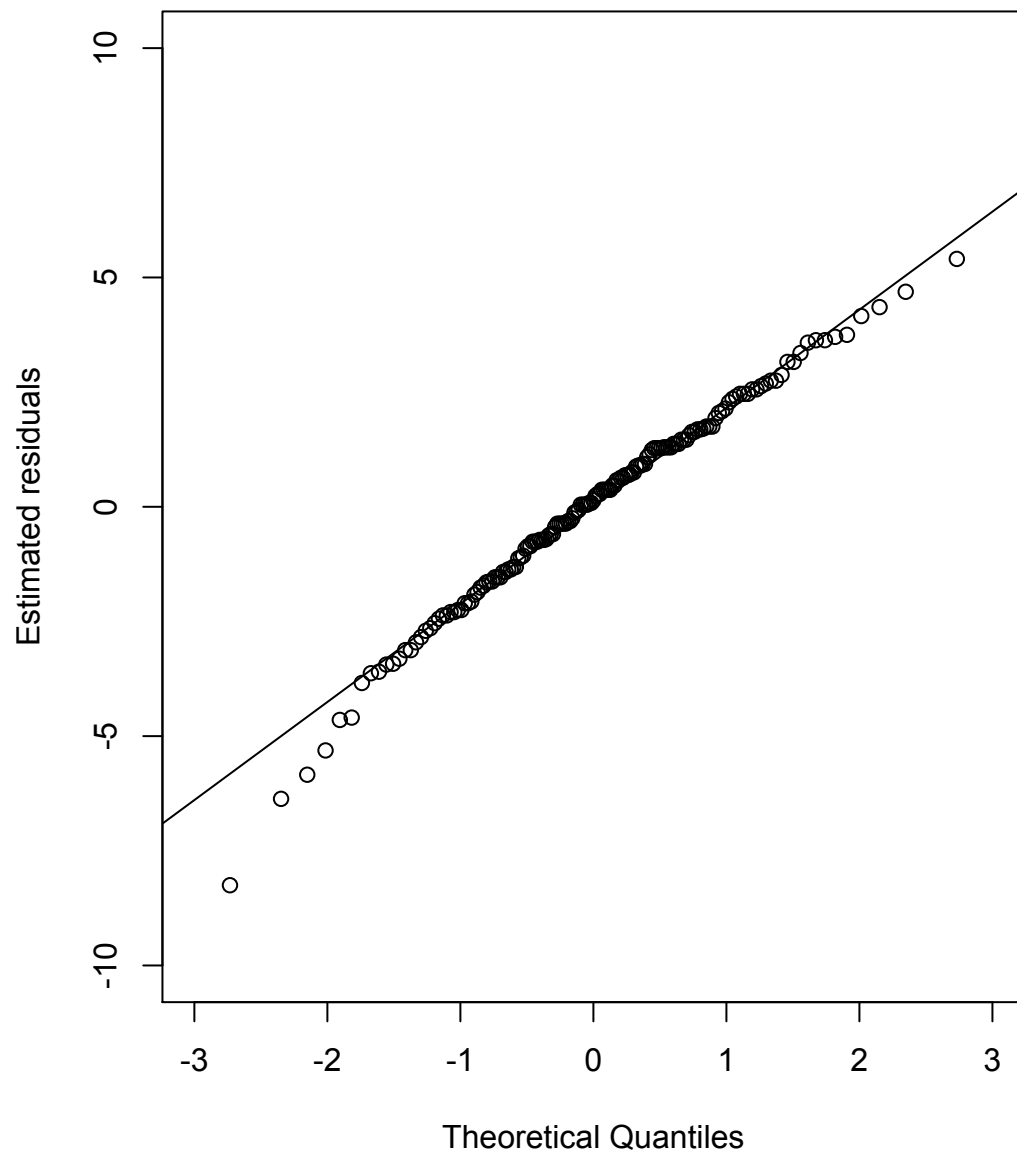

Supplement: S1 File — The code can be executed with the free R software (GNU General Public License). The plots, included in the Results section, can be created directly from the study data with the file PEP_Plots.R. For the Raincloud plots, additional source files are needed from Allen et al. [54]. Further, the ZIP file contains text files with the R software console output, showing the executed code and the results (*.txt file extensions). Lastly, S1 File contains PDF files for all dependent variables with significant predictor variables. The PDF files contain two plots each, showing the QQ-Plots for Random Intercepts and Residuals from the linear mixed-effect model. (ZIP) [file pone.0239553.s003.zip › 12_TLX_5.pdf]

**Random intercepts**

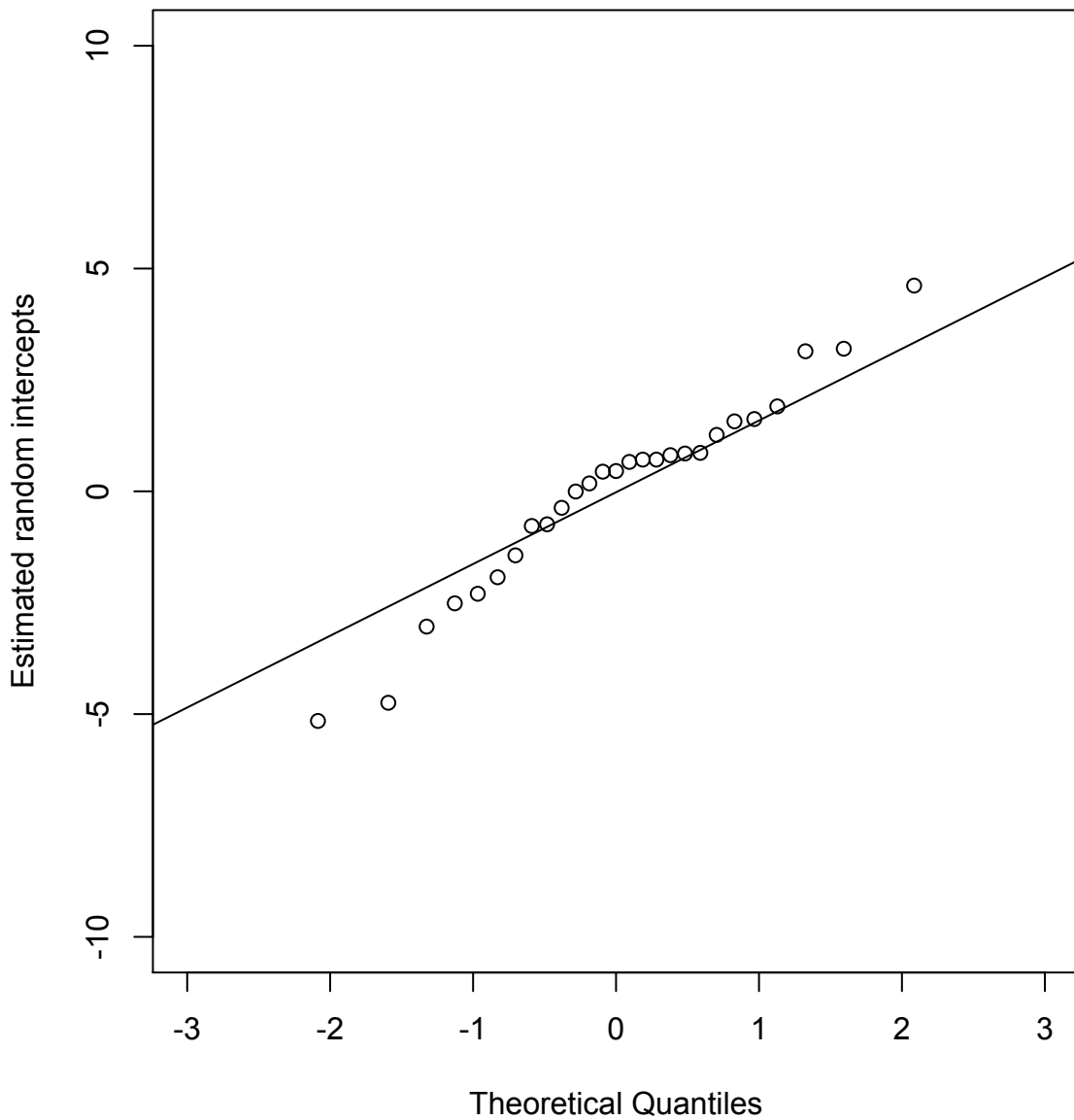

**Residuals**

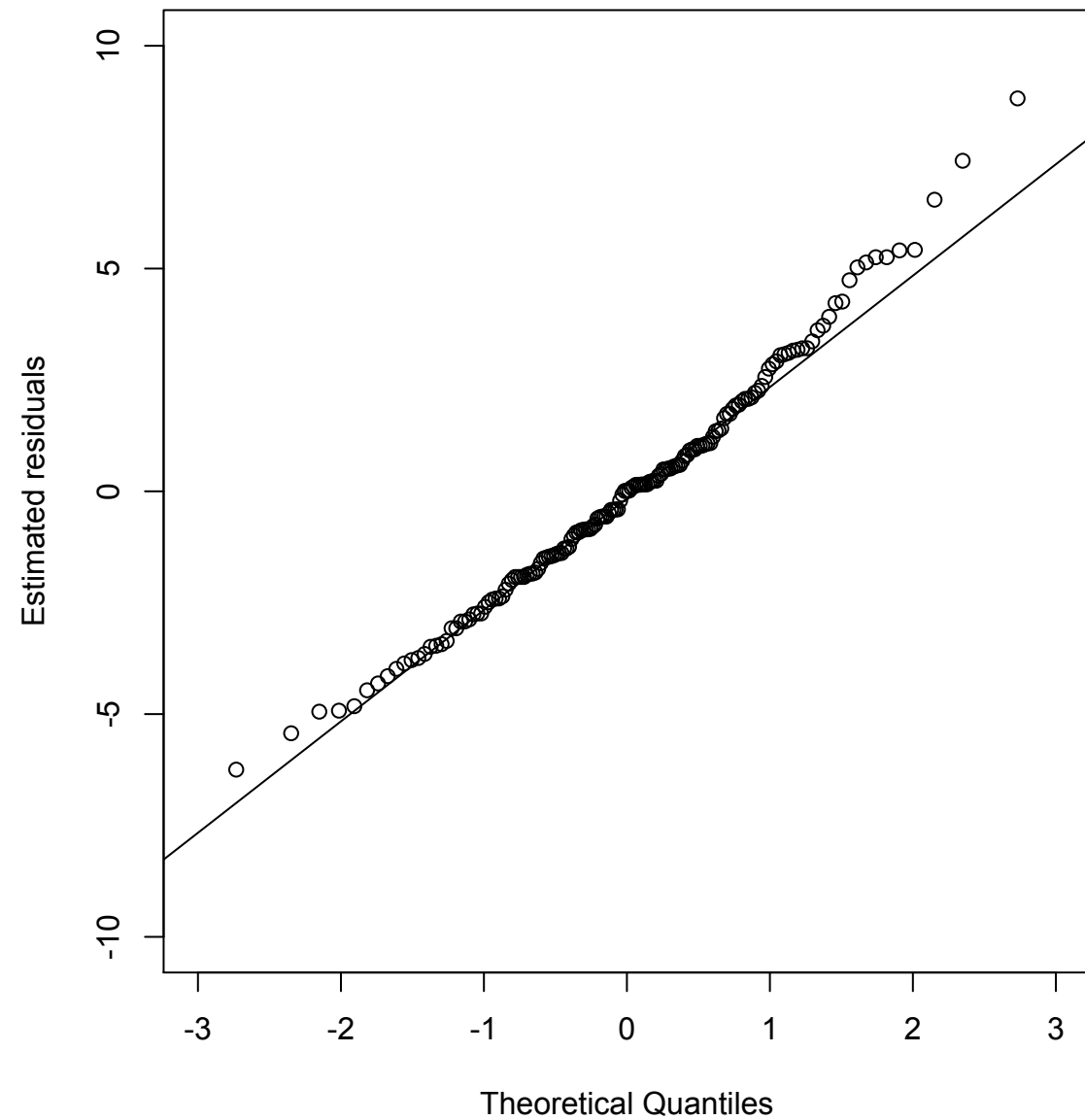

Supplement: S1 File — The code can be executed with the free R software (GNU General Public License). The plots, included in the Results section, can be created directly from the study data with the file PEP_Plots.R. For the Raincloud plots, additional source files are needed from Allen et al. [54]. Further, the ZIP file contains text files with the R software console output, showing the executed code and the results (*.txt file extensions). Lastly, S1 File contains PDF files for all dependent variables with significant predictor variables. The PDF files contain two plots each, showing the QQ-Plots for Random Intercepts and Residuals from the linear mixed-effect model. (ZIP) [file pone.0239553.s003.zip › 13_TLX_6.pdf]

**Random intercepts**

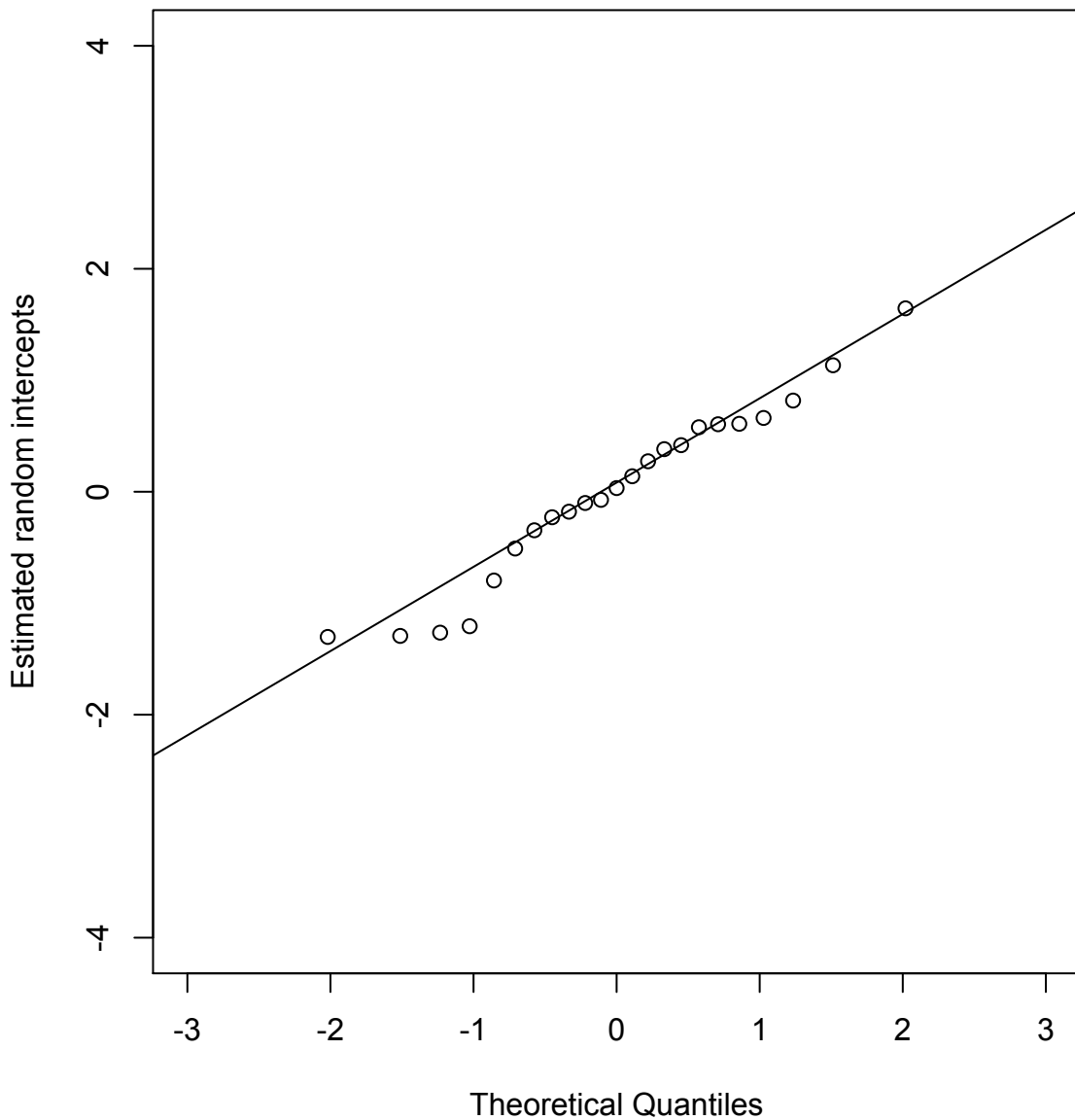

**Residuals**

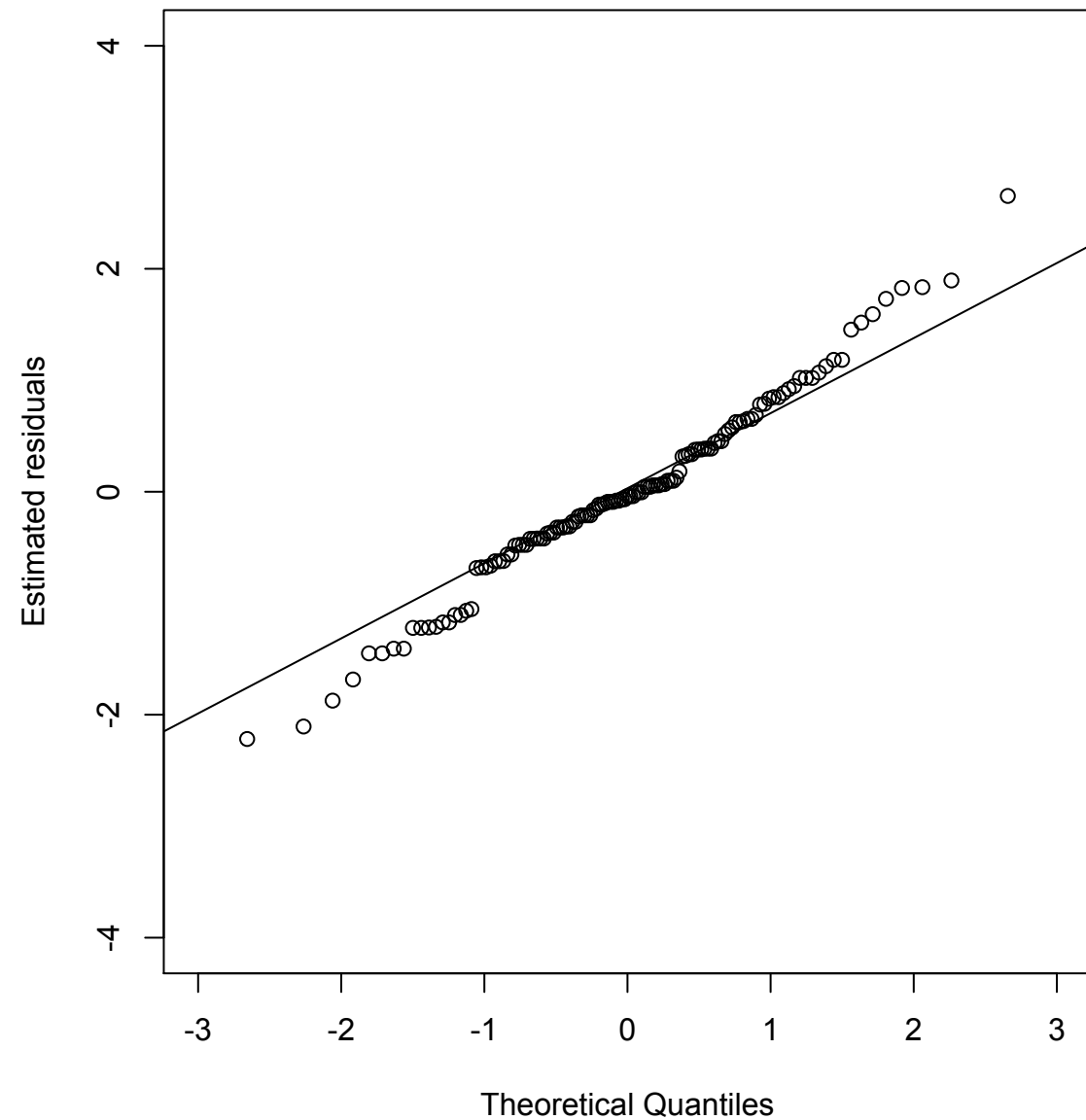

Supplement: S1 File — The code can be executed with the free R software (GNU General Public License). The plots, included in the Results section, can be created directly from the study data with the file PEP_Plots.R. For the Raincloud plots, additional source files are needed from Allen et al. [54]. Further, the ZIP file contains text files with the R software console output, showing the executed code and the results (*.txt file extensions). Lastly, S1 File contains PDF files for all dependent variables with significant predictor variables. The PDF files contain two plots each, showing the QQ-Plots for Random Intercepts and Residuals from the linear mixed-effect model. (ZIP) [file pone.0239553.s003.zip › 14_KSS_S.pdf]

Light Scene=1

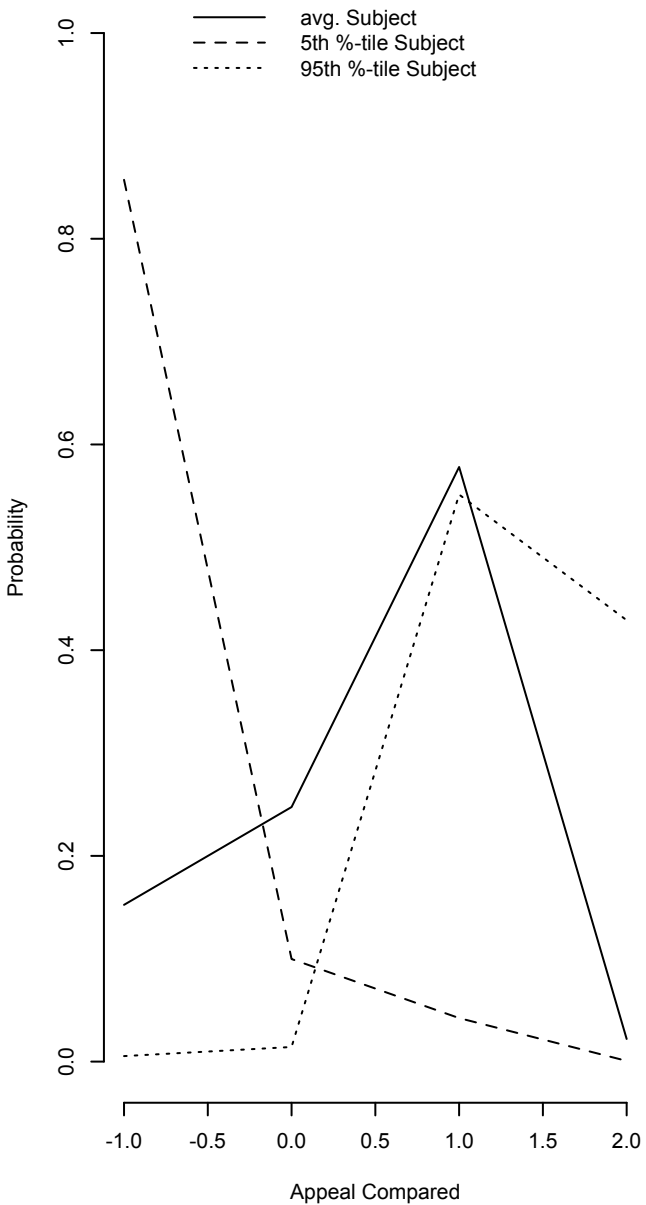

Light Scene=2

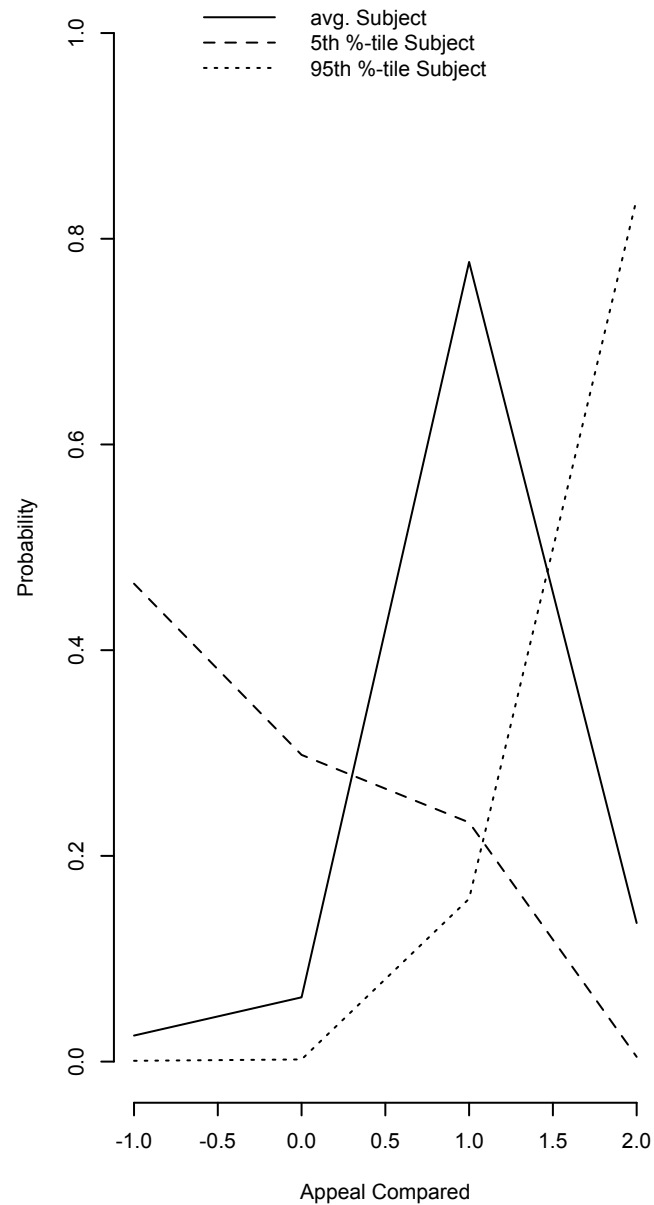

Light Scene=3

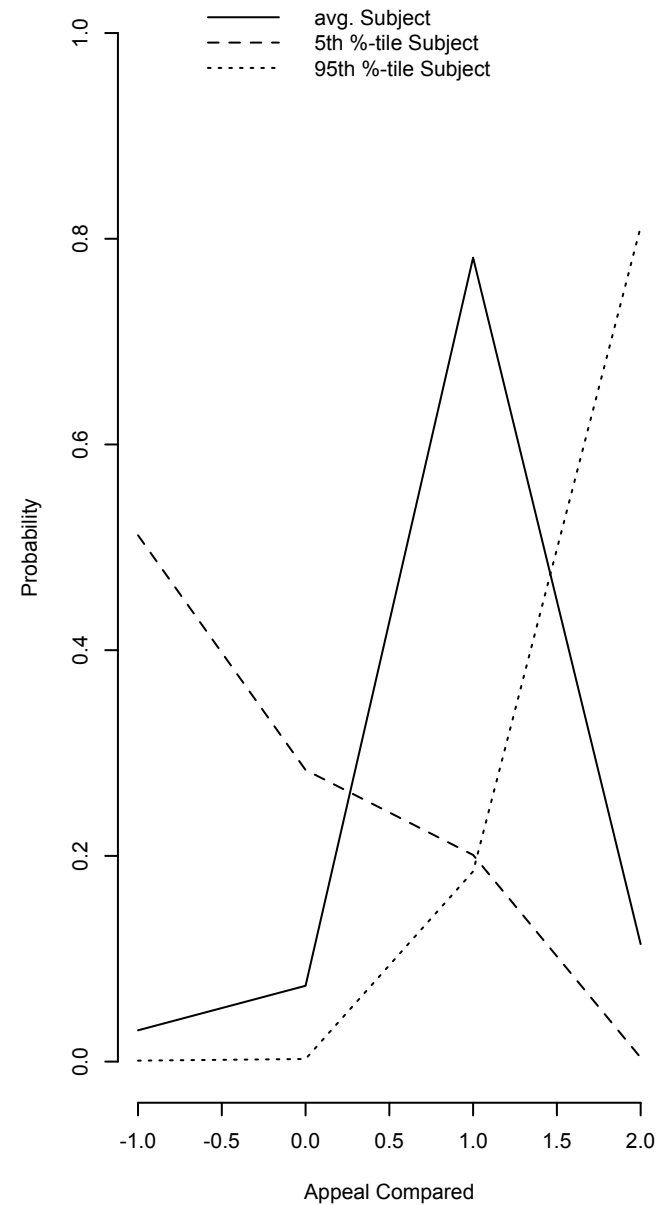

Supplement: S1 File — The code can be executed with the free R software (GNU General Public License). The plots, included in the Results section, can be created directly from the study data with the file PEP_Plots.R. For the Raincloud plots, additional source files are needed from Allen et al. [54]. Further, the ZIP file contains text files with the R software console output, showing the executed code and the results (*.txt file extensions). Lastly, S1 File contains PDF files for all dependent variables with significant predictor variables. The PDF files contain two plots each, showing the QQ-Plots for Random Intercepts and Residuals from the linear mixed-effect model. (ZIP) [file pone.0239553.s003.zip › 16_Appeal-1.pdf]

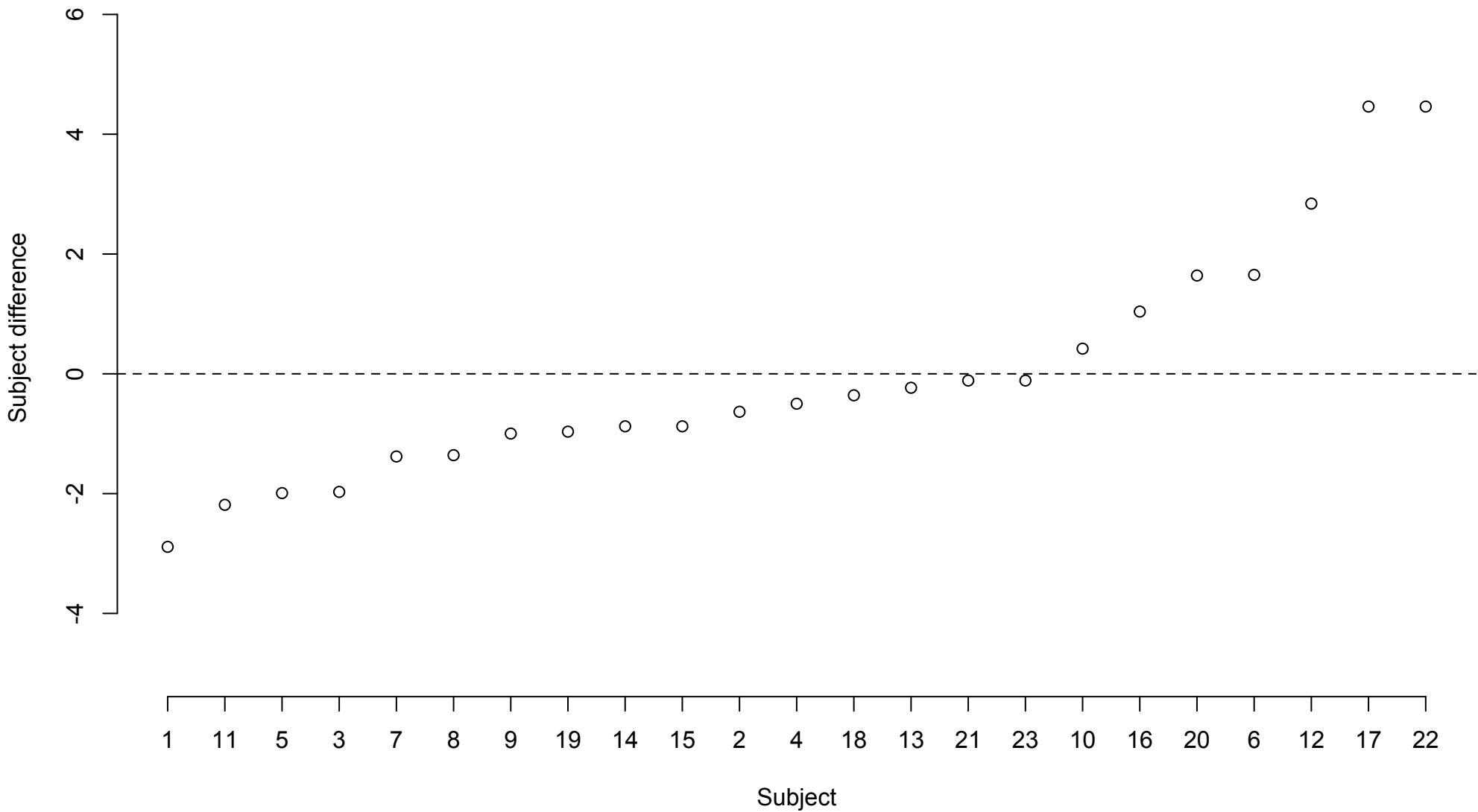

Supplement: S1 File — The code can be executed with the free R software (GNU General Public License). The plots, included in the Results section, can be created directly from the study data with the file PEP_Plots.R. For the Raincloud plots, additional source files are needed from Allen et al. [54]. Further, the ZIP file contains text files with the R software console output, showing the executed code and the results (*.txt file extensions). Lastly, S1 File contains PDF files for all dependent variables with significant predictor variables. The PDF files contain two plots each, showing the QQ-Plots for Random Intercepts and Residuals from the linear mixed-effect model. (ZIP) [file pone.0239553.s003.zip › 16_Appeal.pdf]

Light Scene=1

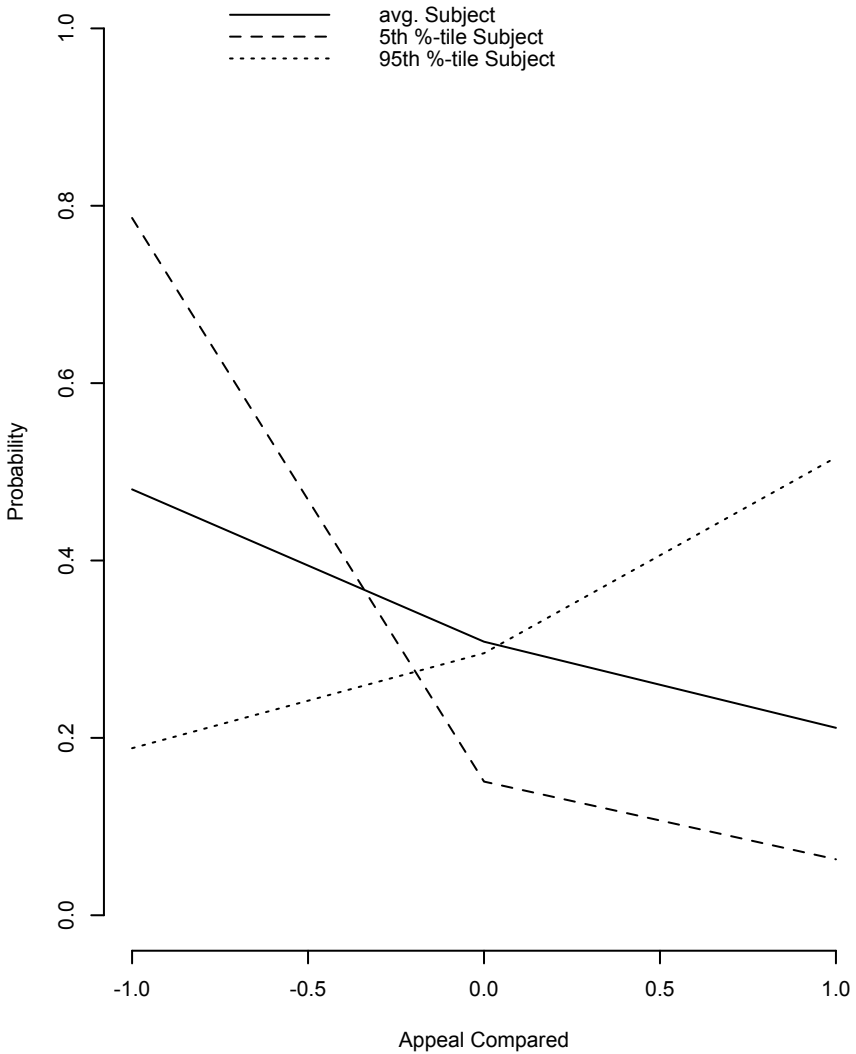

Light Scene=2

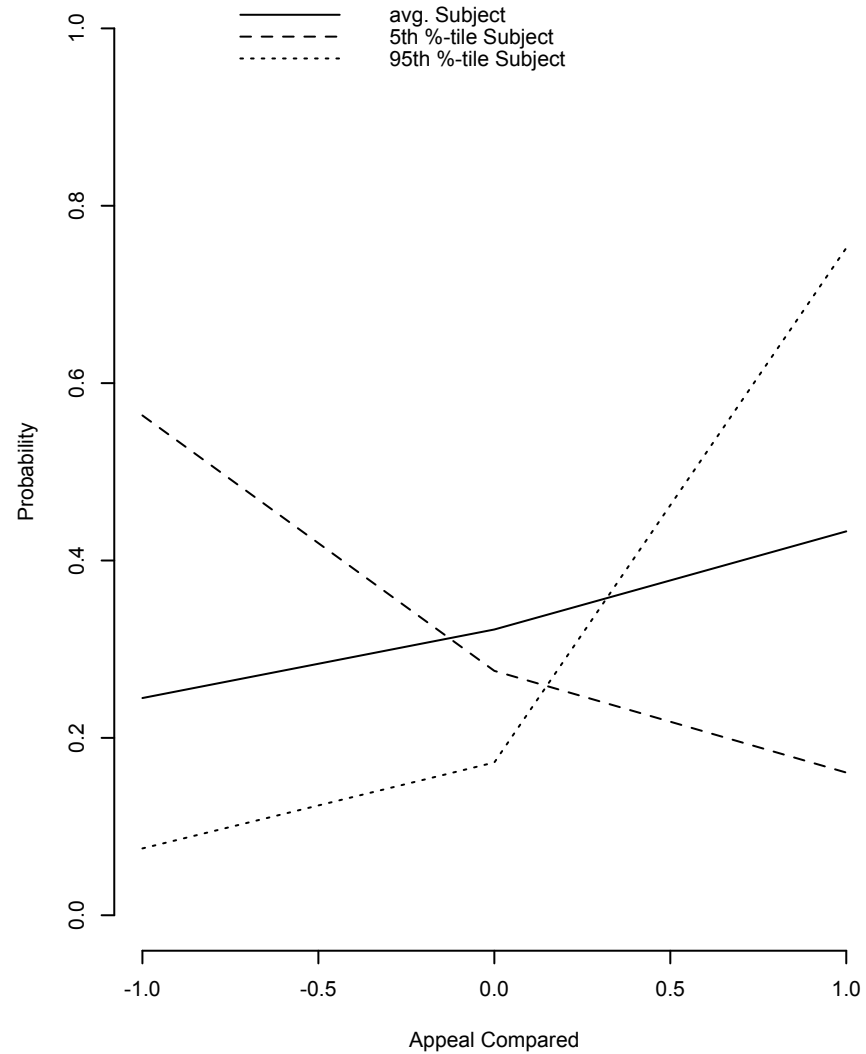

Light Scene=3

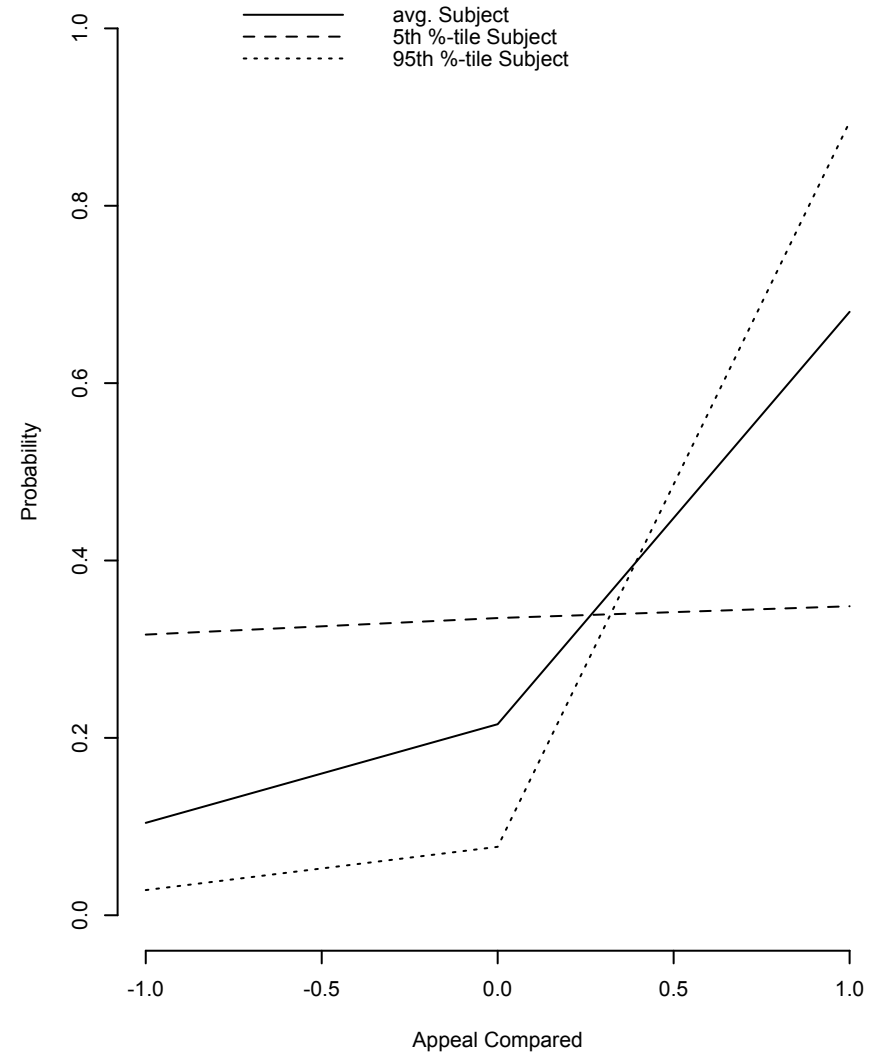

Supplement: S1 File — The code can be executed with the free R software (GNU General Public License). The plots, included in the Results section, can be created directly from the study data with the file PEP_Plots.R. For the Raincloud plots, additional source files are needed from Allen et al. [54]. Further, the ZIP file contains text files with the R software console output, showing the executed code and the results (*.txt file extensions). Lastly, S1 File contains PDF files for all dependent variables with significant predictor variables. The PDF files contain two plots each, showing the QQ-Plots for Random Intercepts and Residuals from the linear mixed-effect model. (ZIP) [file pone.0239553.s003.zip › 17_Appeal_Compared-1.pdf]

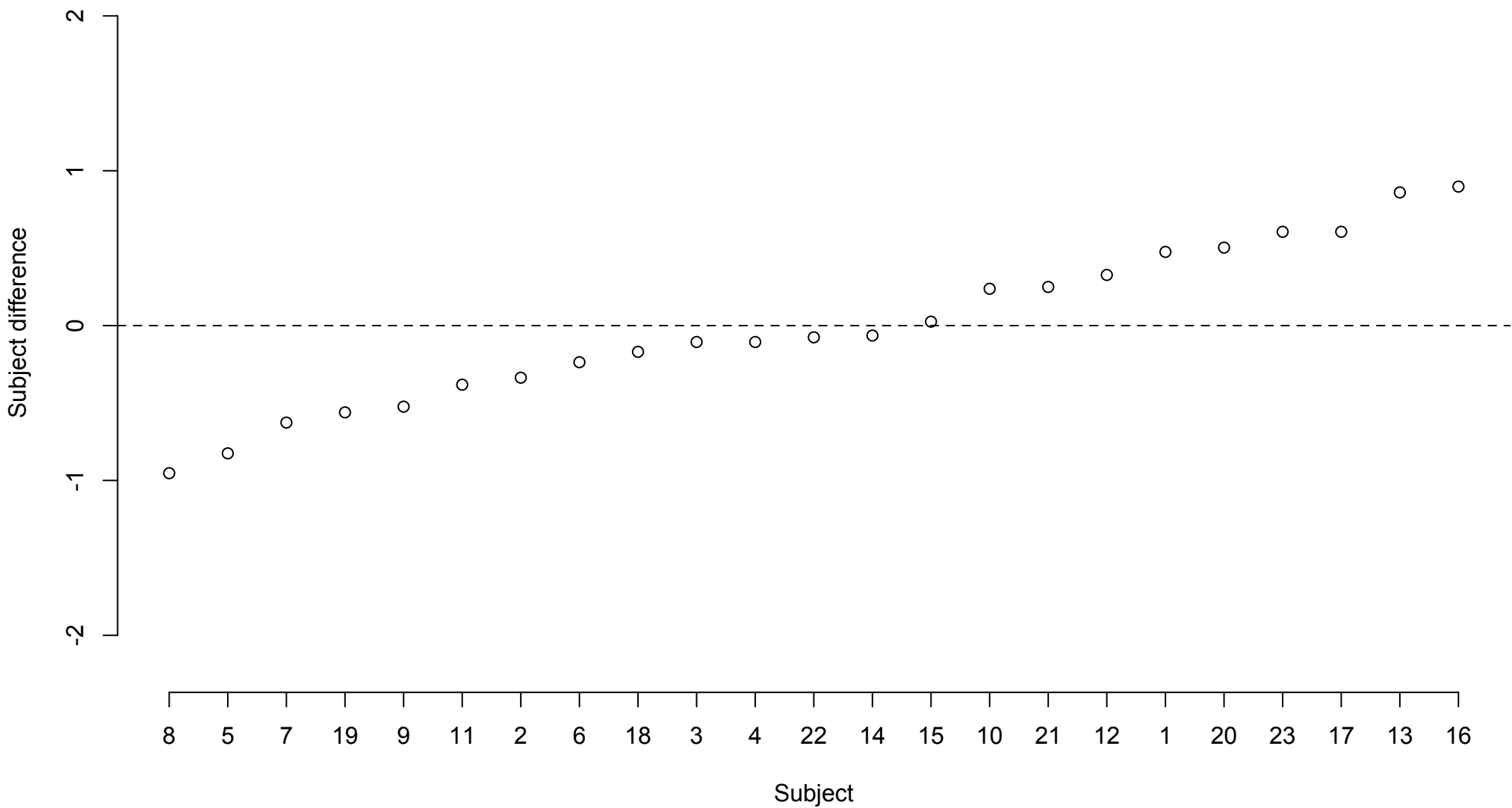

Supplement: S1 File — The code can be executed with the free R software (GNU General Public License). The plots, included in the Results section, can be created directly from the study data with the file PEP_Plots.R. For the Raincloud plots, additional source files are needed from Allen et al. [54]. Further, the ZIP file contains text files with the R software console output, showing the executed code and the results (*.txt file extensions). Lastly, S1 File contains PDF files for all dependent variables with significant predictor variables. The PDF files contain two plots each, showing the QQ-Plots for Random Intercepts and Residuals from the linear mixed-effect model. (ZIP) [file pone.0239553.s003.zip › 17_Appeal_Compared.pdf]

**Random intercepts**

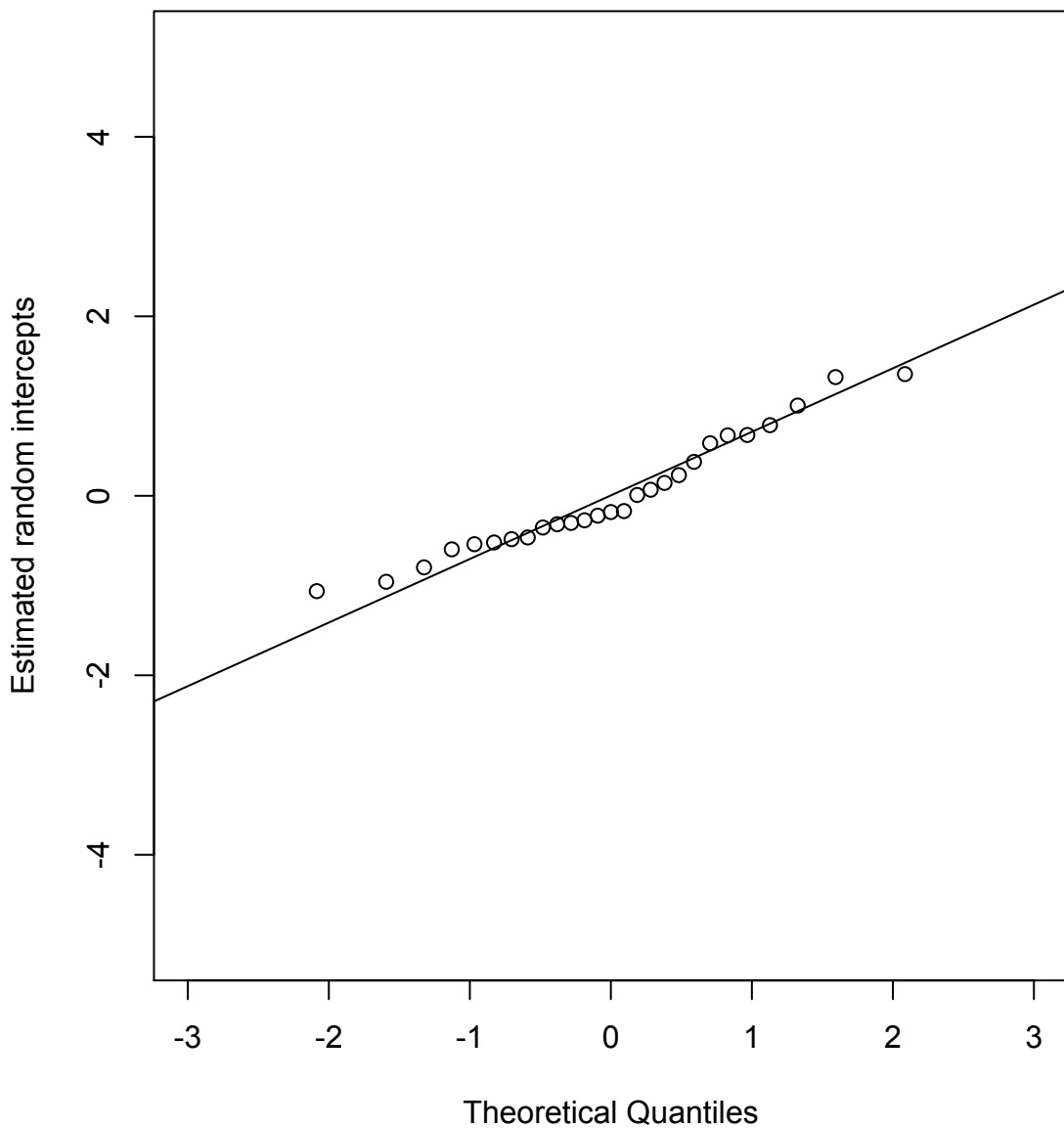

**Residuals**

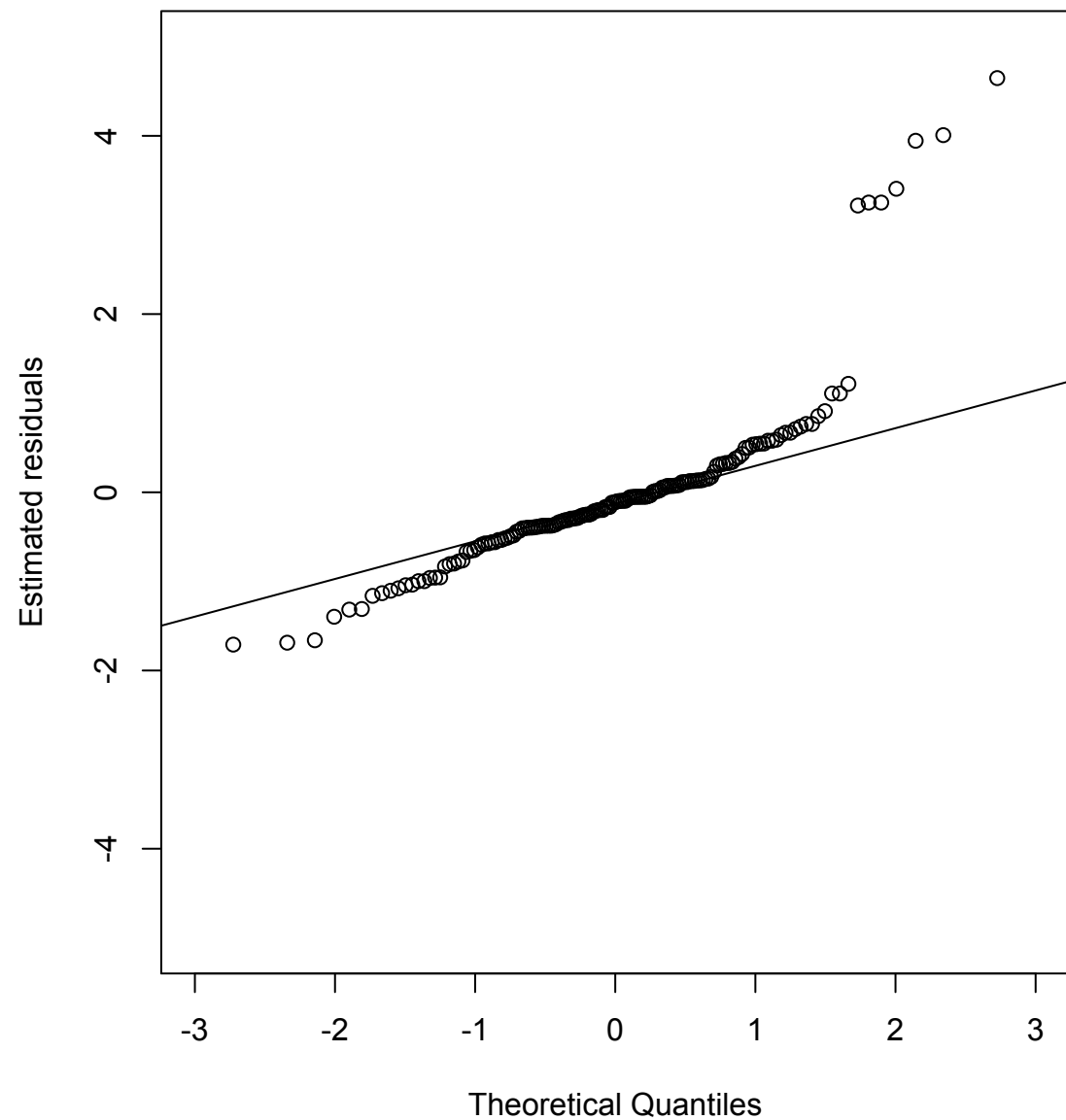

Supplement: S1 File — The code can be executed with the free R software (GNU General Public License). The plots, included in the Results section, can be created directly from the study data with the file PEP_Plots.R. For the Raincloud plots, additional source files are needed from Allen et al. [54]. Further, the ZIP file contains text files with the R software console output, showing the executed code and the results (*.txt file extensions). Lastly, S1 File contains PDF files for all dependent variables with significant predictor variables. The PDF files contain two plots each, showing the QQ-Plots for Random Intercepts and Residuals from the linear mixed-effect model. (ZIP) [file pone.0239553.s003.zip › 19_Mean_Cor.pdf]

**Random intercepts**

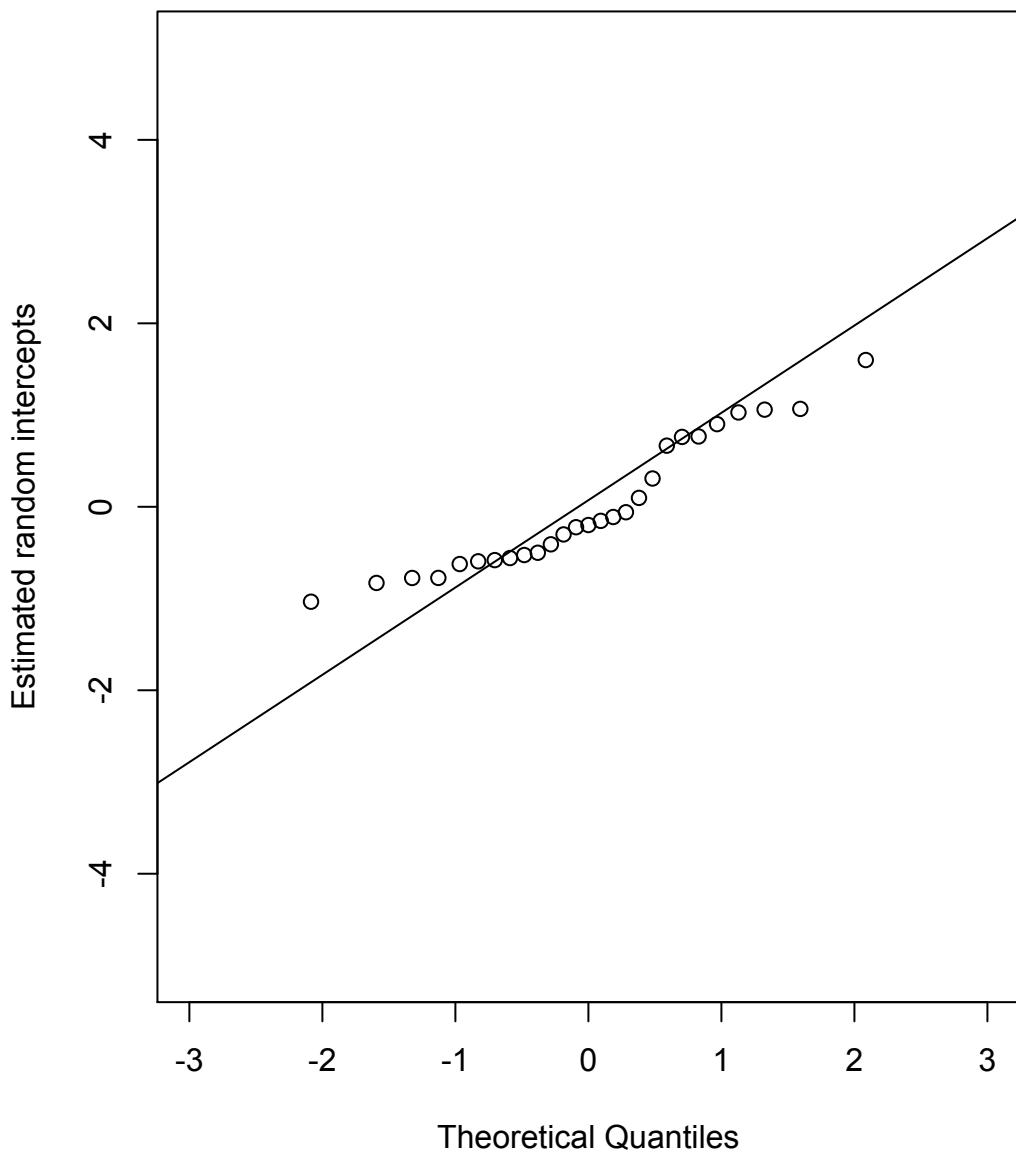

**Residuals**

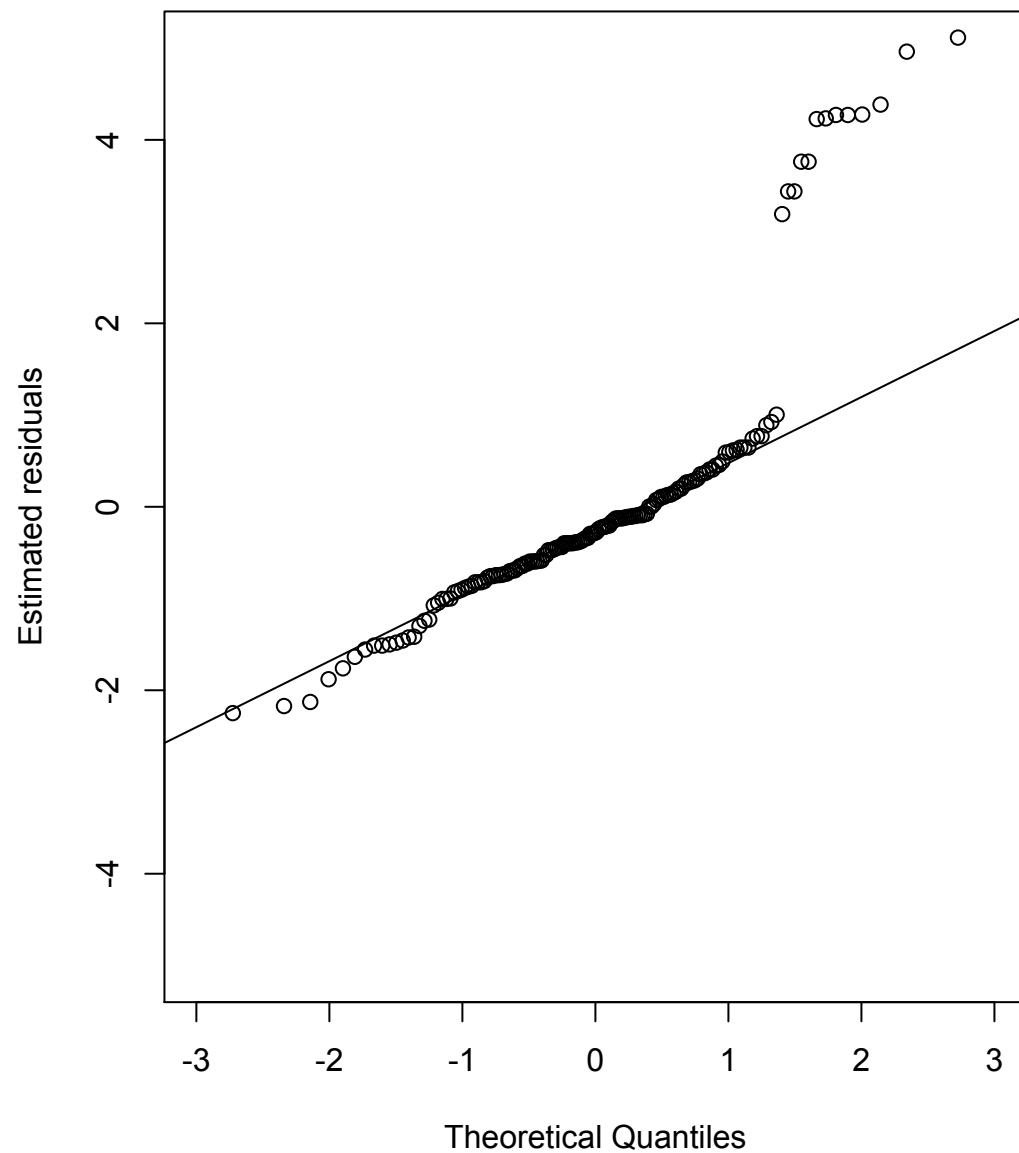

Supplement: S1 File — The code can be executed with the free R software (GNU General Public License). The plots, included in the Results section, can be created directly from the study data with the file PEP_Plots.R. For the Raincloud plots, additional source files are needed from Allen et al. [54]. Further, the ZIP file contains text files with the R software console output, showing the executed code and the results (*.txt file extensions). Lastly, S1 File contains PDF files for all dependent variables with significant predictor variables. The PDF files contain two plots each, showing the QQ-Plots for Random Intercepts and Residuals from the linear mixed-effect model. (ZIP) [file pone.0239553.s003.zip › 20_Mean_Cor_6.pdf]

**Random intercepts**

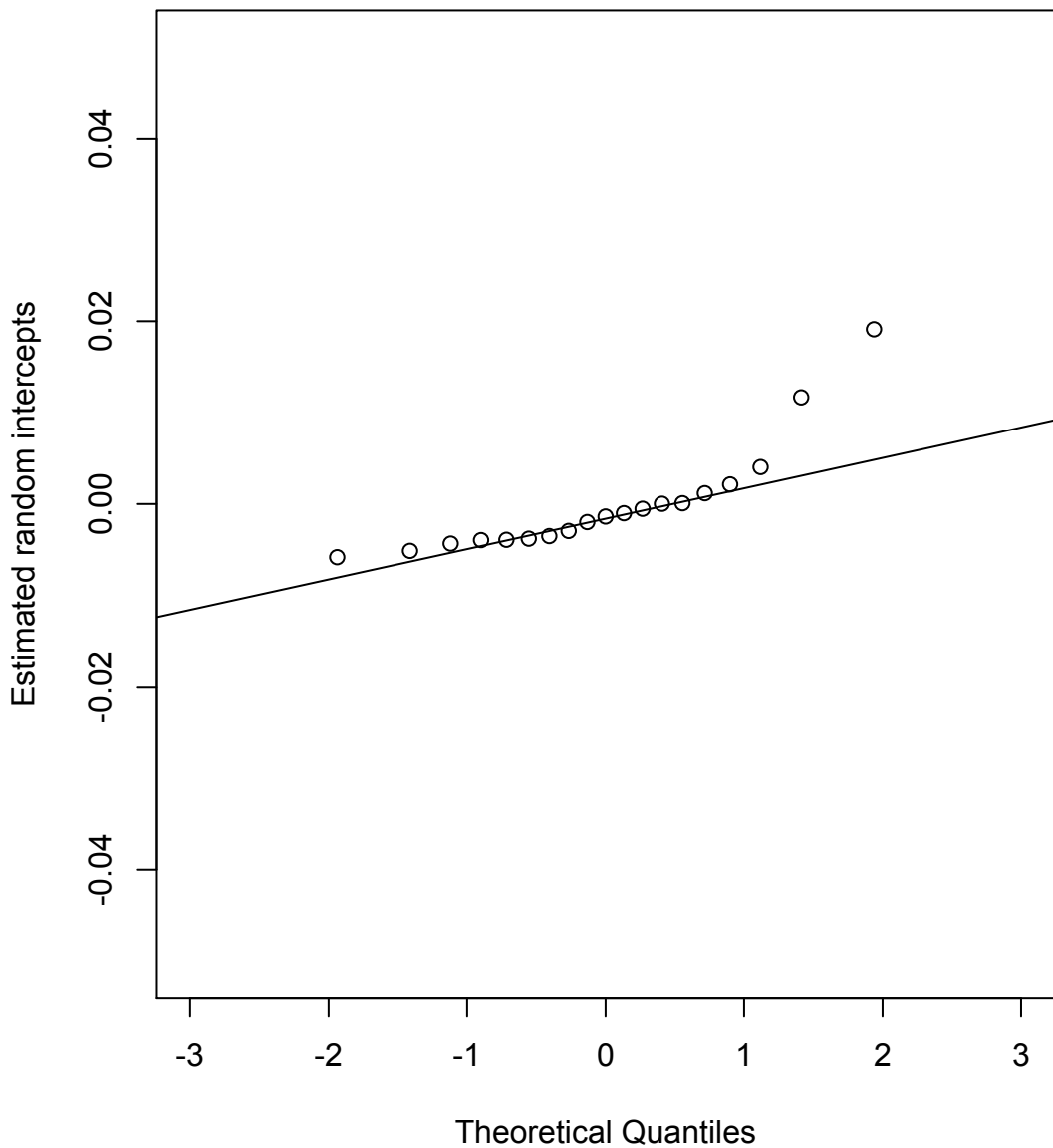

**Residuals**

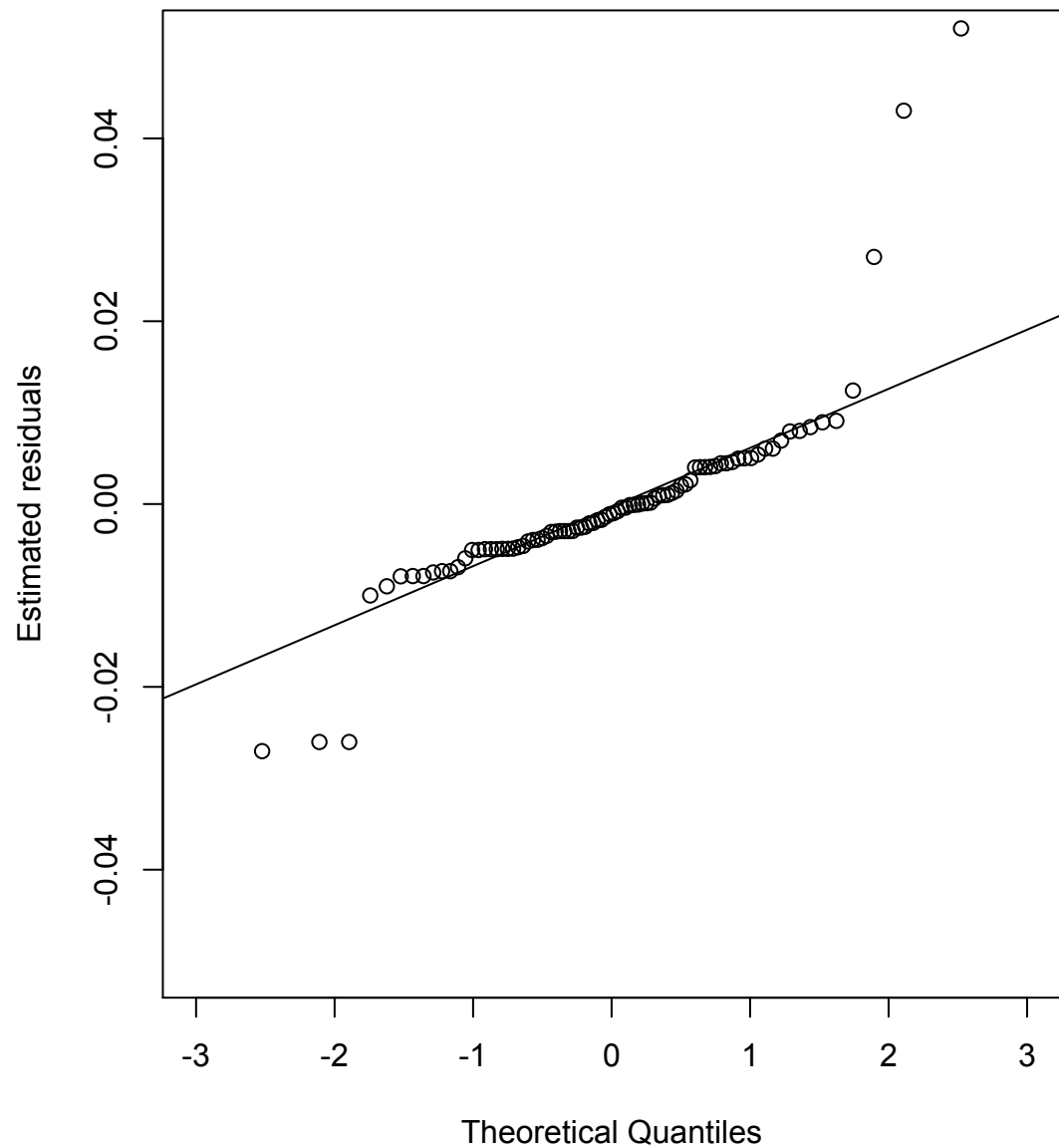

Supplement: S1 File — The code can be executed with the free R software (GNU General Public License). The plots, included in the Results section, can be created directly from the study data with the file PEP_Plots.R. For the Raincloud plots, additional source files are needed from Allen et al. [54]. Further, the ZIP file contains text files with the R software console output, showing the executed code and the results (*.txt file extensions). Lastly, S1 File contains PDF files for all dependent variables with significant predictor variables. The PDF files contain two plots each, showing the QQ-Plots for Random Intercepts and Residuals from the linear mixed-effect model. (ZIP) [file pone.0239553.s003.zip › 23_CO2.pdf]

**Random intercepts**

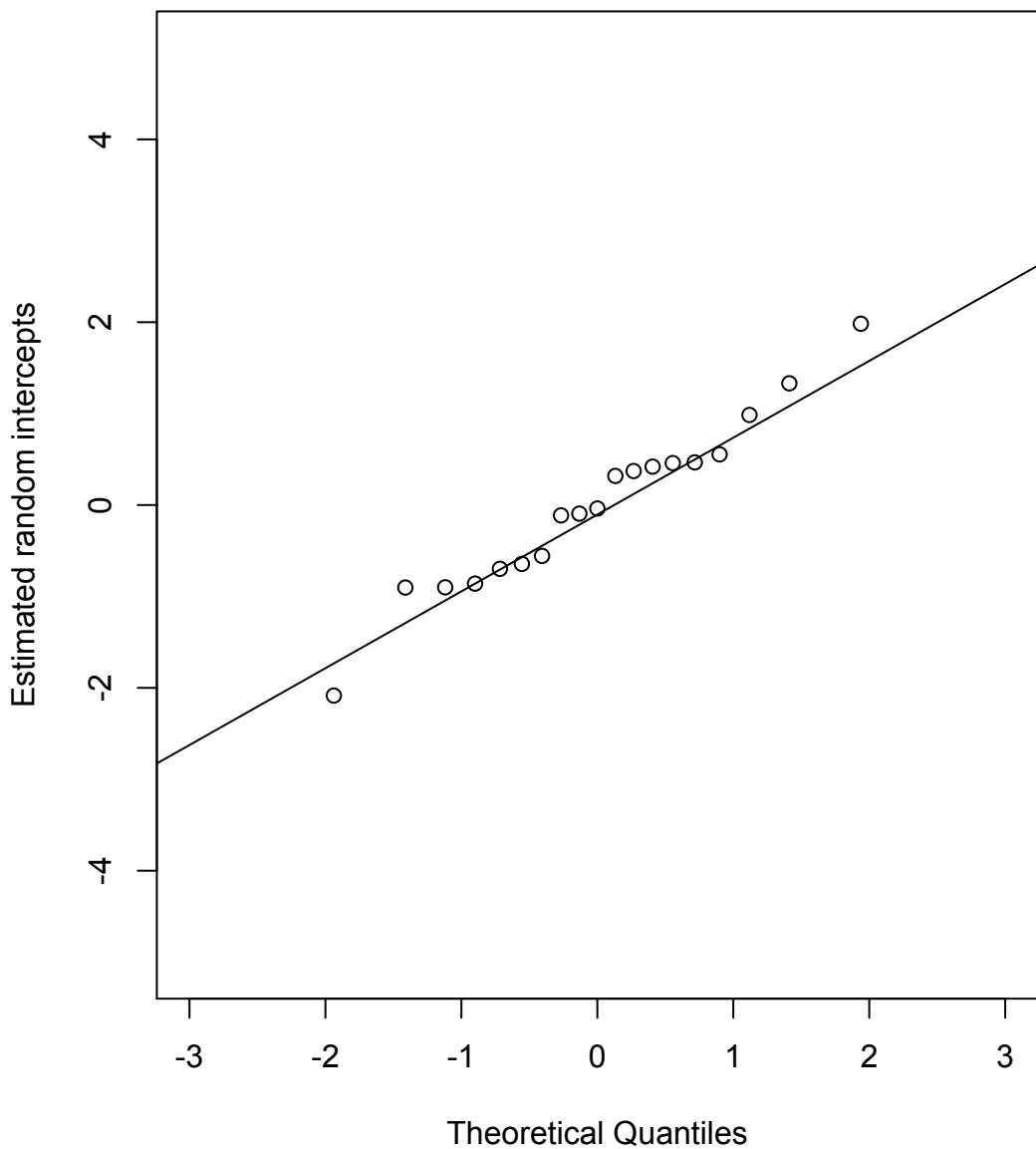

**Residuals**

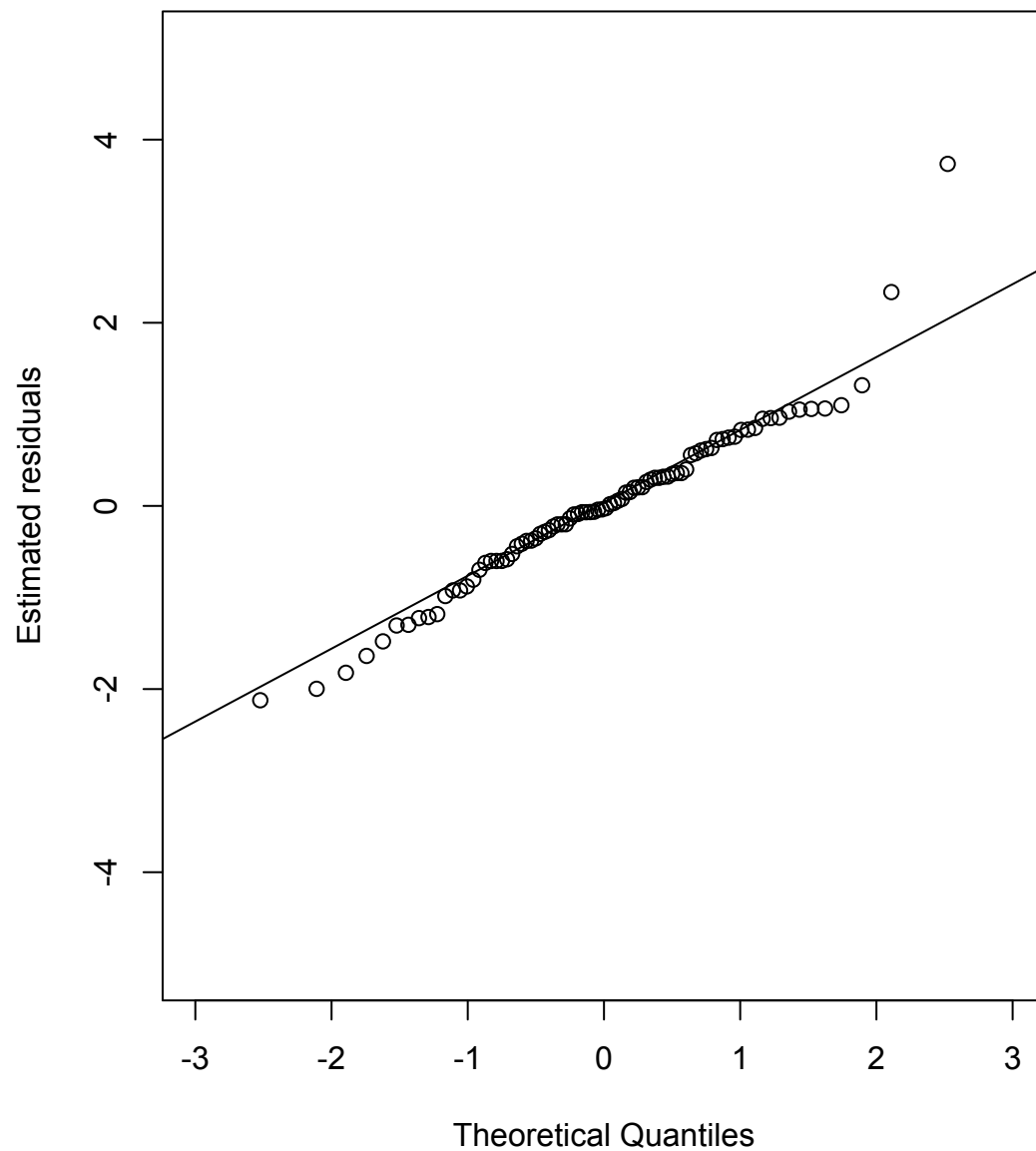

Supplement: S1 File — The code can be executed with the free R software (GNU General Public License). The plots, included in the Results section, can be created directly from the study data with the file PEP_Plots.R. For the Raincloud plots, additional source files are needed from Allen et al. [54]. Further, the ZIP file contains text files with the R software console output, showing the executed code and the results (*.txt file extensions). Lastly, S1 File contains PDF files for all dependent variables with significant predictor variables. The PDF files contain two plots each, showing the QQ-Plots for Random Intercepts and Residuals from the linear mixed-effect model. (ZIP) [file pone.0239553.s003.zip › 24_Temp.pdf]

**Random intercepts**

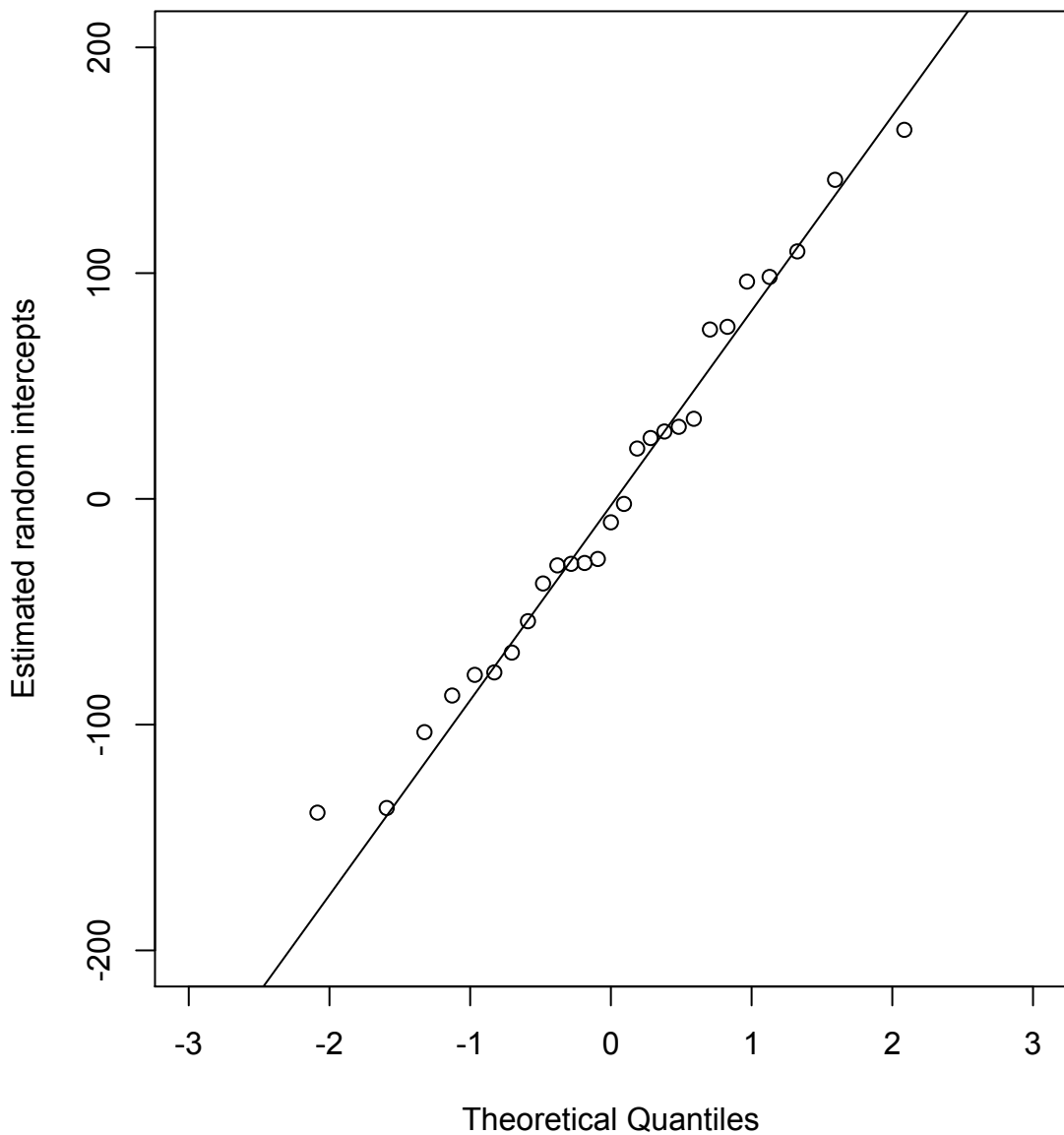

**Residuals**

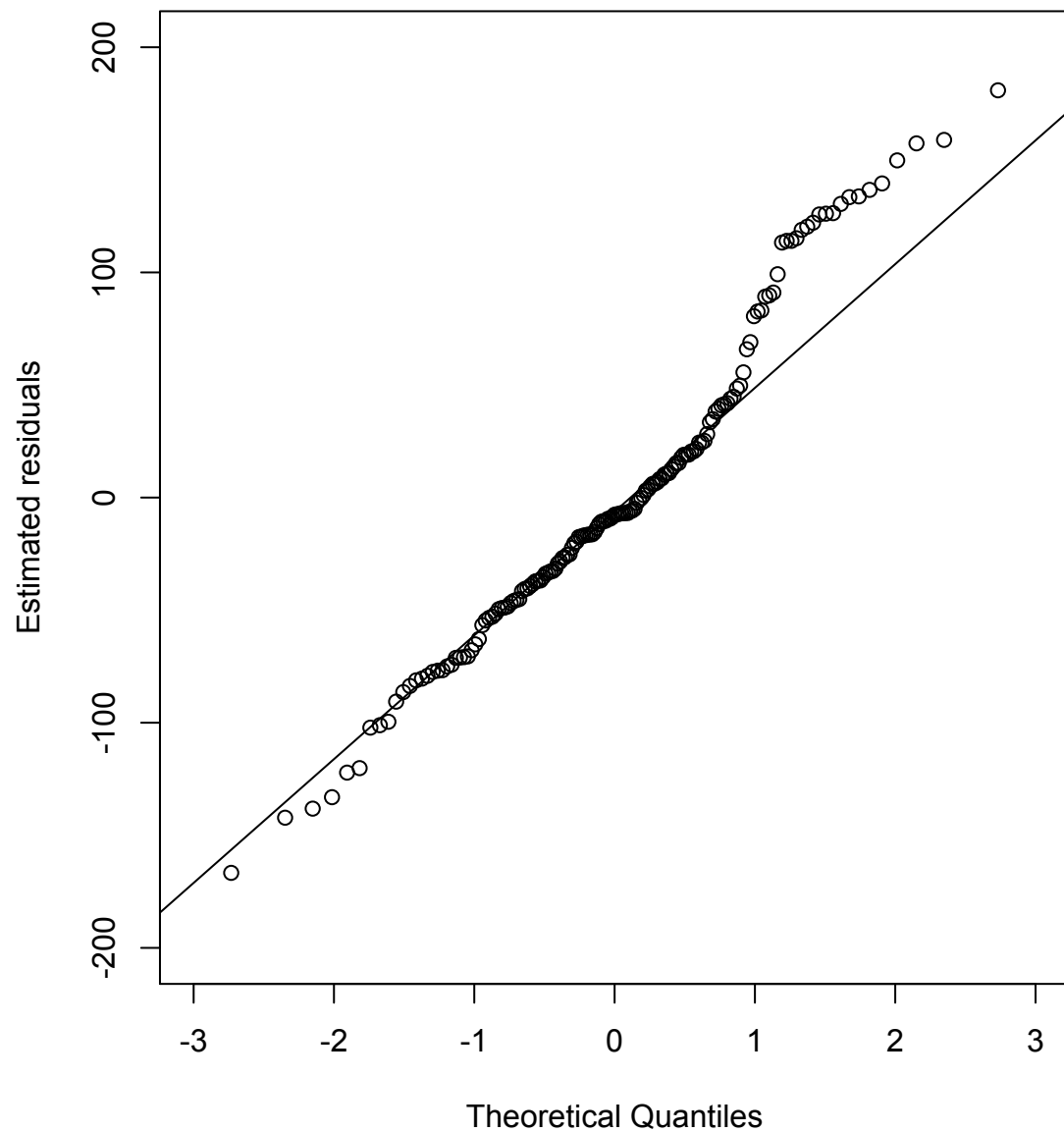

Supplement: S1 File — The code can be executed with the free R software (GNU General Public License). The plots, included in the Results section, can be created directly from the study data with the file PEP_Plots.R. For the Raincloud plots, additional source files are needed from Allen et al. [54]. Further, the ZIP file contains text files with the R software console output, showing the executed code and the results (*.txt file extensions). Lastly, S1 File contains PDF files for all dependent variables with significant predictor variables. The PDF files contain two plots each, showing the QQ-Plots for Random Intercepts and Residuals from the linear mixed-effect model. (ZIP) [file pone.0239553.s003.zip › 28_Mean_RT_2.pdf]

**Random intercepts**

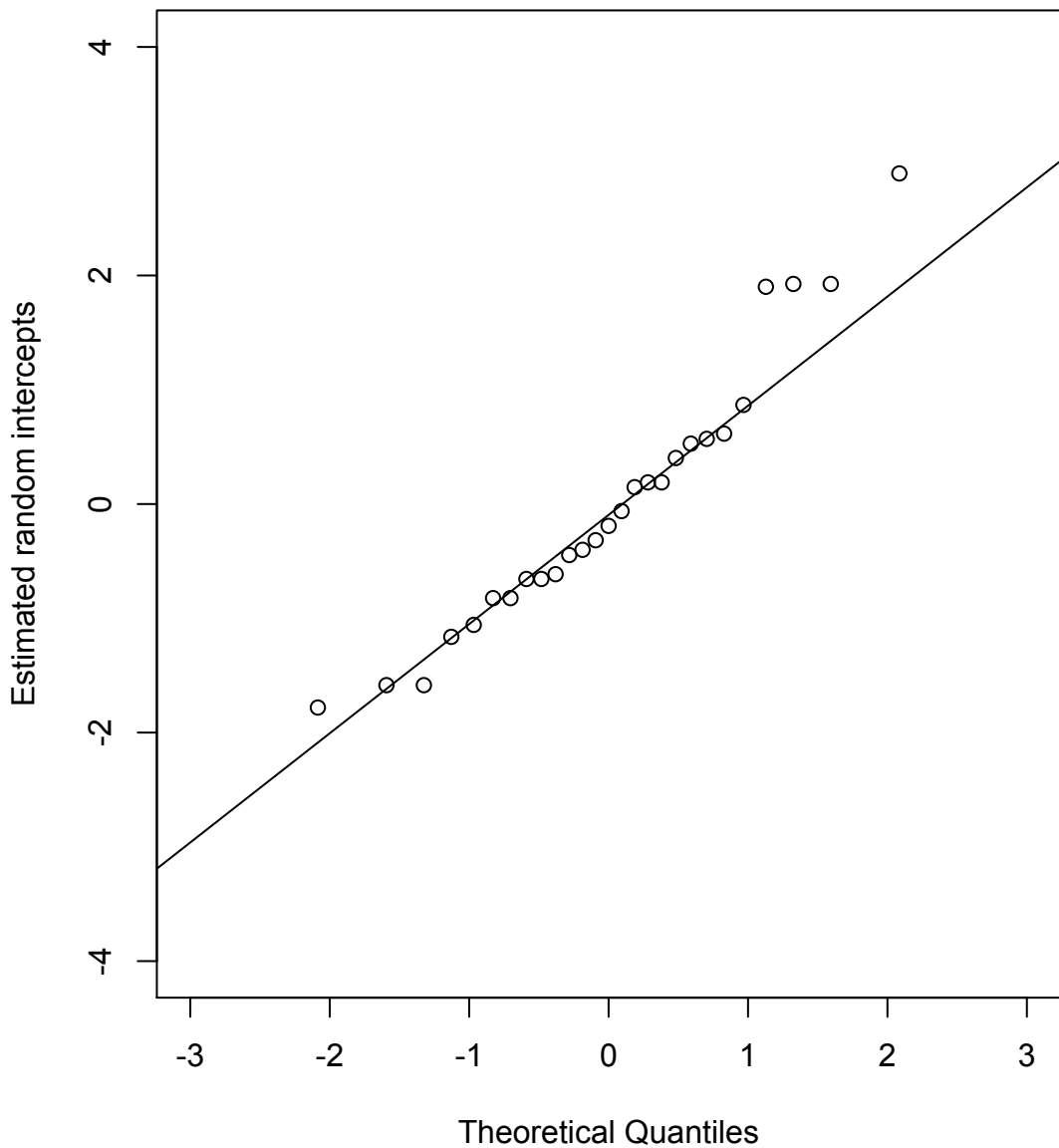

**Residuals**

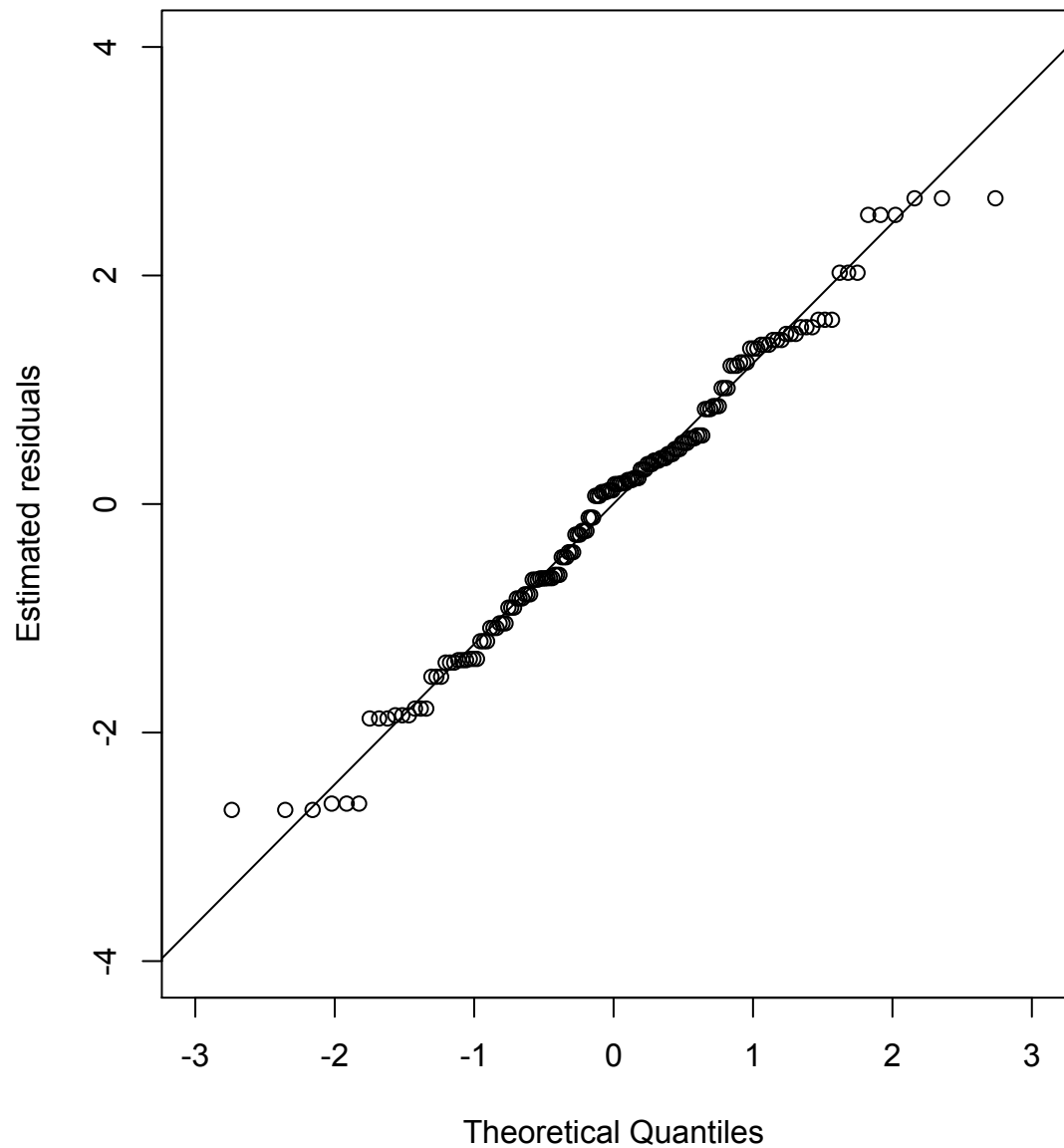

Supplement: S1 File — The code can be executed with the free R software (GNU General Public License). The plots, included in the Results section, can be created directly from the study data with the file PEP_Plots.R. For the Raincloud plots, additional source files are needed from Allen et al. [54]. Further, the ZIP file contains text files with the R software console output, showing the executed code and the results (*.txt file extensions). Lastly, S1 File contains PDF files for all dependent variables with significant predictor variables. The PDF files contain two plots each, showing the QQ-Plots for Random Intercepts and Residuals from the linear mixed-effect model. (ZIP) [file pone.0239553.s003.zip › 33_KSS_D.pdf]
