# Supplementary material for: Influence of common lighting conditions and time-of-day on the effort-related cardiac response
Source: PLoS One. 2020 Oct 7;15(10):e0239553. doi: 10.1371/journal.pone.0239553 (PMC7540875; doi:10.1371/journal.pone.0239553)
Supplement: S2 File — The ZIP file also contains a PDF file with a printout of the executed script for reference. The script was written for the present study, but we encourage its use by other researchers. Test data is provided as S3 File. The script is annotated on a-step-by-step basis, and can be executed with Wolfram Mathematica. In addition to the PDF file provided as part of S2 File, the script can be viewed with the free Wolfram Player. (ZIP) [file pone.0239553.s004.zip › S3_Script_PEP_200331.pdf]

Introduction

This script is part of the Supporting Information material from the article

Influence of common lighting conditions and time-of-day on the effort-related cardiac response

authored by

Johannes Zauner1, Herbert Plischke (1), Hanna Stijnen (2), Ulrich T. Schwarz (2), and Hans Strasburger (3)

(1) Munich University of Applied Sciences, Munich, Germany
(2) Institute of Physics, Chemnitz University of Technology, Chemnitz, Germany
(3) Institute of Medical Psychology, Ludwig-Maximilians-Universität, Munich, Germany

The purpose of this script is to calculate values for heart rate (HR), cardiac left ventricular pre-ejection time (PEP), and cardiac left ventricular ejection time (LVET) from simultaneous measurements of electrocardiogram (ECG) and impedance cardiogram (ICG). This script was written by Ulrich T. Schwarz for the study described in detail in the article above.
The script is free to use by any researcher. We only ask prospective users to make certain they understand the steps taken by the script to calculate HR, PEP and LVET, in order to provide the correct input for calculation. If not, please consult someone who is familiar with Wolfram Mathematica Notebook and the used algorithms.
We also ask researchers to cite the article above, if they employ this script in their work. Finally, if anyone wishes to build upon this script in order to improve or expand the capabilities, or who wants to give feedback about its use, we would like to hear from you:
ulrich.schwarz@physik.tu-chemnitz.de (script author)
zauner@me.com (corresponding author)

How the script works:

The following passage is an excerpt from the article above (section on Data analysis), modified for clarity in the context of the following script.

The pre-ejection-period (PEP) is the time span between the depolarization of the left ventricle (R onset) and opening of the aortic valve (B point). The R onset is the beginning of the Q-wave signal; it indicates the beginning of the depolarization and can be picked up from the ECG signal. As signature for the beginning of the Q wave, we take the minimum of the ECG's second derivative. Its peak indicates the maximum curvature at the transition of the ECG signal into the Q wave. However, as the Q wave is relatively small, other signatures in the ECG signal can be misinterpreted as the R onset in an automated evaluation of the data. In general, first and higher-order derivatives of noisy signals suffer from containing spurious peaks. We therefore restrict the possible occurrences of R onset to a time window after the, easily identifiable, R peak (peak of the QRS complex). Within that window, the R onset is typically seen as a clear negative peak of the ECG signal's second derivative, which can be located reliably and with high precision and thus allows a reliable identification of the Q wave onset. Heart rate (HR) is then calculated from the time difference of subsequent R points.

The time point for the opening of the aortic valve (B point) is derived from the impedance cardiogram (ICG). The impedance Z, and thus the ICG, is sensitive to a variation of blood volume in the thorax. The first derivative, dZ/dt,

corresponds to blood flow. The second derivative d^2Z/dt^2, in turn, corresponds to a change of the blood flow and is thus indicative for the opening of the heart valves. The B point is the onset of the aortic valve's opening, indicated by a negative peak in the third derivative, d^3Z/dt^3. While, compared to ECG, the ICG signal is smooth and devoid of characteristic spikes, its first, second, and third derivative show distinct features. As selection criterion for picking the correct peak of the third derivative, we use the, easily identifiable, peak of the first derivative, dZ/dt. The B point is obtained as the minimum of d^3Z/dt^3 that occurs just before the maximum in dZ/dt. This strategy allows for an automated evaluation of the PEP interval for the large data sets, with few outliers and the required precision.

To calculate the derivatives of the measured signals, we use the Savitzky-Golay filter, as described in Numerical Recipes. This method allows data smoothing, while keeping intact signatures like peaks, and the simultaneous determination of derivatives. Similar to a moving average, a moving section of the data is selected. However, instead of a simple averaging, the algorithm fits a polynomial of given degree to the selected sequence. Then, one point of the fitted polynomial (usually the central point) is taken as value for the smoothed curve. Higher derivatives are taken from the corresponding derivatives of the fitted polynomial at the respective point. The Savitzky-Golay filter is implemented numerically by a list convolution with a kernel. That kernel is calculated in advance for the number of points for the moving fitting, the order of the polynomial, and the order of the derivative. We use a kernel length of 100 points, corresponding to a time interval of 50 ms, and a 3rd-order polynomial for all kernels and for the ICG and ECG signals. The third derivative of the ICG signal is calculated from the first derivative of the ICG signal, which, together with Z, is provided by the Biopac MP36 system (i.e., the system we used to measure ICG/ECG). We ensure that no time lag gets introduced between the ICG and ECG signals and their derivatives by the Savitzky-Golay filter. Thus, PEP and LVET data get extracted from the ICG and ECG measurements in a semi-automated way and with a by-heartbeat resolution. The Mathematica Notebook output is stored in a text file, with every row containing a timestamp, the corresponding length of cardiac PEP, LVET, and HR for each heartbeat.

```
VB1R.acq
0.5 msec/sample
3 channels
Cardiac Output - Z
Ohms
Cardiac Output - dZ/dt
Ohms/sec
ECG (.05 - 35 Hz)
mV
min CH1 CH2 CH3
 1233660 1233660 1233660
0 41.3666 -0.349731 1.4975
8.33333E-06 41.3666 -0.357666 1.4975
1.66667E-05 41.3666 -0.366211 1.49689
2.5E-05 41.3666 -0.374146 1.49658
3.33333E-05 41.3666 -0.38208 1.49567
4.16667E-05 41.3696 -0.389404 1.49506
```

Figure 1 - structure of the *.dat file used for import. The first 11 rows contain metadata and are discarded by the script. Data is structured in the following columns: Time / Impedance / change in Impedance by time / Electrocardiogram
Two sample data files are supplied together with the supplementary information.

1. Import

Import and control

Place the example data set in the same directory as the Mathematica notebook.

The data set “ExampleData_Short.dat” is a 90 second section from the 4 minutes long data set “ExampleData_Long.dat”. The short data set consists of 180.000 = 90 x 2000 lines plus the 11 lines header. The long data set consists of 1.260.391 lines. To read the long data set, change the import command above to data=Import[“ExampleData_Short.dat”].

For those who never worked with Mathematica: to execute a cell (the part of the notebook marked by a bracket on the right side), place the cursor within that cell and press “ctrl+enter” or enter on the number block of your keyboard.

In case if the dynamic content of the cells with “Manipulate” slow down the computer, we suggest to delete the output cell of the respective “Manipulate” when not needed. This is done by clicking on the bracket of the output cell and pushing delete.

```
In[ ]:= SetDirectory[NotebookDirectory[]];
```

```
In[ ]:= data = Import["ExampleData_Short.dat"];
header = data[[1 ;; 11]]
data = Drop[data, 11];
Dimensions[data]
Length[data]
samplingT = header[[2, 1]]/1000 (* sampling time in seconds *)
```

```
Out[ ]= {{1E.acq}, {0.5, msec/sample}, {3, channels}, {Cardiac, Output, −, Z}, {Ohms}, {Cardiac, Output, −, dZ/dt},
  {Ohms/sec}, {ECG, (.05, −, 35, Hz)}, {mV}, {min, CH1, CH2, CH3}, {490 590, 490 590, 490 590}}
```

```
Out[ ]= {180 000, 4}
```

```
Out[ ]= 180 000
```

```
Out[ ]= 0.0005
```

For better readability of the script, we generate four separate arrays containing time, Z, dZ/dt, and EC.

```
In[ ]:= time = data[[All, 1]];
carZ = data[[All, 2]];
cardZdt = data[[All, 3]];
ECG = data[[All, 4]];
```

Graphical display of delta Z, dZ/dt, and EKG

In[]:=
```
istart = 4000;
iend = 16 000;
GraphicsGrid[{{ListPlot[{time, carZ}þ⟦istart;; iend⟧, FrameLabel → {"Time  [min]", "Cardiac Z [Ohm]"},
        Frame → True,  Joined → True,  ImageSize → Medium]},
    {ListPlot[{time, cardZdt}þ⟦istart;; iend⟧, FrameLabel → {"Time  [min]", "Cardiac dZ/dt [Ohm/s]"},
        PlotRange → All,  Frame → True,  Joined → True,  ImageSize → Medium]},
    {ListPlot[{time, ECG}þ⟦istart;; iend⟧, FrameLabel → {"Time  [min]", "ECG [meV]"},
        PlotRange → All,  Frame → True,  Joined → True,  ImageSize → Medium]}}]
```

Out[]=

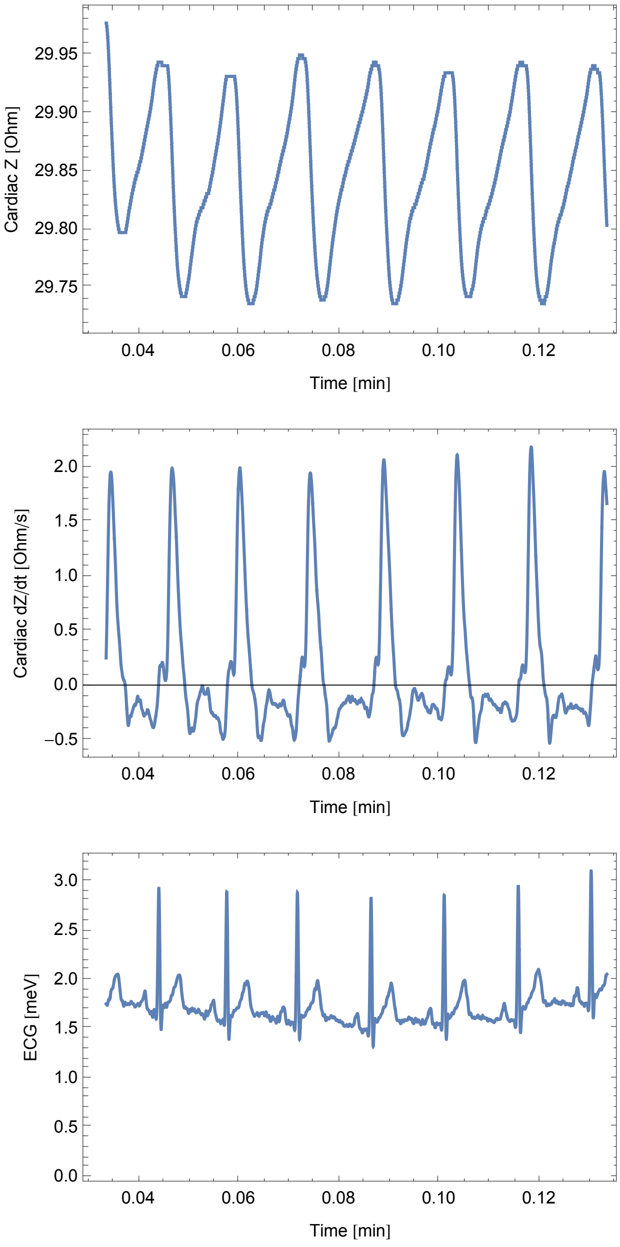

The following cell works only with Mathematica installed (allowing to dynamically scroll through the data set).

Let’s have a look at the raw Cardiac Z, dZ/dt and ECG data. Within “Manipulate” you can scroll through the

data by moving the ruler or (after clicking on the “+“ to the side of the ruler” using the additional buttons.
length = 12000; defines the length of the window, here 12.000 samples = 6 seconds.
The sample data has the typical motion artefacts from a real experiment.

```
In[ ]:= length =12 000;
Manipulate[
  GraphicsGrid[{{ListPlot[{time, carZ}þ[[i;; i +length]], FrameLabel →{"Time  [min]","Cardiac Z [Ohm]"},
          Frame→True,  Joined→True,  ImageSize→Medium]},
      {ListPlot[{time, cardZdt}þ[[i;; i +length]], FrameLabel →{"Time  [min]","Cardiac dZ/dt [Ohm/s]"},
          PlotRange→All,  Frame→True,  Joined→True,  ImageSize→Medium]},
      {ListPlot[{time, ECG}þ[[i;; i +length]], FrameLabel →{"Time  [min]","ECG [meV]"},
          PlotRange→All,  Frame→True,  Joined→True,  ImageSize→Medium]}}],
  {i, 1, Length[time]−length, length}, ControlPlacement→Top]
```

2. Using the Savitzky-Golay Kernel to derivate experimental data

Applying the Savitzky-Golay filter to a data set involves two steps. First, the filter kernel is generated using the SavitzkyGolayMatrix[{kernellength},3,2]; will generate a filter kernel of length kernellength (here 100 points), the polynomial order is given by the second argument (here 3), and the order of the derivative (here 2nd derivative) as third parameter. Second, a convolution of the kernel with the data set. To keep the data aligned in time, the third parameter in ListConvolve defines the correct padding. Furthermore, for the second derivative the result is divided by the squared time difference between to time steps to get the correct absolute amplitude of the derivative.
Depending on the length of the data set and CPU speed, the convolution may take a few seconds.

Kernel for the third derivative cardiac Z (2. derivative of the measured 1. derivative dZ/dt)

```
In[ ]:= kernellength=100;
derivativekernel =SavitzkyGolayMatrix[{kernellength}, 3, 2];
```

```
In[ ]:= card3Zdt3 =ListConvolve[derivativekernel, cardZdt, kernellength]/samplingT^2;
```

First and second derivative of the ECG Signal

```
In[ ]:= kernellength=100;
```

```
In[ ]:= derivativekernel =SavitzkyGolayMatrix[{kernellength}, 3, 1];
```

```
In[ ]:= dECGdt =ListConvolve[derivativekernel, ECG, kernellength]/samplingT;
```

```
In[ ]:= derivativekernel =SavitzkyGolayMatrix[{kernellength}, 3, 2];
```

```
In[ ]:= d2ECGdt2 =ListConvolve[derivativekernel, ECG, kernellength]/samplingT^2;
```

Test: Displaying the Data together with the calculated derivatives

In[ ]:=
```
GraphicsGrid[{
    {ListPlot[{time, carZ}þ[[istart;; iend]], FrameLabel →{"Time  [min]", "Cardiac Z [Ohm]"},
        Frame→True,  Joined→True, ImageSize→Medium],
      ListPlot[{time, cardZdt}þ[[istart;; iend]], FrameLabel →{"Time  [min]", "Cardiac dZ/dt [Ohm/s]"},
        PlotRange→All, Frame→True,  Joined→True, ImageSize→Medium],
      ListPlot[{time, card3Zdt3}þ[[istart ;; iend]], FrameLabel →{"Time  [min]", "Cardiac d^3Z/dt^3 [Ohm/s^2]"},
        PlotRange→All, Frame→True,  Joined→True, ImageSize→Medium]},
    {ListPlot[{time, ECG}þ[[istart;; iend]], FrameLabel →{"Time  [min]", "ECG [mV]"},
        Frame→True,  Joined→True, PlotRange→All, ImageSize→Medium],
      ListPlot[{time, dECGdt}þ[[istart;; iend]], FrameLabel →{"Time  [min]", "dECG/dt [mV/s]"},
        PlotRange→All, Frame→True,  Joined→True, PlotRange→All, ImageSize→Medium],
      ListPlot[{time, d2ECGdt2}þ[[istart ;; iend]], FrameLabel →{"Time  [min]", "d^2ECG/dt^2 [mV/s^2]"},
        PlotRange→All, Frame→True,  Joined→True, PlotRange→All, ImageSize→Medium]}}
]
```

Out[ ]=

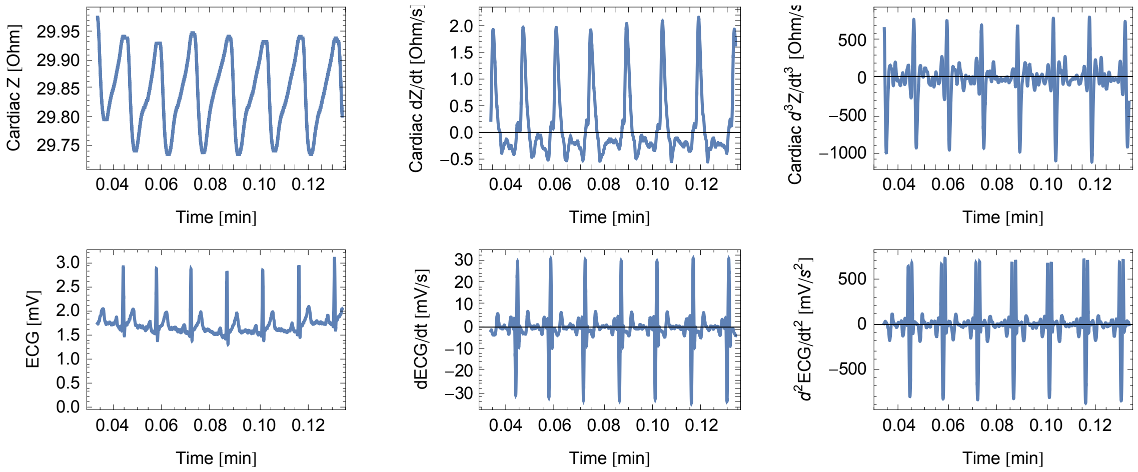

The following cell works only with Mathematica installed:

```
In[-]:= length =6000;
Manipulate[
  GraphicsGrid[{
     {ListPlot[{time, carZ}þ[[i;; i +length]], FrameLabel →{"Time  [min]","Cardiac Z [Ohm]"},
        Frame→True,  Joined→True, ImageSize→Medium],
      ListPlot[{time, cardZdt}þ[[i;; i +length]], FrameLabel →{"Time  [min]","Cardiac dZ/dt [Ohm/s]"},
        PlotRange→All, Frame→True,  Joined→True, ImageSize→Medium],
      ListPlot[{time, card3Zdt3}þ[[i;; i +length]],
        FrameLabel →{"Time  [min]","Cardiac d³Z/dt³ [Ohm/s²]"},
        PlotRange→All, Frame→True,  Joined→True, ImageSize→Medium]},
     {ListPlot[{time, ECG}þ[[i;; i +length]], FrameLabel →{"Time  [min]","ECG [mV]"},
        Frame→True,  Joined→True, PlotRange→All, ImageSize→Medium],
      ListPlot[{time, dECGdt}þ[[i;; i +length]], FrameLabel →{"Time  [min]","dECG/dt [mV/s]"},
        PlotRange→All, Frame→True,  Joined→True, PlotRange→All, ImageSize→Medium],
      ListPlot[{time, d2ECGdt2}þ[[i;; i +length]], FrameLabel →{"Time  [min]","d²ECG/dt² [mV/s²]"},
        PlotRange→All, Frame→True,  Joined→True, PlotRange→All, ImageSize→Medium]}}
  ],
  {i, 1, Length[time]−length, length}, ControlPlacement→Top]
```

3. Maxima and Minima

Next, we search for specific maxima and minima in the curves, as described in the algorithm. “FindPeaks[dataset,...]” is a comfortable routine to search for peaks. We use FindPeaks[dataset, σ, s, t] with three parameters in addition to the data set provided. The second parameter defines a $\sigma$for Gaussian blurring before peak search, the second parameter defines a minimum sharpness, and the third a minimum amplitude. For details see Mathematica help.

The user has to check if peaks (max and min) are found correctly - they are marked as colored dots in the diagrams following their calculation. If not, the appropriate value in the FindPeaks command (last of four parameters) needs to be adjusted. First try to set the correct height (last parameter).

Minima for 2nd derivative ECG

First find negative peaks of the second derivative of the ECG signal.
Usually the first or last point has do be dropped, in order to find the corresponding points in the other signals. The first and last point are dropped (otherwise error messages “Part -1 of {} does not exist” will be generated below, when algorithms search beyond the length of the data set).

```
In[-]:= Minimad2ECGdt2 =Drop[Drop[Round[FindPeaks[−d2ECGdt2, 0, 0, 500][[All, 1]]], −1],1];
```

In[ ]:=
```
imin =4000; ( * define window of displayed data in the plots below * )
imax =14 000;
ListLinePlot[
  {time⟦imin ;; imax⟧, d2ECGdt2⟦imin ;; imax⟧}þ,
  PlotRange →All,  AxesLabel →{"time  [min]","d^2ECG/dt^2 [mV/s^2]"},
  Epilog →{Blue,   PointSize[0.01], Point[{time⟦Minimad2ECGdt2⟧, d2ECGdt2⟦Minimad2ECGdt2⟧}þ]}
]
```

Out[ ]=

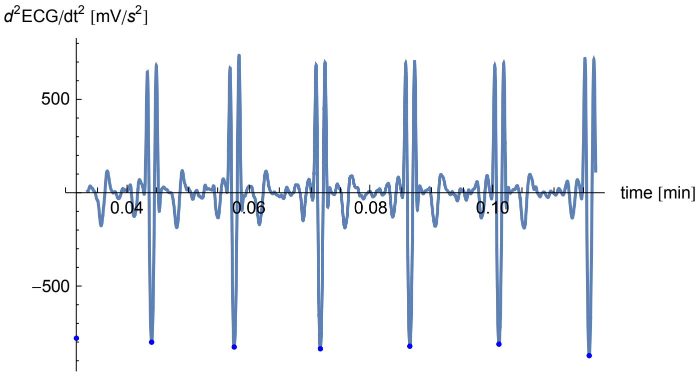

Minima ECG for R-onset

Then search for the last minimum in the ECG signal (red point) before the maximum of the second derivative of the ECG signal (blue point). This is done in two steps: first search for all minima (setting the third parameter in FindPeaks to -∞), then select the closest minimum of the ECG signal before each maximum of the second derivative.

In[ ]:=
```
peaks  =Round[FindPeaks[−ECG,  0, 0, −∞]⟦All,1⟧];
RonsetIndex =Table[Select[peaks, #<Minimad2ECGdt2⟦i⟧&]⟦−1⟧,  {i, 1, Length[Minimad2ECGdt2]}];
```

In[ ]:=
```
ListLinePlot[
  {time⟦imin ;; imax⟧, ECG⟦imin ;; imax⟧}þ, PlotRange →All,  AxesLabel →{"time  [min]","ECG [mV]"},
  Epilog →{Red,   PointSize[0.01], Point[{time⟦RonsetIndex⟧, ECG⟦RonsetIndex⟧}þ],
      Blue, Point[{time⟦Minimad2ECGdt2⟧, ECG⟦Minimad2ECGdt2⟧}þ]}
]
```

Out[ ]=

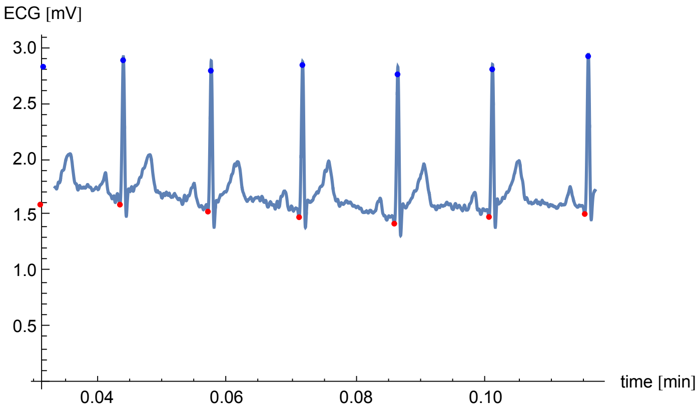

Maxima 1st derivative cardZdt

Find the maxima of the 1st derivative of cardiac dZ/dt, which is needed to find the B-point in the next step. In principle, the first line is enough, but then because of artefacts, some additional points will show up. Therefore we select for each minimum in the second derivative of the ECG the first one following a maximum of cardiac dZ/dt. In the second line one extra point is appended to take care of the end of the data set.

```
In[]:= MaxcardZdt =Round[FindPeaks[cardZdt, 0, 0, 1]]⟦All,1⟧;
AppendTo[MaxcardZdt,Minimad2ECGdt2⟦-1⟧+10];
MaxcardZdt =Table[Select[MaxcardZdt,#>Minimad2ECGdt2⟦i⟧&]⟦1⟧, {i, Length[Minimad2ECGdt2]}];
```

```
In[]:= ListLinePlot[
  {time⟦imin ;; imax⟧, cardZdt⟦imin ;; imax⟧}ᵀ,
  PlotRange →All, AxesLabel →{"time [min]","dZ/dt [Ohm/s]"},
  Epilog →{Blue, PointSize[0.01],Point[{time⟦MaxcardZdt⟧, cardZdt⟦MaxcardZdt⟧}ᵀ]}
]
```

Out[]=

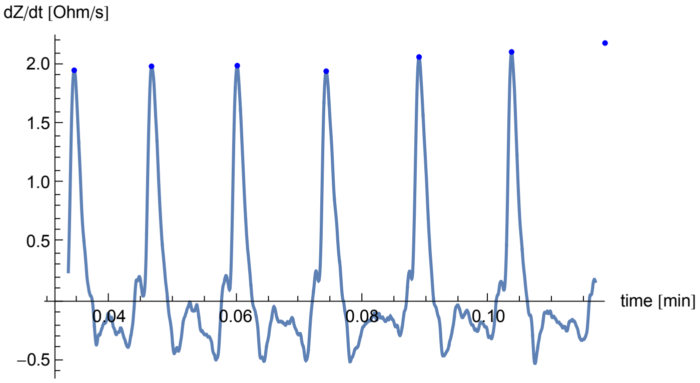

Maxima 3rd derivative carZ for B-Point

The maxima of the third derivative of cardiac Z which is just before the maximum of dZ/dt correspond the the B-point (green points).
The third derivative has many peaks of similar height. So in a first steps all peaks of a significant height are selected (e.g. with an amplitude larger than 200), and from these the last peak before each maximum of dZ/dt (blue points from above) is selected.

```
In[]:= Maxcard3Zdt3 =Round[FindPeaks[card3Zdt3, 2, 0, 200]]⟦All,1⟧;
BpunktIndex =Table[Select[Maxcard3Zdt3,#<(MaxcardZdt⟦i⟧)&]⟦-1⟧, {i, Length[MaxcardZdt]}];
```

In[-]:=
```
ListLinePlot[
  {time⟦imin ;; imax⟧, card3Zdt3⟦imin ;; imax⟧}þ,
  PlotRange →All, AxesLabel →{"time [min]","d³Z/dt³ [Ohm/s³]"},
  Epilog →{Green, PointSize[0.01],Point[{time⟦BpunktIndex⟧, card3Zdt3⟦BpunktIndex⟧}þ],
      Blue, Point[{time⟦MaxcardZdt⟧, card3Zdt3⟦MaxcardZdt⟧}þ]}
]
```

Out[-]=

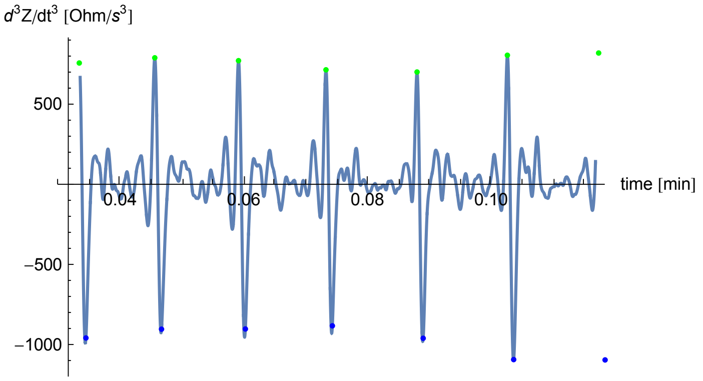

Below, the same points are shown in the cardiac dZ/dt data set. It is difficult to see that the B-point (green) is at the maximum curvature of the dZ/dt curve.

In[-]:=
```
ListLinePlot[
  {time⟦imin ;; imax⟧, cardZdt⟦imin ;; imax⟧}þ,
  PlotRange →All, AxesLabel →{"time [min]","dZ/dt [Ohm/s]"},
  Epilog →{Green, PointSize[0.01],Point[{time⟦BpunktIndex⟧, cardZdt⟦BpunktIndex⟧}þ],
      Blue, Point[{time⟦MaxcardZdt⟧, cardZdt⟦MaxcardZdt⟧}þ]}
]
```

Out[-]=

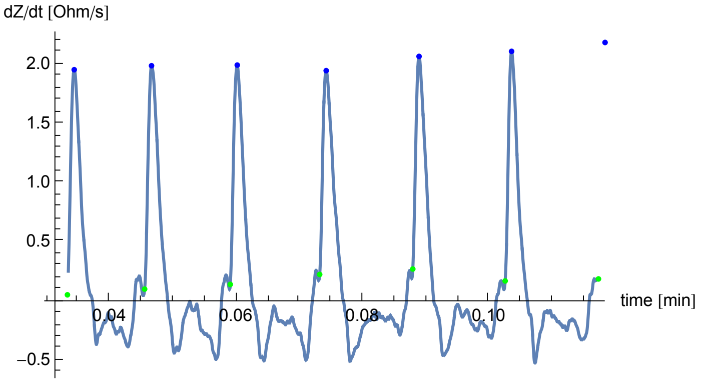

Minima dZ/dt for X-Point

For the X-point (purple) find the lowest minimum of cardiac dZ/dt within a time window defined by XtimeWindowStart and XtimeWindowEnd after the maximum of dZ/dt (blue).

In[ ]:=
```
XtimeWindowStart =0.150; (* in Seconds *)
XtimeWindowEnd =0.250; (* in Seconds *)
XpunktIndex=Table[
    section =cardZdt[[Min[MaxcardZdt[[i]]+XtimeWindowStart/samplingT, Length[cardZdt]];;
        Min[Round[MaxcardZdt[[i]]+XtimeWindowEnd/samplingT], Length[cardZdt]]]];
    peaks =FindPeaks[−section, 2, 0, −∞];
    Round[MaxcardZdt[[i]]+XtimeWindowStart/samplingT+Sort[peaks,#1[[2]]<#2[[2]]&][[−1, 1]]],
    {i, Length[MaxcardZdt]}];
```

In[ ]:=
```
ListLinePlot[
  {time[[imin ;; imax]], cardZdt[[imin ;; imax]]}þ,
  PlotRange →All, AxesLabel →{"time [min]","dZ/dt [Ohm/s]"},
  Epilog →{Purple, PointSize[0.01],Point[{time[[XpunktIndex]],cardZdt[[XpunktIndex]]}þ],
    Blue, Point[{time[[MaxcardZdt]], cardZdt[[MaxcardZdt]]}þ]}
]
```

Out[ ]=
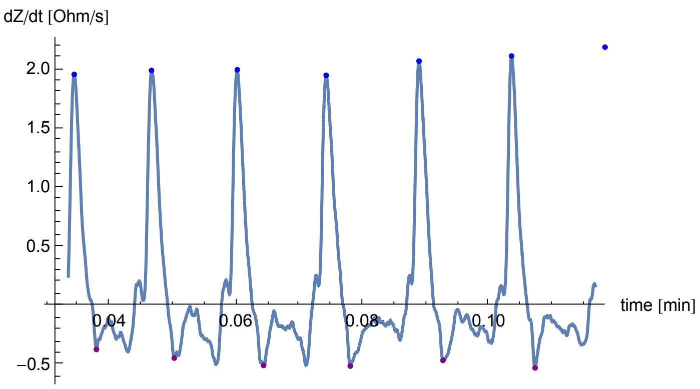

Plotting all Points

In[]:=
```
GraphicsGrid[{
    {ListPlot[{time, carZ}þ⟦istart;; iend⟧, FrameLabel →{"Time  [min]", "Cardiac Z [Ω]"},
        Frame →True,  Joined →True, ImageSize→Medium,
        Epilog →{Green,  PointSize[0.02], Point[{time⟦BpunktIndex⟧, carZ⟦BpunktIndex⟧}þ]}],
      ListPlot[{time, cardZdt}þ⟦istart;; iend⟧, FrameLabel →{"Time  [min]", "Cardiac dZ/dt [Ω/s]"},
        PlotRange→All,  Frame →True,  Joined →True,  ImageSize→Medium,
        Epilog →{Green,  PointSize[0.02], Point[{time⟦BpunktIndex⟧, cardZdt⟦BpunktIndex⟧}þ],
            Purple, Point[{time⟦XpunktIndex⟧, cardZdt⟦XpunktIndex⟧}þ]}],
      ListPlot[{time, card3Zdt3}þ⟦istart;; iend⟧, FrameLabel →{"Time  [min]", "Cardiac d²Z/dt² [Ω/s²]"},
        PlotRange→All,  Frame →True,  Joined →True,  ImageSize→Medium,
        Epilog →{Green,  PointSize[0.02], Point[{time⟦BpunktIndex⟧, card3Zdt3⟦BpunktIndex⟧}þ]}]},
    {ListPlot[{time, ECG}þ⟦istart;; iend⟧, FrameLabel →{"Time  [min]", "ECG [mV]"},
        Frame →True,  Joined →True,  PlotRange→All,  ImageSize→Medium,
        Epilog →{Red,  PointSize[0.01], Point[{time⟦RonsetIndex⟧, ECG⟦RonsetIndex⟧}þ],
            Blue, Point[{time⟦Minimad2ECGdt2⟧,  ECG⟦Minimad2ECGdt2⟧}þ]}],
      ListPlot[{time, dECGdt}þ⟦istart;; iend⟧, FrameLabel →{"Time  [min]", "dECG/dt [mV/s]"},
        PlotRange→All,  Frame →True,  Joined →True,  PlotRange→All,  ImageSize→Medium,
        Epilog →{Red,  PointSize[0.02], Point[{time⟦Minimad2ECGdt2⟧, dECGdt⟦Minimad2ECGdt2⟧}þ]}],
      ListPlot[{time, d2ECGdt2}þ⟦istart;; iend⟧, FrameLabel →{"Time  [min]", "d²ECG/dt² [mV/s²]"},
        PlotRange→All,  Frame →True,  Joined →True,  PlotRange→All,  ImageSize→Medium, Epilog→
          {Red, PointSize[0.02], Point[{time⟦Minimad2ECGdt2⟧, d2ECGdt2⟦Minimad2ECGdt2⟧}þ]}]}}
]
```

Out[]=

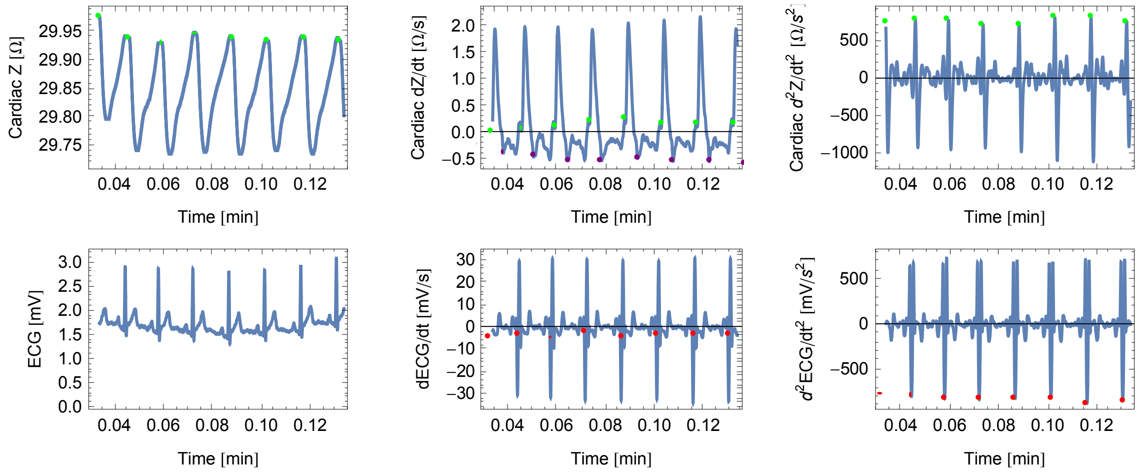

The following cell works only with Mathematica installed:

```
In[]:= length = 6000;
Manipulate[
  GraphicsGrid[{
      {ListPlot[{time, carZ}þ[[i;; i + length]], FrameLabel → {"Time [min]", "Cardiac Z [Ω]"},
          Frame → True, Joined → True, ImageSize → Medium,
          Epilog → {Green, PointSize[0.02], Point[{time[[BpunktIndex]], carZ[[BpunktIndex]]}þ]}],
        ListPlot[{time, cardZdt}þ[[i;; i + length]], FrameLabel → {"Time [min]", "Cardiac dZ/dt [Ω/s]"},
          PlotRange → All, Frame → True, Joined → True, ImageSize → Medium,
          Epilog → {Green, PointSize[0.02], Point[{time[[BpunktIndex]], cardZdt[[BpunktIndex]]}þ],
              Purple, Point[{time[[XpunktIndex]], cardZdt[[XpunktIndex]]}þ]}],
        ListPlot[{time, card3Zdt3}þ[[i;; i + length]], FrameLabel → {"Time [min]", "Cardiac d²Z/dt² [Ω/s²]"},
          PlotRange → All, Frame → True, Joined → True, ImageSize → Medium,
          Epilog → {Green, PointSize[0.02], Point[{time[[BpunktIndex]], card3Zdt3[[BpunktIndex]]}þ]}]},
      {ListPlot[{time, ECG}þ[[i;; i + length]], FrameLabel → {"Time [min]", "ECG [mV]"},
          Frame → True, Joined → True, PlotRange → All, ImageSize → Medium,
          Epilog → {Red, PointSize[0.01], Point[{time[[RonsetIndex]], ECG[[RonsetIndex]]}þ],
              Blue, Point[{time[[Minimad2ECGdt2]], ECG[[Minimad2ECGdt2]]}þ]}],
        ListPlot[{time, dECGdt}þ[[i;; i + length]], FrameLabel → {"Time [min]", "dECG/dt [mV/s]"},
          PlotRange → All, Frame → True, Joined → True,
          PlotRange → All, ImageSize → Medium, Epilog →
            {Red, PointSize[0.02], Point[{time[[Minimad2ECGdt2]], dECGdt[[Minimad2ECGdt2]]}þ]}],
        ListPlot[{time, d2ECGdt2}þ[[i;; i + length]], FrameLabel → {"Time [min]", "d²ECG/dt² [mV/s²]"},
          PlotRange → All, Frame → True, Joined → True,
          PlotRange → All, ImageSize → Medium, Epilog →
            {Red, PointSize[0.02], Point[{time[[Minimad2ECGdt2]], d2ECGdt2[[Minimad2ECGdt2]]}þ]}]}}
  ],
  {i, 1, Length[time] − length, length}, ControlPlacement → Top]
```

The algorithm should produce lists of equal length:

```
In[]:= Length[Minimad2ECGdt2]
Length[RonsetIndex]
Length[MaxcardZdt]
Length[XpunktIndex]
Length[BpunktIndex]

Out[]= 108

Out[]= 108

Out[]= 108

Out[]= 108

Out[]= 108
```

4. Calculating time differences

Lastly, PEP, LVET, and BMP are calculated from time differences.

In[ ]:=
```
PEP =(BpunktIndex −RonsetIndex)samplingT ;
LVET =(XpunktIndex −BpunktIndex)samplingT;
BPM  =60/(Differences[Minimad2ECGdt2]samplingT);
ListPlot[{{time⟦Minimad2ECGdt2⟧, PEP}þ,{time⟦Minimad2ECGdt2⟧, LVET}þ},PlotRange →{0,  0.5},
  PlotStyle →{Green,  Purple}, Frame →True,  FrameLabel →{"Zeit  [min]","PEP, LVET [s]"}]
```

Out[ ]=

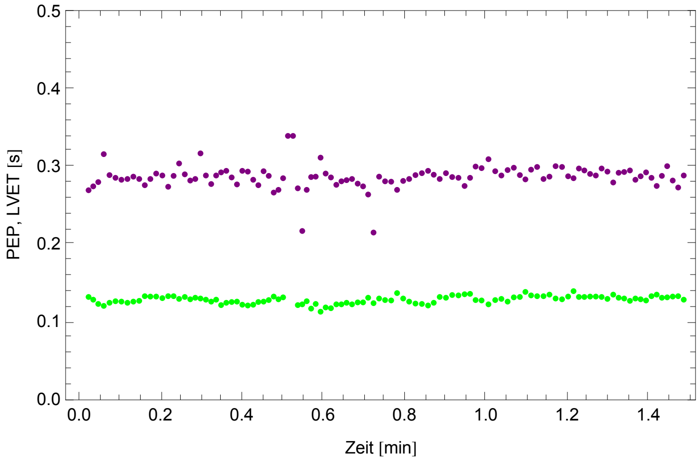

In[ ]:=
```
ListPlot [ { Drop[time⟦BpunktIndex⟧, −1], BPM}þ,
  PlotRange →{0,   120}, Frame →True,  FrameLabel→{"Zeit  [min]","BPM"}]
```

Out[ ]=

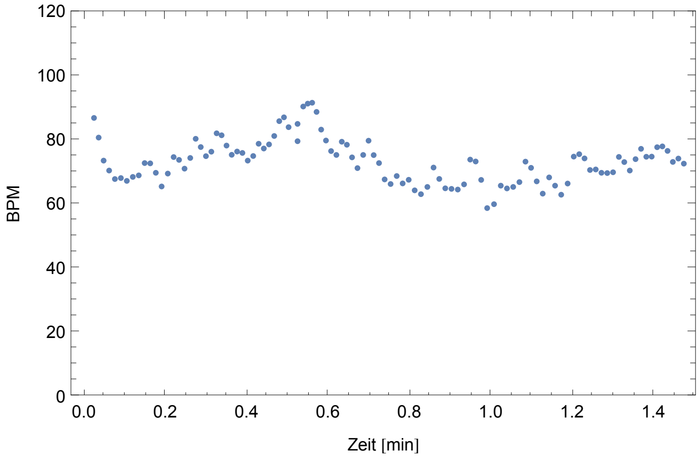

5. Export

PEP, LVET, and BMP can be exported as text files into the working directory. Rename "Example.txt" in order to change the output .

```
data ={Drop[time⟦BpunktIndex⟧, −1], Drop[PEP, −1], Drop[LVET, −1], BPM}þ;
Zeit =data⟦All, 1⟧;
Pep =data⟦All, 2⟧;
Lvet =data⟦All, 3⟧;
Bpm =data⟦All, 4⟧;
Export["Example.txt", {Zeit, Pep 1000, Lvet 1000, Bpm}þ]
```

Out[ ]= Example.dat
